# Supplementary material for: Perceived indoor temperature extremes are associated with sleep health among women in the United States
Source: medRxiv. 2025 Sep 14:2025.09.12.25335650. Preprint. [Version 1] doi: 10.1101/2025.09.12.25335650 (PMC12466784; doi:10.1101/2025.09.12.25335650)
Supplement: Supplement 1 [file media-1.pdf]

Perceived indoor temperature extremes are associated with sleep health among women in the United States

Symielle A. Gaston, PhD, MPH [1]

Dayna T. Neo, PhD, MPH [2]

W. Braxton Jackson II, MPH [2]

Dale P. Sandler, PhD [1]

Chandra L. Jackson, PhD, MS [1,3]

1 Epidemiology Branch, National Institute of Environmental Health Sciences, National Institutes of Health, Research Triangle Park, NC, USA

2 DLH LLC, Bethesda, MD, USA

3 Division of Intramural Research, National Institute on Minority Health and Health Disparities, National Institutes of Health, Bethesda, MD, USA

Please direct correspondence to: Dr. Chandra L. Jackson, PhD, MS, 111 TW Alexander Drive, Research Triangle Park, North Carolina, 27709, Phone: 984-287-3701, Fax: 301-480-3290, Email: Chandra.jackson@nih.gov

Supplemental Figure 1. Study population flow chart

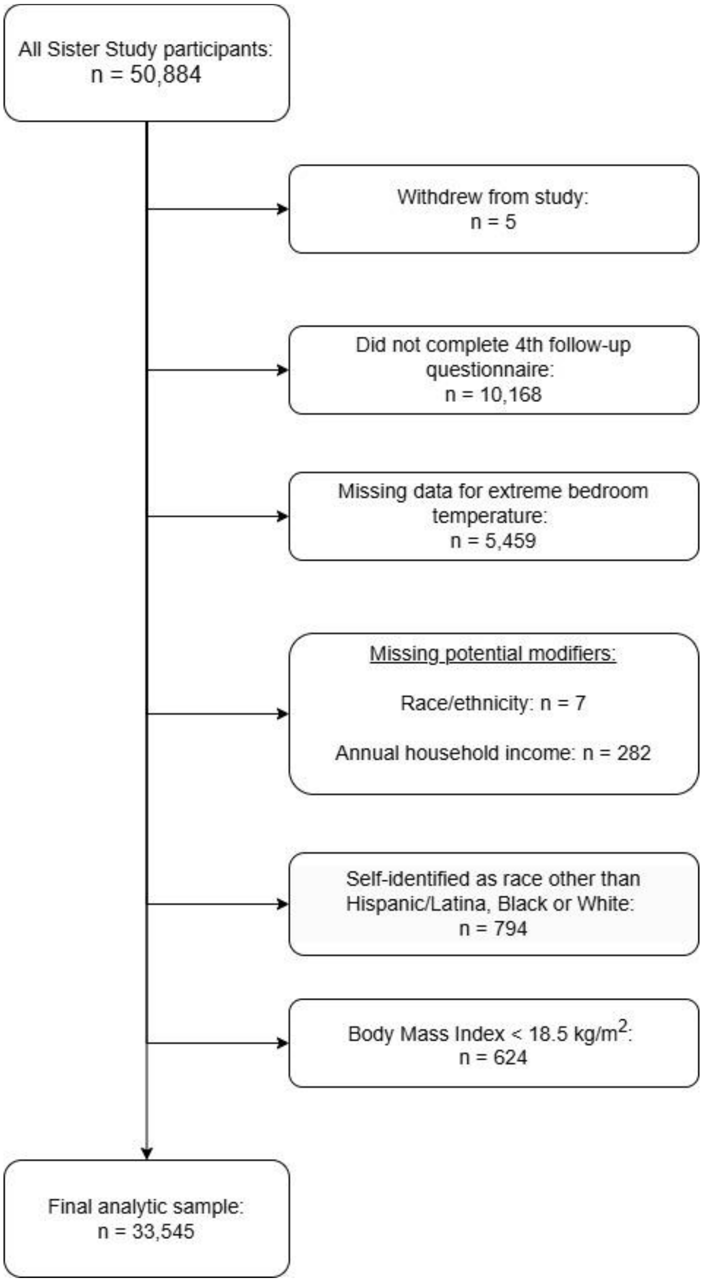

Supplemental Table 1. Comparison of sociodemographic and clinical characteristics at enrollment (2003-2009) between eligible (n = 33,545) and ineligible (n=17,339) Sister Study participants (N=50,884)

	Eligible n = 33,545 (65.9%)	Ineligible n = 17,339 (34.1%)	P-values
**Sociodemographic characteristics**			
Age, years (mean ± SD)	55.5 ± 8.6	56.0 ± 9.6	<0.001
Race and ethnicity			
Hispanic/Latina	1,225 (3.7)	1,290 (7.4)	<0.0001
Non-Hispanic Black	2,200 (6.6)	2,262 (13.1)	
Non-Hispanic White	30,120 (89.8)	12,438 (71.7)	
Other	0 (0.0)	1,334 (7.7)	
Missing	*0 (0.0)*	*15 (0.1)*	
Educational attainment			
≤High school or GED	4,462 (13.3)	3,342 (19.3)	<0.0001
Some college/technical	10,590 (31.6)	6,590 (38.0)	
≥ Bachelor's degree or higher	18,489 (55.1)	7,394 (42.6)	
Missing	*4 (0.01)*	*13 (0.1)*	
Annual household income			
<$20,000 - $49,999	7,095 (21.2)	5,485 (31.6)	<0.0001
$50,000-$99,999	13,601 (40.5)	6,303 (36.4)	
≥$100,000	11,913 (35.5)	4,487 (25.9)	
Missing	936 (2.8)	1,064 (6.1)	
Marital status			
Married or living as though married	25,855 (77.1)	12,137 (70.0)	<0.0001
Never married, divorced, widowed, or separated	7,686 (22.9)	5,187 (29.9)	
Missing	4 (0.0)	15 (0.1)	
Region of residence			
Northeast	5,894 (17.6)	2,621 (15.1)	<0.0001
Midwest	9,309 (27.8)	4,306 (24.8)	
South	10,559 (31.5)	6,284 (36.2)	
West	7,398 (22.1)	3,635 (21.0)	
Puerto Rico	385 (1.2)	488 (2.8)	
Missing	*0 (0.0)*	*5 (0.0)*	

Clinical characteristics			
Body mass index, kg/m^2			
Recommended (18.5 - 24.9)	13,718 (40.9)	5,910 (34.1)	<0.0001
Overweight (25.0 - 29.9)	10,582 (51.6)	5,311 (30.6)	
Obesity (≥30)	8,602 (25.6)	5,469 (31.5)	
Missing	*643 (1.9)*	*649 (3.7)*	
Menopausal status			
Premenopausal	11,328 (33.8)	5,700 (32.9)	0.04
Postmenopausal	22,210 (66.2)	11,633 (67.1)	
Missing or never had period	*7 (0.0)*	*6 (0.0)*	

Data presented as mean ± standard deviation or n (%)
Chi-square tests were used to compare categorical data.
A t-test was used to compare continuous age.

Supplemental Table 2. Sociodemographic characteristics, clinical characteristics, and sleep health dimensions among eligible Sister Study participants by race and ethnicity as well as perceived indoor temperature extremes, Sister Study, 2017-2019, N=33,545

**Race and ethnicity**	**Hispanic/Latina [a]**				**Non-Hispanic Black [b]**				**Non-Hispanic White [c]**			
	Total n = 1,225 (3.7%)	Infrequent temperature extremes [d] n = 1,042 (85.1%)	Too hot [e ¥] n = 112 (9.1%)	Too cold [f §] n = 46 (3.8%)	Total n = 2,200 (6.6%)	Infrequent temperature extremes [d] n = 1,802 (81.9%)	Too hot [e ¥] n = 324 (14.7%)	Too cold [f §] n = 34 (1.6%)	Total n = 30,120 (89.8%)	Infrequent temperature extremes [d] n = 26,783 (88.9%)	Too hot [e ¥] n = 2,582 (8.6%)	Too cold [f §] n = 382 (1.3%)
**Sociodemographic characteristics**												
Age, years (mean ± SD) [ǂ]	63.9 ± 8.5	64.2 ± 8.5	60.6 ± 7.8	65.9 ± 8.8	65.3 ± 8.2	65.9 ± 8.1	62.4 ± 7.7	66.0 ± 10.6	67.4 ± 8.5	67.9 ± 8.5	62.7 ± 7.6	69.1 ± 9.0
Age [ǂ]												
< 67.2 yrs	795 (64.9)	662 (63.5)	90 (80.4)	25 (54.4)	1,283 (58.3)	1,004 (55.7)	232 (71.6)	16 (47.1)	14,701 (48.8)	12,422 (46.4)	1,858 (72.0)	150 (39.3)
≥ 67.2 yrs	430 (35.1)	380 (36.5)	22 (19.6)	21 (45.7)	917 (41.7)	798 (44.3)	92 (28.4)	18 (52.9)	15,419 (51.2)	14,361 (53.6)	724 (28.0)	232 (60.7)
Educational attainment [g ǂ]												
<High school / GED	225 (18.4)	179 (17.2)	26 (23.2)	13 (28.3)	180 (8.2)	140 (7.8)	31 (9.6)	8 (23.5)	4,057 (13.5)	3,561 (13.3)	371 (14.4)	72 (18.9)
Some college / technical	394 (32.2)	329 (31.6)	38 (33.9)	15 (32.6)	686 (31.2)	548 (30.4)	110 (34.0)	12 (35.3)	9,510 (31.6)	8,369 (31.2)	870 (33.7)	141 (36.9)
≥ Bachelor's or higher	606 (49.5)	534 (51.3)	48 (42.9)	18 (39.1)	1,333 (60.6)	1,113 (61.8)	183 (56.5)	14 (41.2)	16,550 (54.9)	14,851 (55.5)	1,341 (51.9)	169 (44.2)
Missing	*0 (0.0)*	*0 (0.0)*	*0 (0.0)*	*0 (0.0)*	*1 (0.1)*	*1 (0.1)*	*0 (0.0)*	*0 (0.0)*	*3 (0.0)*	*2 (0.0)*	*0 (0.0)*	*0 (0.0)*
Annual household income [ǂ]												
<$20,000 - $49,999	580 (47.4)	483 (46.4)	44 (39.3)	37 (80.4)	756 (34.4)	632 (35.1)	91 (28.1)	18 (52.9)	7,562 (25.1)	6,832 (25.5)	504 (19.5)	141 (36.9)
$50,000-$99,999	351 (28.7)	304 (29.2)	34 (30.4)	6 (13.0)	839 (38.1)	675 (37.5)	141 (43.5)	7 (20.6)	11,187 (37.1)	10,031 (37.5)	896 (34.7)	121 (31.7)
≥$100,000	294 (24.0)	255 (24.5)	34 (30.4)	3 (6.5)	605 (27.5)	495 (27.5)	92 (28.4)	9 (26.5)	11,371 (37.8)	9,920 (37.0)	1,182 (45.8)	120 (31.4)
Marital status [ǂ]												
Married or living as though married	771 (62.9)	645 (61.9)	81 (72.3)	26 (56.5)	1,005 (45.7)	790 (43.8)	188 (58.0)	10 (29.4)	21,027 (69.8)	18,434 (68.8)	2,056 (79.6)	260 (68.1)
Never married, divorced, widowed, or separated	454 (37.1)	397 (38.1)	31 (27.7)	20 (43.5)	1,195 (54.3)	1,012 (56.2)	136 (42.0)	24 (70.6)	9,093 (30.2)	8,349 (31.2)	526 (20.4)	122 (31.9)
Region of residence [ǂ]												
Northeast	98 (8.0)	79 (7.6)	15 (13.4)	1 (2.2)	193 (8.8)	159 (8.8)	29 (9.0)	2 (5.9)	5,260 (17.5)	4,697 (17.5)	448 (17.4)	61 (16.0)
Midwest	77 (6.3)	70 (6.7)	7 (6.3)	0 (0.0)	451 (20.5)	377 (20.9)	61 (18.8)	7 (20.6)	8,457 (28.1)	7,520 (28.1)	724 (28.0)	101 (26.4)
South	349 (28.5)	306 (29.4)	30 (26.8)	8 (17.4)	1,379 (62.7)	1,113 (61.8)	216 (66.7)	20 (58.8)	9,370 (31.1)	8,354 (31.2)	782 (30.3)	119 (31.2)
West	335 (27.4)	290 (27.8)	33 (29.5)	6 (13.0)	177 (8.1)	153 (8.5)	18 (5.6)	5 (14.7)	7,029 (23.3)	6,208 (23.2)	628 (24.3)	101 (26.4)
Puerto Rico	366 (29.9)	297 (28.5)	27 (24.1)	31 (67.4)	0 (0.0)	0 (0.0)	0 (0.0)	0 (0.0)	4 (0.0)	4 (0.0)	0 (0.0)	0 (0.0)
**Clinical characteristics**												
Body Mass Index, kg/m^2 [ǂ]												
18.5 - 24.9	386 (31.5)	338 (32.4)	31 (27.7)	12 (26.1)	373 (17.0)	316 (17.5)	46 (14.2)	6 (17.7)	12,369 (41.1)	11,132 (41.6)	911 (35.3)	172 (45.0)
25.0 - 29.9	443 (36.2)	375 (36.0)	37 (33.0)	20 (43.5)	741 (33.7)	616 (34.2)	99 (30.6)	10 (29.4)	9,583 (31.8)	8,503 (31.8)	877 (34.0)	93 (24.4)
≥30	383 (31.3)	318 (30.5)	43 (38.4)	14 (30.4)	1,063 (48.3)	851 (47.2)	178 (54.9)	17 (50.0)	7,774 (25.8)	6,799 (25.4)	760 (29.4)	112 (29.3)
Missing	*13 (1.1)*	*11 (1.1)*	*1 (0.9)*	*0 (0.0)*	*23 (1.1)*	*19 (1.1)*	*1 (0.3)*	*1 (2.9)*	*394 (1.3)*	*349 (1.3)*	*34 (1.3)*	*5 (1.3)*
Menopausal status [ǂ]												
Premenopausal	87 (7.1)	79 (7.6)	7 (6.3)	1 (2.2)	143 (6.5)	104 (5.8)	29 (9.0)	5 (14.7)	1,116 (3.7)	927 (3.5)	147 (5.7)	19 (5.0)
Postmenopausal	1,138 (92.9)	963 (92.4)	105 (93.8)	45 (97.8)	2,055 (93.4)	1,696 (94.1)	295 (91.1)	29 (85.3)	29,000 (96.3)	25,852 (96.5)	2,435 (94.3)	363 (95.0)

Missing or never had a period	0 (0.0)	0 (0.0)	0 (0.0)	0 (0.0)	2 (0.1)	2 (0.1)	0 (0.0)	0 (0.0)	4 (0.0)	4 (0.0)	0 (0.0)	0 (0.0)
Trouble sleeping for any other reason [h ǂ]												
Yes	783 (63.9)	622 (59.7)	100 (89.3)	36 (78.3)	1,494 (67.9)	1,150 (63.8)	276 (85.2)	31 (91.2)	21,576 (71.6)	18,575 (69.4)	2,306 (89.3)	339 (88.7)
No	442 (36.1)	420 (40.3)	12 (10.7)	10 (21.7)	706 (32.1)	652 (36.2)	48 (14.8)	3 (8.8)	8,543 (28.4)	8,207 (30.6)	276 (10.7)	43 (11.3)
Missing	*0 (0.0)*	*0 (0.0)*	*0 (0.0)*	*0 (0.0)*	*0 (0.0)*	*0 (0.0)*	*0 (0.0)*	*0 (0.0)*	*1 (0.0)*	*1 (0.0)*	*0 (0.0)*	*0 (0.0)*
**Sleep health dimensions**												
Average weekly sleep duration [i ǂ]												
Short	191 (15.6)	160 (15.4)	20 (17.9)	6 (13.0)	628 (28.6)	504 (28.0)	92 (28.4)	14 (41.2)	3,004 (10.0)	2,618 (9.8)	289 (11.2)	56 (14.7)
Recommended	799 (65.2)	695 (66.7)	65 (58.0)	27 (58.7)	1,274 (57.9)	1,055 (58.6)	188 (58.0)	15 (44.1)	22,322 (74.1)	20,008 (74.7)	1,829 (70.8)	236 (61.8)
Long	196 (16.0)	157 (15.1)	24 (21.4)	8 (17.4)	257 (11.7)	212 (11.8)	36 (11.1)	4 (11.8)	4,470 (14.8)	3,865 (14.4)	448 (17.4)	79 (20.7)
Missing	*39 (3.2)*	*30 (2.9)*	*3 (2.7)*	*5 (10.9)*	*41 (1.9)*	*31 (1.7)*	*8 (2.5)*	*1 (2.9)*	*324 (1.1)*	*292 (1.1)*	*16 (0.6)*	*11 (2.9)*
Long sleep onset latency [j ǂ]												
Yes	216 (17.6)	146 (14)	41 (36.6)	14 (30.4)	327 (14.9)	210 (11.7)	90 (27.8)	7 (20.6)	3,640 (12.1)	2,841 (10.6)	558 (21.6)	99 (25.9)
No	968 (79.0)	864 (82.9)	67 (59.8)	28 (60.9)	1,834 (83.4)	1,559 (86.5)	229 (70.7)	26 (76.5)	26,150 (86.8)	23,642 (88.3)	2,006 (77.7)	277 (72.5)
Missing	*41 (3.4)*	*32 (3.1)*	*4 (3.6)*	*4 (8.7)*	*39 (1.8)*	*33 (1.8)*	*5 (1.5)*	*1 (2.9)*	*330 (1.1)*	*300 (1.1)*	*18 (0.7)*	*6 (1.6)*
Poor sleep maintenance [k ǂ]												
Yes	410 (33.5)	300 (28.8)	68 (60.7)	23 (50.0)	793 (36.1)	568 (31.5)	179 (55.3)	19 (55.9)	12,057 (40.0)	9,844 (36.8)	1,717 (66.5)	220 (57.6)
No	777 (63.4)	709 (68.0)	43 (38.4)	20 (43.5)	1,372 (62.4)	1,206 (66.9)	139 (42.9)	14 (41.2)	17,728 (58.9)	16,625 (62.1)	850 (32.9)	160 (41.9)
Missing	*38 (3.1)*	*33 (3.2)*	*1 (0.9)*	*3 (6.5)*	*35 (1.6)*	*28 (1.6)*	*6 (1.9)*	*1 (2.9)*	*335 (1.1)*	*314 (1.2)*	*15 (0.6)*	*2 (0.5)*
Insomnia symptoms [l ǂ]												
Yes	465 (38.0)	342 (32.8)	79 (70.5)	25 (54.4)	864 (39.3)	616 (34.2)	195 (60.2)	21 (61.8)	13,026 (43.3)	10,663 (39.8)	1,828 (70.8)	233 (61.0)
No	733 (59.8)	678 (65.1)	32 (28.6)	18 (39.1)	1,316 (59.8)	1,169 (64.9)	126 (38.9)	13 (38.2)	16,905 (56.1)	15,940 (59.5)	749 (29.0)	147 (38.5)
Missing	*27 (2.2)*	*22 (2.1)*	*1 (0.9)*	*3 (6.5)*	*20 (0.9)*	*17 (0.9)*	*3 (0.9)*	*0 (0.0)*	*189 (0.6)*	*180 (0.7)*	*5 (0.2)*	*2 (0.5)*
Sleep medication use [m ǂ]												
Yes	180 (14.7)	139 (13.3)	18 (16.1)	12 (26.1)	242 (11.0)	184 (10.2)	44 (13.6)	5 (14.7)	5,868 (19.5)	5,061 (18.9)	599 (23.2)	104 (27.2)
No	1,023 (83.5)	887 (85.1)	94 (83.9)	31 (67.4)	1,939 (88.1)	1,604 (89.0)	276 (85.2)	29 (85.3)	24,131 (80.1)	21,613 (80.7)	1,973 (76.4)	277 (72.5)
Missing	*22 (1.8)*	*16 (1.5)*	*0 (0.0)*	*3 (6.5)*	*19 (0.9)*	*14 (0.8)*	*4 (1.2)*	*0 (0.0)*	*121 (0.4)*	*109 (0.4)*	*10 (0.4)*	*1 (0.3)*
Daytime dysfunction [n]												
Yes	14 (1.1)	9 (0.9)	1 (0.9)	2 (4.4)	19 (0.9)	11 (0.6)	5 (1.5)	3 (8.8)	219 (0.7)	166 (0.6)	32 (1.2)	11 (2.9)
No	1,190 (97.1)	1,015 (97.4)	110 (98.2)	43 (93.5)	2,157 (98.1)	1,771 (98.3)	315 (97.2)	31 (91.2)	29,539 (98.1)	26,288 (98.2)	2,524 (97.8)	368 (96.3)
Missing	*21 (1.7)*	*18 (1.7)*	*1 (0.9)*	*1 (2.2)*	*24 (1.1)*	*20 (1.1)*	*4 (1.2)*	*0 (0.0)*	*362 (1.2)*	*329 (1.2)*	*26 (1.0)*	*3 (0.8)*
Healthcare provider diagnosed sleep apnea [o ǂ]												
Yes	100 (8.2)	76 (7.3)	13 (11.6)	8 (17.4)	302 (13.7)	229 (12.7)	54 (16.7)	10 (29.4)	3,051 (10.1)	2,649 (9.9)	301 (11.7)	58 (15.2)
No	1,107 (90.4)	953 (91.5)	99 (88.4)	36 (78.3)	1,857 (84.4)	1,539 (85.4)	264 (81.5)	23 (67.7)	26,767 (88.9)	23,863 (89.1)	2,265 (87.7)	314 (82.2)
Missing	*18 (1.5)*	*13 (1.3)*	*0 (0.0)*	*2 (4.4)*	*41 (1.9)*	*34 (1.9)*	*6 (1.9)*	*1 (2.9)*	*302 (1.0)*	*271 (1.0)*	*16 (0.6)*	*10 (2.6)*

Data presented as mean ± standard deviation or n (%)

Note: N=438 (1.3%) participants who reported frequent trouble sleeping due to both ‘too hot’ and ‘too cold’ temperatures were not included in the analysis.

Abbreviations: SD, Standard Deviation; GED, General Educational Development

[¥]Chi-square test p-values indicate significant differences between the overall prevalence of ‘too hot’ by race and ethnicity.

[§]Chi-square test p-values indicate significant differences between the overall prevalence of ‘too cold’ by race and ethnicity.

[ǂ]Chi-square or ANOVA test p-values indicate significant differences in characteristics between the total populations of each race and ethnicity.

[a] Excluded for reporting both 'Too hot' and 'Too cold', Hispanic/Latina: n = 25 (2.0%)
[b] Excluded for reporting both 'Too hot' and 'Too cold', Non-Hispanic Black: n = 40 (1.8%)
[c] Excluded for reporting both 'Too hot' and 'Too cold', Non-Hispanic White: n = 373 (1.2%)
[d] 'Infrequent temperature extremes' is defined as self-reported trouble sleeping due to feeling too hot < 3 times per week and trouble sleeping due to feeling too cold < 3 times per week in the past month.
[e] 'Too hot' is defined as self-reported trouble sleeping due to feeling too hot ≥ 3 times per week in the past month.
[f] 'Too cold' is defined as self-reported trouble sleeping due to feeling too cold ≥ 3 times per week in the past month.
[g] Educational attainment was assessed at baseline.
[h] Reasons for trouble sleeping ≥ 3 times per week in the past month include: unable to fall asleep within 30 minutes, waking up in the middle of the night or early morning, waking up to use the bathroom, cannot breathe comfortably, coughing or snoring loudly, having bad dreams, having pain, or other non-specified reasons.
[i] Sleep duration is based on reported bed and wake times or reported average sleep duration. Participants who reported ≤ 2 hours or ≥ 23 hours of sleep are excluded. Short: <7 hours; Recommended: 7-9 hours; Long >9 hours.
[j] Long sleep onset latency is defined as not falling asleep within 30 minutes at least three times a week during the past month.
[k] Poor sleep maintenance is defined as waking up in the middle of the night or early morning at least three times a week during the past month.
[l] Insomnia symptoms is defined as long sleep onset latency or poor sleep maintenance.
[m] Sleep medication use is defined as 'taking medicine (prescription or over the counter) to help you sleep' at least three times a week during the past month.
[n] Daytime dysfunction is defined as having trouble staying awake while driving, eating, or engaging in social activity at least three times a week during the past month.
[o] Healthcare provider diagnosed sleep apnea is defined as a current doctor or other health professional diagnosis of sleep apnea.

Supplemental Table 3. Sociodemographic characteristics, clinical characteristics, and sleep health dimensions among eligible Sister Study participants by annual household income as well as perceived indoor temperature extremes, Sister Study, 2017-2019, N = 33,545

**Annual household income**	**<$20,000 - $49,999 [a]**				**$50,000 - $99,999 [b]**				**≥ $100,000[c]**			
	Total n = 8,898 (26.5%)	Infrequent temperature extremes [d] n = 7,947 (89.3%)	Too hot [e ¥] n = 639 (7.2%)	Too cold [f §] n = 196 (2.2%)	Total n = 12,377 (36.9%)	Infrequent temperature extremes [d] n = 11,010 (89.0%)	Too hot [e ¥] n = 1,071 (8.7%)	Too cold [f §] n = 134 (1.1 %)	Total n = 12,270 (36.6%)	Infrequent temperature extremes [d] n = 10, 670 (87.0%)	Too hot [e ¥] n = 1,308 (10.7%)	Too cold [f §] n = 132 (1.1%)
**Sociodemographic characteristics**												
Age, years (mean ± SD) [ƚ]	70.5 ± 8.2	71.0 ± 8.1	65.2 ± 7.9	71.0 ± 9.0	67.8 ± 8.0	68.2 ± 8.0	63.7 ± 7.3	67.9 ± 8.4	64.0 ± 8.2	64.5 ±8.2	60.4 ± 7.1	65.5 ± 9.1
Age [ƚ]												
< 67.2 yrs	3,011 (33.8)	2,494 (31.4)	382 (59.8)	64 (32.7)	5,756 (46.5)	4,863 (44.2)	720 (67.2)	58 (43.3)	8,012 (65.3)	6,731 (63.1)	1,078 (82.4)	69 (52.3)
≥ 67.2 yrs	5,887 (66.2)	5,453 (68.6)	257 (40.2)	132 (67.4)	6,621 (53.5)	6,147 (55.8)	351 (32.8)	76 (56.7)	4,258 (34.7)	3,939 (36.9)	230 (17.6)	63 (47.7)
Race and ethnicity [ƚ]												
Hispanic/Latina	580 (6.5)	483 (6.1)	44 (6.9)	37 (18.9)	351 (2.8)	304 (2.8)	34 (3.2)	6 (4.5)	294 (2.4)	255 (2.4)	34 (2.6)	3 (2.3)
Non-Hispanic Black	756 (8.5)	632 (8.0)	91 (14.2)	18 (9.2)	839 (6.8)	675 (6.1)	141 (13.2)	7 (5.2)	605 (4.9)	495 (4.6)	92 (7.0)	9 (6.8)
Non-Hispanic White	7,562 (85.0)	6,832 (86.0)	504 (78.9)	141 (71.9)	11,187 (90.4)	10,031 (91.1)	896 (83.7)	121 (90.3)	11,371 (92.7)	9,920 (93.0)	1,182 (90.4)	120 (90.9)
Missing	*0 (0.0)*	*0 (0.0)*	*0 (0.0)*	*0 (0.0)*	*0 (0.0)*	*0 (0.0)*	*0 (0.0)*	*0 (0.0)*	*0 (0.0)*	*0 (0.0)*	*0 (0.0)*	*0 (0.0)*
Educational attainment [g ƚ]												
<High school / GED	2,160 (24.3)	1,893 (23.8)	176 (27.5)	61 (31.1)	1,615 (13.1)	1,412 (12.8)	159 (14.9)	21 (15.7)	687 (5.6)	575 (5.4)	93 (7.1)	11 (8.3)
Some college / technical	3,625 (40.7)	3,240 (40.8)	257 (40.2)	79 (40.3)	4,189 (33.9)	3,648 (33.1)	421 (39.3)	53 (39.6)	2,776 (22.6)	2,358 (22.1)	340 (26.0)	36 (27.3)
≥ Bachelor's or higher	3,111 (35.0)	2,812 (35.4)	206 (32.2)	56 (28.6)	6,572 (53.1)	5,949 (54.0)	491 (45.9)	60 (44.8)	8,806 (71.8)	7,737 (72.5)	875 (66.9)	85 (64.4)
Missing	*2 (0.0)*	*2 (0.0)*	*0 (0.0)*	*0 (0.0)*	*1 (0.0)*	*1 (0.0)*	*0 (0.0)*	*0 (0.0)*	*1 (0.0)*	*0 (0.0)*	*0 (0.0)*	*0 (0.0)*
Marital status [ƚ]												
Married or living as though married	3,393 (38.1)	2,966 (37.3)	303 (47.4)	78 (39.8)	8,645 (69.8)	7,585 (68.9)	834 (77.9)	101 (75.4)	10,765 (87.7)	9,318 (87.3)	1,188 (90.8)	117 (88.6)
Never married, divorced, widowed, or separated	5,505 (61.9)	4,981 (62.7)	336 (52.6)	118 (60.2)	3,732 (30.2)	3,425 (31.1)	237 (22.1)	33 (24.6)	1,505 (12.3)	1,352 (12.7)	120 (9.2)	15 (11.4)
Region of residence [ƚ]												
Northeast	1,246 (14.0)	1,127 (14.2)	89 (13.9)	20 (10.2)	1,935 (15.6)	1,761 (16.0)	134 (12.5)	22 (16.4)	2,370 (19.3)	2,047 (19.2)	269 (20.6)	22 (16.7)
Midwest	2,525 (28.4)	2,283 (28.7)	162 (25.4)	52 (26.5)	3,565 (28.8)	3,171 (28.8)	313 (29.2)	30 (22.4)	2,895 (23.6)	2,513 (23.6)	317 (24.2)	26 (19.7)
South	2,946 (33.1)	2,619 (33.0)	231 (36.2)	52 (26.5)	4,089 (33.0)	3,609 (32.8)	376 (35.1)	46 (34.3)	4,063 (33.1)	3,545 (33.2)	421 (32.2)	49 (37.1)
West	1,884 (21.2)	1,681 (21.2)	135 (21.1)	44 (22.5)	2,731 (22.1)	2,419 (22.0)	245 (22.9)	33 (24.6)	2,926 (23.9)	2,551 (23.9)	299 (22.9)	35 (26.5)
Puerto Rico	297 (3.3)	237 (3.0)	22 (3.4)	28 (14.3)	57 (0.5)	50 (0.5)	3 (0.3)	3 (2.2)	16 (0.1)	14 (0.1)	2 (0.2)	0 (0.0)
**Clinical characteristics**												
Body Mass Index, kg/m^2 [ƚ]												
18.5 - 24.9	2,854 (32.1)	2,605 (32.8)	158 (24.7)	65 (33.2)	4,679 (37.8)	4,220 (38.3)	338 (31.6)	62 (46.3)	5,595 (45.6)	4,961 (46.5)	492 (37.6)	63 (47.7)
25.0 - 29.9	2,852 (32.1)	2,546 (32.0)	211 (33.0)	57 (29.1)	4,064 (32.8)	3,633 (33.0)	354 (33.1)	30 (22.4)	3,851 (31.4)	3,315 (31.1)	448 (34.3)	36 (27.3)
≥30	3,067 (34.5)	2,681 (33.7)	265 (41.5)	71 (36.2)	3,469 (28.0)	3,014 (27.4)	363 (33.9)	41 (30.6)	2,684 (21.9)	2,273 (21.3)	353 (27.0)	31 (23.5)
Missing	*125 (1.4)*	*115 (1.5)*	*5 (0.8)*	*3 (1.5)*	*165 (1.3)*	*143 (1.3)*	*16 (1.5)*	*1 (0.8)*	*140 (1.1)*	*121 (1.1)*	*15 (1.2)*	*2 (1.5)*
Menopausal status [ƚ]												

Premenopausal	144 (1.6)	116 (1.5)	22 (3.4)	1 (0.5)	345 (2.8)	272 (2.5)	53 (5.0)	9 (6.7)	857 (7.0)	722 (6.8)	108 (8.3)	15 (11.4)
Postmenopausal	8,751 (98.4)	7,828 (98.5)	617 (96.6)	195 (99.5)	12,031 (97.2)	10,737 (97.5)	1,018 (95.1)	125 (93.3)	11,411 (93.0)	9,946 (93.2)	1,200 (91.7)	117 (88.6)
Missing or never had a period	*3 (0.0)*	*3 (0.0)*	*0 (0.0)*	*0 (0.0)*	*1 (0.0)*	*1 (0.0)*	*0 (0.0)*	*0 (0.0)*	*2 (0.0)*	*2 (0.0)*	*0 (0.0)*	*0 (0.0)*
Trouble sleeping for any other reason [h ǂ]												
Yes	6,614 (74.3)	5,741 (72.2)	585 (91.6)	176 (89.8)	8,820 (71.3)	7,605 (69.1)	943 (88.1)	119 (88.8)	8,419 (68.6)	7,001 (65.6)	1,154 (88.2)	111 (84.1)
No	2,284 (25.7)	2,206 (27.8)	54 (8.5)	20 (10.2)	3,556 (28.7)	3,404 (30.9)	128 (12.0)	15 (11.2)	3,851 (31.4)	3,669 (34.4)	154 (11.8)	21 (15.9)
Missing	*0 (0.0)*	*0 (0.0)*	*0 (0.0)*	*0 (0.0)*	*1 (0.0)*	*1 (0.0)*	*0 (0.0)*	*0 (0.0)*	*0 (0.0)*	*0 (0.0)*	*0 (0.0)*	*0 (0.0)*
**Sleep health dimensions**												
Average weekly sleep duration [i ǂ]												
Short	1,186 (13.3)	1,035 (13.0)	93 (14.6)	36 (18.4)	1,390 (11.2)	1,184 (10.8)	161 (15.0)	22 (16.4)	1,247 (10.2)	1,063 (10.0)	147 (11.2)	18 (13.6)
Recommended	5,902 (66.3)	5,345 (67.3)	399 (62.4)	104 (53.1)	9,070 (73.3)	8,144 (74.0)	732 (68.4)	91 (67.9)	9,423 (76.8)	8,269 (77.5)	951 (72.7)	83 (62.9)
Long	1,615 (18.2)	1,403 (17.7)	134 (21.0)	44 (22.5)	1,801 (14.6)	1,576 (14.3)	171 (16.0)	19 (14.2)	1,507 (12.3)	1,255 (11.8)	203 (15.5)	28 (21.2)
Missing	*195 (2.2)*	*164 (2.1)*	*13 (2.0)*	*12 (6.1)*	*116 (0.9)*	*106 (1.0)*	*7 (0.7)*	*2 (1.5)*	*93 (0.8)*	*83 (0.8)*	*7 (0.5)*	*3 (2.3)*
Long sleep onset latency [j ǂ]												
Yes	1,420 (16.0)	1,106 (13.9)	192 (30.1)	64 (32.7)	1,561 (12.6)	1,186 (10.8)	274 (25.6)	31 (23.1)	1,202 (9.8)	905 (8.5)	223 (17.1)	25 (18.9)
No	7,313 (82.2)	6,697 (84.3)	436 (68.2)	125 (63.8)	10,685 (86.3)	9,702 (88.1)	791 (73.9)	102 (76.1)	10,954 (89.3)	9,666 (90.6)	1,075 (82.2)	104 (78.8)
Missing	*165 (1.9)*	*144 (1.8)*	*11 (1.7)*	*7 (3.6)*	*131 (1.1)*	*122 (1.1)*	*6 (0.6)*	*1 (0.8)*	*114 (0.9)*	*99 (0.9)*	*10 (0.8)*	*3 (2.3)*
Poor sleep maintenance [k ǂ]												
Yes	3,634 (40.8)	3,011 (37.9)	426 (66.7)	112 (57.1)	4,832 (39.0)	3,957 (35.9)	677 (63.2)	82 (61.2)	4,794 (39.1)	3,744 (35.1)	861 (65.8)	68 (51.5)
No	5,109 (57.4)	4,791 (60.3)	211 (33.0)	79 (40.3)	7,406 (59.8)	6,925 (62.9)	386 (36.0)	51 (38.1)	7,362 (60.0)	6,824 (64.0)	435 (33.3)	64 (48.5)
Missing	*155 (1.7)*	*145 (1.8)*	*2 (0.3)*	*5 (2.6)*	*139 (1.1)*	*128 (1.2)*	*8 (0.8)*	*1 (0.8)*	*114 (0.9)*	*102 (1)*	*12 (0.9)*	*0 (0.0)*
Insomnia symptoms [l ǂ]												
Yes	3,961 (44.5)	3,288 (41.4)	458 (71.7)	120 (61.2)	5,263 (42.5)	4,316 (39.2)	731 (68.3)	85 (63.4)	5,131 (41.8)	4,017 (37.7)	913 (69.8)	74 (56.1)
No	4,850 (54.5)	4,580 (57.6)	180 (28.2)	72 (36.7)	7,031 (56.8)	6,615 (60.1)	337 (31.5)	48 (35.8)	7,073 (57.6)	6,592 (61.8)	390 (29.8)	58 (43.9)
Missing	*87 (1.0)*	*79 (1.0)*	*1 (0.2)*	*4 (2.0)*	*83 (0.7)*	*79 (0.7)*	*3 (0.3)*	*1 (0.8)*	*66 (0.5)*	*61 (0.6)*	*5 (0.4)*	*0 (0.0)*
Sleep medication use [m ǂ]												
Yes	1,839 (20.7)	1,571 (19.8)	170 (26.6)	57 (29.1)	2,370 (19.2)	2,053 (18.7)	242 (22.6)	27 (20.2)	2,081 (17.0)	1,760 (16.5)	249 (19.0)	37 (28.0)
No	6,984 (78.5)	6,312 (79.4)	465 (72.8)	136 (69.4)	9,955 (80.4)	8,913 (81.0)	822 (76.8)	106 (79.1)	10,154 (82.8)	8,879 (83.2)	1,056 (80.7)	95 (72.0)
Missing	*75 (0.8)*	*64 (0.8)*	*4 (0.6)*	*3 (1.5)*	*52 (0.4)*	*44 (0.4)*	*7 (0.7)*	*1 (0.8)*	*35 (0.3)*	*31 (0.3)*	*3 (0.2)*	*0 (0.0)*
Daytime dysfunction [n ǂ]												
Yes	89 (1.0)	69 (0.9)	11 (1.7)	5 (2.6)	87 (0.7)	63 (0.6)	14 (1.3)	7 (5.2)	76 (0.6)	54 (0.5)	13 (1.0)	4 (3.0)
No	8,677 (97.5)	7,755 (97.6)	623 (97.5)	189 (96.4)	12,136 (98.1)	10,810 (98.2)	1,042 (97.3)	126 (94.0)	12,073 (98.4)	10,509 (98.5)	1,284 (98.2)	127 (96.2)
Missing	*132 (1.5)*	*123 (1.6)*	*5 (0.8)*	*2 (1.0)*	*154 (1.2)*	*137 (1.2)*	*15 (1.4)*	*1 (0.8)*	*121 (1.0)*	*107 (1.0)*	*11 (0.8)*	*1 (0.8)*
Healthcare provider diagnosed sleep apnea [o ǂ]												
Yes	1,156 (13.0)	998 (12.6)	106 (16.6)	36 (18.4)	1,300 (10.5)	1,114 (10.1)	136 (12.7)	22 (16.4)	997 (8.1)	842 (7.9)	126 (9.6)	18 (13.6)
No	7,595 (85.4)	6,824 (85.9)	523 (81.9)	153 (78.1)	10,947 (88.5)	9,778 (88.8)	931 (86.9)	106 (79.1)	11,189 (91.2)	9,753 (91.4)	1,174 (89.8)	114 (86.4)
Missing	*147 (1.7)*	*125 (1.6)*	*10 (1.6)*	*7 (3.6)*	*130 (1.1)*	*118 (1.1)*	*4 (0.4)*	*6 (4.5)*	*84 (0.7)*	*75 (0.7)*	*8 (0.6)*	*0 (0.0)*

Data presented as mean ± standard deviation or n (%)

Note: N=438 (1.3%) participants who reported both 'too hot' and 'too cold' temperatures were not included in the analysis.

Abbreviations: SD, Standard Deviation; GED, General Educational Development

[¥] Chi-square test p-values indicate significant differences in the overall prevalence of ‘too hot’ by annual household income category.
[§] Chi-square test p-values indicate significant differences in the overall prevalence of ‘too cold’ by annual household income category.
[ƚ] Chi-square or ANOVA test p-values indicate significant differences in characteristics between the total populations of each annual household income category.
[a] Excluded for reporting both ‘Too hot’ and ‘Too cold’, < $20,000 - $49,999: n = 116 (1.3%)
[b] Excluded for reporting both ‘Too hot’ and ‘Too cold’, $50,000 - $99,999: n = 162 (1.3%)
[c] Excluded for reporting both ‘Too hot’ and ‘Too cold’, ≥ $100,000: n = 160 (1.3%)
[d] Infrequent temperature extremes’ is defined as self-reported trouble sleeping due to feeling too hot < 3 times per week and trouble sleeping due to feeling too cold < 3 times per week in the past month.
[e] ’Too hot’ is defined as self-reported trouble sleeping due to feeling too hot ≥ 3 times per week in the past month.
[f] ’Too cold’ is defined as self-reported trouble sleeping due to feeling too cold ≥ 3 times per week in the past month.
[g] Educational attainment was assessed at baseline.
[h] Reasons for trouble sleeping ≥ 3 times per week in the past month include: unable to fall asleep within 30 minutes, waking up in the middle of the night or early morning, waking up to use the bathroom, cannot breathe comfortably, coughing or snoring loudly, having bad dreams, having pain, or other non-specified reasons.
[i] Sleep duration is based on reported bed and wake times or reported average sleep duration. Participants who reported ≤ 2 hours or ≥ 23 hours of sleep are excluded. Short: <7 hours; Recommended: 7-9 hours; Long >9 hours
[j] Long sleep onset latency is defined as not falling asleep within 30 minutes at least three times a week during the past month.
[k] Poor sleep maintenance is defined as waking up in the middle of the night or early morning at least three times a week during the past month.
[l] Insomnia symptoms is defined as having long sleep onset latency or poor sleep maintenance.
[m] Sleep medication use is defined as 'taking medicine (prescription or over the counter) to help you sleep' at least three times a week during the past month.
[n] Daytime dysfunction is defined as having trouble staying awake while driving, eating, or engaging in social activity at least three times a week during the past month.
[o] Healthcare provider diagnosed sleep apnea is defined as a current doctor or other health professional diagnosis of sleep apnea.

Supplemental Table 4. Sociodemographic characteristics, clinical characteristics, and sleep health dimensions among eligible Sister Study participants by region of residence as well as perceived indoor temperature extremes, Sister Study, 2017-2019, N = 33,545

Region	Northeast [a]				Midwest [b]				South [c]				West [d]				Puerto Rico [e]			
	Total n = 5,551 (16.6%)	Infrequent temperature extremes [f] n = 4,935 (88.9%)	Too hot[g¥] n = 492 (8.9%)	Too cold[h§] n = 64 (1.2%)	Total n = 8,985 (26.8%)	Infrequent temperature extremes [f] n = 7,967 (88.7%)	Too hot[g¥] n = 792 (8.8%)	Too cold[h§] n = 108 (1.2%)	Total n = 11,098 (33.1%)	Infrequent temperature extremes [f] n = 9,773 (88.1%)	Too hot[g¥] n = 1,028 (9.3%)	Too cold[h§] n = 147 (1.3%)	Total n = 7,541 (22.5%)	Infrequent temperature extremes [f] n = 6,651 (88.2%)	Too hot[g¥] n = 679 (9.0%)	Too cold[h§] n = 112 (1.5%)	Total n = 370 (1.1%)	Infrequent temperature extremes [f] n = 301 (81.4%)	Too hot[g¥] n = 27 (7.3%)	Too cold [h§] n = 31 (8.4%)
**Sociodemographic characteristics**																				
Age, years (mean ± SD) [ǂ]	66.7 ± 8.7	67.3 ± 8.6	61.5 ± 7.3	67.9 ± 8.6	66.8 ± 8.5	67.3 ± 8.5	62.1 ± 7.4	68.0 ± 9.3	67.2 ± 8.5	67.7 ± 8.4	62.9 ± 7.7	68.4 ± 9.7	67.7 ± 8.5	68.2 ± 8.4	63.5 ± 7.8	70.0 ± 8.7	65.8 ± 8.2	65.9 ± 8.2	64.3 ± 7.5	66.8 ± 8.3
Age [ǂ]																				
< 67.2 yrs	2,898 (52.2)	2,434 (49.3)	389 (79.1)	26 (40.6)	4,630 (51.5)	3,896 (48.9)	597 (75.4)	48 (44.4)	5,480 (49.4)	4,590 (47.0)	717 (69.8)	63 (42.9)	3,567 (47.3)	3,002 (45.1)	460 (67.8)	38 (33.9)	204 (55.1)	166 (55.2)	17 (63.0)	16 (51.6)
≥ 67.2 yrs	2,653 (47.8)	2,501 (50.7)	103 (20.9)	38 (59.4)	4,355 (48.5)	4,071 (51.1)	195 (24.6)	60 (55.6)	5,618 (50.6)	5,183 (53.0)	311 (30.3)	84 (57.1)	3,974 (52.7)	3,649 (54.9)	219 (32.3)	74 (66.1)	166 (44.9)	135 (44.9)	10 (37.0)	15 (48.4)
Race and ethnicity [ǂ]																				
Hispanic/Latina	98 (1.8)	79 (1.6)	15 (3.1)	1 (1.6)	77 (0.9)	70 (0.9)	7 (0.9)	0 (0.0)	349 (3.1)	306 (3.1)	30 (2.9)	8 (5.4)	335 (4.4)	290 (4.4)	33 (4.9)	6 (5.4)	366 (98.9)	297 (98.7)	27 (100.0)	31 (100.0)
Non-Hispanic Black	193 (3.5)	159 (3.2)	29 (5.9)	2 (3.1)	451 (5.0)	377 (4.7)	61 (7.7)	7 (6.5)	1,379 (12.4)	1,113 (11.4)	216 (21.0)	20 (13.6)	177 (2.4)	153 (2.3)	18 (2.7)	5 (4.5)	4 (1.1)	0 (0.0)	0 (0.0)	0 (0.0)
Non-Hispanic White	5,260 (94.8)	4,697 (95.2)	448 (91.1)	61 (95.3)	8,457 (94.1)	7,520 (94.4)	724 (91.4)	101 (93.5)	9,370 (84.4)	8,354 (85.5)	782 (76.1)	119 (81.0)	7,029 (93.2)	6,208 (93.3)	628 (92.5)	101 (90.2)	4 (1.1)	4 (1.3)	0 (0.0)	0 (0.0)
Educational attainment [i ǂ]																				
<High school / GED	750 (13.5)	656 (13.3)	71 (14.4)	15 (23.4)	1,459 (16.2)	1,277 (16.0)	143 (18.1)	24 (22.2)	1,399 (12.6)	1,229 (12.6)	123 (12.0)	30 (20.4)	771 (10.2)	658 (9.9)	80 (11.8)	16 (14.3)	83 (22.4)	60 (19.9)	11 (40.7)	8 (25.8)
Some college / technical	1,541 (27.8)	1,349 (27.3)	149 (30.3)	22 (34.4)	2,997 (33.4)	2,643 (33.2)	262 (33.1)	46 (42.6)	3,507 (31.6)	3,047 (31.2)	354 (34.4)	52 (35.4)	2,446 (32.4)	2,130 (32.0)	246 (36.2)	38 (33.9)	99 (26.8)	77 (25.6)	7 (25.9)	10 (32.3)
≥ Bachelor's or higher	3,258 (58.7)	2,929 (59.4)	272 (55.3)	27 (42.2)	4,529 (50.4)	4,047 (50.8)	387 (48.9)	38 (35.2)	6,190 (55.8)	5,495 (56.2)	551 (53.6)	65 (44.2)	4,324 (57.3)	3,863 (58.1)	353 (52.0)	58 (51.8)	188 (50.8)	164 (54.5)	9 (33.3)	13 (41.9)
Missing	*2 (0.0)*	*1 (0.0)*	*0 (0.0)*	*0 (0.0)*	*0 (0.0)*	*0 (0.0)*	*0 (0.0)*	*0 (0.0)*	*2 (0.0)*	*2 (0.0)*	*0 (0.0)*	*0 (0.0)*	*0 (0.0)*	*0 (0.0)*	*0 (0.0)*	*0 (0.0)*	*0 (0.0)*	*0 (0.0)*	*0 (0.0)*	*0 (0.0)*
Annual household income [ǂ]																				
< $20,000 - $49,999	1,246 (22.5)	1,127 (22.8)	89 (18.1)	20 (31.3)	2,525 (28.1)	2,283 (28.7)	162 (20.5)	52 (48.2)	2,946 (26.6)	2,619 (26.8)	231 (22.5)	52 (35.4)	1,884 (25.0)	1,681 (25.3)	135 (19.9)	44 (39.3)	297 (80.3)	237 (78.7)	22 (81.5)	28 (90.3)
$50,000 – $99,999	1,935 (34.9)	1,761 (35.7)	134 (27.2)	22 (34.4)	3,565 (39.7)	3,171 (39.8)	313 (39.5)	30 (27.8)	4,089 (36.8)	3,609 (36.9)	376 (36.6)	46 (31.3)	2,731 (36.2)	2,419 (36.4)	245 (36.1)	33 (29.5)	57 (15.4)	50 (16.6)	3 (11.1)	3 (9.7)
≥ $100,000	2,370 (42.7)	2,047 (41.5)	269 (54.7)	22 (34.4)	2,895 (32.2)	2,513 (31.5)	317 (40.0)	26 (24.1)	4,063 (36.6)	3,545 (36.3)	421 (41.0)	49 (33.3)	2,926 (38.8)	2,551 (38.4)	299 (44.0)	35 (31.3)	16 (4.3)	14 (4.7)	2 (7.4)	0 (0.0)
Marital status [ǂ]																				
Married/living as though married	3,805 (68.5)	3,327 (67.4)	387 (78.7)	47 (73.4)	6,277 (69.9)	5,497 (69.0)	623 (78.7)	70 (64.8)	7,426 (66.9)	6,459 (66.1)	774 (75.3)	94 (63.9)	5,071 (67.2)	4,405 (66.2)	523 (77.0)	68 (60.7)	224 (60.5)	181 (60.1)	18 (66.7)	17 (54.8)
Never married, divorced, widowed, or separated	1,746 (31.5)	1,608 (32.6)	105 (21.3)	17 (26.6)	2,708 (30.1)	2,470 (31.0)	169 (21.3)	38 (35.2)	3,672 (33.1)	3,314 (33.9)	254 (24.7)	53 (36.1)	2,470 (32.8)	2,246 (33.8)	156 (23.0)	44 (39.3)	146 (39.5)	120 (39.9)	9 (33.3)	14 (45.2)
**Clinical characteristics**																				
Body Mass Index, kg/m^2 [ǂ]																				
18.5 - 24.9	2,300 (41.4)	2,080 (42.2)	168 (34.2)	23 (35.9)	3,224 (35.9)	2,889 (36.3)	251 (31.7)	44 (40.7)	4,159 (37.5)	3,767 (38.5)	290 (28.2)	55 (37.4)	3,333 (44.2)	2,961 (44.5)	269 (39.6)	59 (52.7)	112 (30.3)	89 (29.6)	10 (37.0)	9 (29.0)
25.0 - 29.9	1,745 (31.4)	1,556 (31.5)	154 (31.3)	17 (26.6)	2,915 (32.4)	2,572 (32.3)	272 (34.3)	30 (27.8)	3,619 (32.6)	3,182 (32.6)	349 (34.0)	38 (25.9)	2,339 (31.0)	2,061 (31.0)	229 (33.7)	25 (22.3)	149 (40.3)	123 (40.9)	9 (33.3)	13 (41.9)

≥30	1,434 (25.8)	1,235 (25.0)	164 (33.3)	23 (35.9)	2,738 (30.5)	2,410 (30.3)	257 (32.5)	34 (31.5)	3,163 (28.5)	2,686 (27.5)	378 (36.8)	51 (34.7)	1,780 (23.6)	1,551 (23.3)	174 (25.6)	26 (23.2)	105 (28.4)	86 (28.6)	8 (29.6)	9 (29.0)
Missing	*72 (1.3)*	*64 (1.3)*	*6 (1.2)*	*1 (1.6)*	*108 (1.2)*	*96 (1.2)*	*12 (1.5)*	*0 (0.0)*	*157 (1.4)*	*138 (1.4)*	*11 (1.1)*	*3 (2.0)*	*89 (1.2)*	*78 (1.2)*	*7 (1.0)*	*2 (1.8)*	*4 (1.1)*	*3 (1.0)*	*0 (0.0)*	*0 (0.0)*
Menopausal status																				
Premenopausal	230 (4.1)	192 (3.9)	31 (6.3)	2 (3.1)	376 (4.2)	314 (3.9)	50 (6.3)	7 (6.5)	437 (3.9)	350 (3.6)	65 (6.3)	10 (6.8)	282 (3.7)	235 (3.5)	36 (5.3)	5 (4.5)	21 (5.7)	19 (6.3)	1 (3.7)	1 (3.2)
Postmenopausal	5,321 (95.9)	4,743 (96.1)	461 (93.7)	62 (96.9)	8,608 (95.8)	7,652 (96.1)	742 (93.7)	101 (93.5)	10,657 (96.0)	9,419 (96.4)	963 (93.7)	137 (93.2)	7,258 (96.3)	6,415 (96.5)	643 (94.7)	107 (95.5)	349 (94.3)	282 (93.7)	26 (96.3)	30 (96.8)
Missing or never had a period	*0 (0.0)*	*0 (0.0)*	*0 (0.0)*	*0 (0.0)*	*1 (0.0)*	*1 (0.0)*	*0 (0.0)*	*0 (0.0)*	*4 (0.0)*	*4 (0.0)*	*0 (0.0)*	*0 (0.0)*	*1 (0.0)*	*1 (0.0)*	*0 (0.0)*	*0 (0.0)*	*0 (0.0)*	*0 (0.0)*	*0 (0.0)*	*0 (0.0)*
Trouble sleeping for any other reason [j] ǂ																				
Yes	3,876 (69.8)	3,334 (67.6)	430 (87.4)	55 (85.9)	6,290 (70.0)	5,375 (67.5)	702 (88.6)	101 (93.5)	7,916 (71.3)	6,722 (68.8)	921 (89.6)	131 (89.1)	5,522 (73.2)	4,725 (71.0)	605 (89.1)	96 (85.7)	249 (67.3)	191 (63.5)	24 (88.9)	23 (74.2)
No	1,675 (30.2)	1,601 (32.4)	62 (12.6)	9 (14.1)	2,695 (30.0)	2,592 (32.5)	90 (11.4)	7 (6.5)	3,181 (28.7)	3,050 (31.2)	107 (10.4)	16 (10.9)	2,019 (26.8)	1,926 (29.0)	74 (10.9)	16 (14.3)	121 (32.7)	110 (36.5)	3 (11.1)	8 (25.8)
Missing	*0 (0.0)*	*0 (0.0)*	*0 (0.0)*	*0 (0.0)*	*0 (0.0)*	*0 (0.0)*	*0 (0.0)*	*0 (0.0)*	*1 (0.0)*	*1 (0.0)*	*0 (0.0)*	*0 (0.0)*	*0 (0.0)*	*0 (0.0)*	*0 (0.0)*	*0 (0.0)*	*0 (0.0)*	*0 (0.0)*	*0 (0.0)*	*0 (0.0)*
**Sleep health dimensions**																				
Average weekly sleep duration [k] ǂ																				
Short	709 (12.8)	624 (12.6)	61 (12.4)	17 (26.6)	944 (10.5)	792 (9.9)	119 (15.0)	17 (15.7)	1,369 (12.3)	1,172 (12.0)	140 (13.6)	26 (17.7)	743 (9.9)	648 (9.7)	75 (11.1)	12 (10.7)	58 (15.7)	46 (15.3)	6 (22.2)	4 (12.9)
Recommended	4,005 (72.2)	3,571 (72.4)	357 (72.6)	35 (54.7)	6,657 (74.1)	5,974 (75.0)	542 (68.4)	71 (65.7)	7,912 (71.3)	7,033 (72.0)	703 (68.4)	87 (59.2)	5,590 (74.1)	4,980 (74.9)	472 (69.5)	67 (59.8)	231 (62.4)	200 (66.5)	8 (29.6)	18 (58.1)
Long	765 (13.8)	673 (13.6)	71 (14.4)	10 (15.6)	1,273 (14.2)	1,100 (13.8)	126 (15.9)	16 (14.8)	1,693 (15.3)	1,463 (15.0)	174 (16.9)	29 (19.7)	1,131 (15.0)	957 (14.4)	127 (18.7)	30 (26.8)	61 (16.5)	41 (13.6)	10 (37.0)	6 (19.4)
Missing	*72 (1.3)*	*67 (1.4)*	*3 (0.6)*	*2 (3.1)*	*111 (1.2)*	*101 (1.3)*	*5 (0.6)*	*4 (3.7)*	*124 (1.1)*	*105 (1.1)*	*11 (1.1)*	*5 (3.4)*	*77 (1.0)*	*66 (1.0)*	*5 (0.7)*	*3 (2.7)*	*20 (5.4)*	*14 (4.7)*	*3 (11.1)*	*3 (9.7)*
Long sleep onset latency [l] ǂ																				
Yes	634 (11.4)	499 (10.1)	102 (20.7)	13 (20.3)	1,064 (11.8)	822 (10.3)	161 (20.3)	34 (31.5)	1,501 (13.5)	1,126 (11.5)	278 (27)	34 (23.1)	910 (12.1)	700 (10.5)	141 (20.8)	28 (25.0)	74 (20.0)	50 (16.6)	7 (25.9)	11 (35.5)
No	4,846 (87.3)	4,373 (88.6)	384 (78.1)	49 (76.6)	7,832 (87.2)	7,063 (88.7)	627 (79.2)	73 (67.6)	9,457 (85.2)	8,521 (87.2)	741 (72.1)	111 (75.5)	6,545 (86.8)	5,872 (88.3)	534 (78.7)	82 (73.2)	272 (73.5)	236 (78.4)	16 (59.3)	16 (51.6)
Missing	*71 (1.3)*	*63 (1.3)*	*6 (1.2)*	*2 (3.1)*	*89 (1.0)*	*82 (1.0)*	*4 (0.5)*	*1 (0.9)*	*140 (1.3)*	*126 (1.3)*	*9 (0.9)*	*2 (1.4)*	*86 (1.1)*	*79 (1.2)*	*4 (0.6)*	*2 (1.8)*	*24 (6.5)*	*15 (5.0)*	*4 (14.8)*	*4 (12.9)*
Poor sleep maintenance [m] ǂ																				
Yes	2,228 (40.1)	1,808 (36.6)	339 (68.9)	33 (51.6)	3,437 (38.3)	2,781 (34.9)	501 (63.3)	70 (64.8)	4,336 (39.1)	3,477 (35.6)	661 (64.3)	85 (57.8)	3,141 (41.7)	2,565 (38.6)	447 (65.8)	59 (52.7)	118 (31.9)	81 (26.9)	16 (59.3)	15 (48.4)
No	3,259 (58.7)	3,066 (62.1)	151 (30.7)	30 (46.9)	5,454 (60.7)	5,102 (64.0)	284 (35.9)	37 (34.3)	6,619 (59.6)	6,162 (63.1)	359 (34.9)	62 (42.2)	4,315 (57.2)	4,007 (60.3)	228 (33.6)	52 (46.4)	230 (62.2)	203 (67.4)	10 (37.0)	13 (41.9)
Missing	*64 (1.2)*	*61 (1.2)*	*2 (0.4)*	*1 (1.6)*	*94 (1.1)*	*84 (1.1)*	*7 (0.9)*	*1 (0.9)*	*143 (1.3)*	*134 (1.4)*	*8 (0.8)*	*0 (0.0)*	*85 (1.1)*	*79 (1.2)*	*4 (0.6)*	*1 (0.9)*	*22 (6.0)*	*17 (5.7)*	*1 (3.7)*	*3 (9.7)*
Insomnia symptoms [n] ǂ																				
Yes	2,380 (42.9)	1,939 (39.3)	356 (72.4)	34 (53.1)	3,739 (41.6)	3,035 (38.1)	536 (67.7)	74 (68.5)	4,742 (42.7)	3,814 (39.0)	712 (69.3)	92 (62.6)	3,357 (44.5)	2,737 (41.2)	480 (70.7)	62 (55.4)	137 (37.0)	96 (31.9)	18 (66.7)	17 (54.8)
No	3,133 (56.4)	2,960 (60.0)	135 (27.4)	29 (45.3)	5,195 (57.8)	4,884 (61.3)	254 (32.1)	34 (31.5)	6,275 (56.5)	5,883 (60.2)	312 (30.4)	55 (37.4)	4,136 (54.9)	3,868 (58.2)	198 (29.2)	49 (43.8)	215 (58.1)	192 (63.8)	8 (29.6)	11 (35.5)
Missing	*38 (0.7)*	*36 (0.7)*	*1 (0.2)*	*1 (1.6)*	*51 (0.6)*	*48 (0.6)*	*2 (0.3)*	*0 (0.0)*	*81 (0.7)*	*76 (0.8)*	*4 (0.4)*	*0 (0.0)*	*48 (0.6)*	*46 (0.7)*	*1 (0.2)*	*1 (0.9)*	*18 (4.9)*	*13 (4.3)*	*1 (3.7)*	*3 (9.7)*
Sleep medication use [o] ǂ																				
Yes	914 (16.5)	782 (15.9)	101 (20.5)	19 (29.7)	1,617 (18.0)	1,392 (17.5)	160 (20.2)	31 (28.7)	2,163 (19.5)	1,839 (18.8)	242 (23.5)	35 (23.8)	1,519 (20.1)	1,317 (19.8)	151 (22.2)	24 (21.4)	77 (20.8)	54 (17.9)	7 (25.9)	12 (38.7)
No	4,611 (83.1)	4,132 (83.7)	387 (78.7)	45 (70.3)	7,320 (81.5)	6,531 (82.0)	628 (79.3)	77 (71.3)	8,888 (80.1)	7,895 (80.8)	781 (76.0)	110 (74.8)	5,991 (79.5)	5,306 (79.8)	527 (77.6)	87 (77.7)	283 (76.5)	240 (79.7)	20 (74.1)	18 (58.1)
Missing	26 (0.5)	21 (0.4)	4 (0.8)	0 (0.0)	48 (0.5)	44 (0.6)	4 (0.5)	0 (0.0)	47 (0.4)	39 (0.4)	5 (0.5)	2 (1.4)	31 (0.4)	28 (0.4)	1 (0.2)	1 (0.9)	10 (2.7)	7 (2.3)	0 (0.0)	1 (3.2)
Daytime dysfunction [p]																				

Yes	36 (0.7)	29 (0.6)	5 (1.0)	2 (3.1)	63 (0.7)	53 (0.7)	7 (0.9)	1 (0.9)	101 (0.9)	68 (0.7)	17 (1.7)	8 (5.4)	50 (0.7)	36 (0.5)	8 (1.2)	4 (3.6)	2 (0.5)	0 (0.0)	1 (3.7)	1 (3.2)
No	5,452 (98.2)	4,847 (98.2)	483 (98.2)	62 (96.9)	8,811 (98.1)	7,816 (98.1)	774 (97.7)	106 (98.2)	10,867 (97.9)	9,589 (98.1)	999 (97.2)	138 (93.9)	7,400 (98.1)	6,531 (98.2)	668 (98.4)	106 (94.6)	356 (96.2)	291 (96.7)	25 (92.6)	30 (96.8)
Missing	*63 (1.1)*	*59 (1.2)*	*4 (0.8)*	*0 (0.0)*	*111 (1.2)*	*98 (1.2)*	*11 (1.4)*	*1 (0.9)*	*130 (1.2)*	*116 (1.2)*	*12 (1.2)*	*1 (0.7)*	*91 (1.2)*	*84 (1.3)*	*3 (0.4)*	*2 (1.8)*	*12 (3.2)*	*10 (3.3)*	*1 (3.7)*	*0 (0.0)*
Healthcare provider diagnosed sleep apnea [q ƚ]																				
Yes	470 (8.5)	403 (8.2)	54 (11.0)	9 (14.1)	936 (10.4)	820 (10.3)	89 (11.2)	10 (9.3)	1,177 (10.6)	981 (10.0)	144 (14.0)	31 (21.1)	852 (11.3)	741 (11.1)	78 (11.5)	20 (17.9)	18 (4.9)	9 (3.0)	3 (11.1)	6 (19.4)
No	5,024 (90.5)	4,481 (90.8)	433 (88.0)	54 (84.4)	7,944 (88.4)	7,058 (88.6)	696 (87.9)	92 (85.2)	9,804 (88.3)	8,684 (88.9)	880 (85.6)	112 (76.2)	6,615 (87.7)	5,845 (87.9)	595 (87.6)	91 (81.3)	344 (93.0)	287 (95.4)	24 (88.9)	24 (77.4)
Missing	*57 (1.0)*	*51 (1.0)*	*5 (1.0)*	*1 (1.6)*	*105 (1.2)*	*89 (1.1)*	*7 (0.9)*	*6 (5.6)*	*117 (1.1)*	*108 (1.1)*	*4 (0.4)*	*4 (2.7)*	*74 (1.0)*	*65 (1.0)*	*6 (0.9)*	*1 (0.9)*	*8 (2.2)*	*5 (1.7)*	*0 (0.0)*	*1 (3.2)*

Data presented as mean ± standard deviation or n (%)
Note: N=438 (1.3%) participants who reported both 'too hot' and 'too cold' temperatures were not included in the analysis.
Abbreviations: SD, Standard Deviation; GED, General Educational Development
[¥]Chi-square test p-values indicate significant differences between in the overall prevalence of 'too hot' by region of residence.
[§]Chi-square test p-values indicate significant differences between the overall prevalence of 'too cold' by region or residence.
[ƚ]Chi-square or ANOVA test p-values indicate significant differences in characteristics between the total populations of each region of residence.

[a] Excluded for reporting both 'Too hot' and 'Too cold', Northeast: n = 60 (1.1%)
[b] Excluded for reporting both 'Too hot' and 'Too cold', Midwest: n = 118 (1.3%)
[c] Excluded for reporting both 'Too hot' and 'Too cold', South: n = 150 (1.4%)
[d] Excluded for reporting both 'Too hot' and 'Too cold', West: n = 99 (1.3%)
[e] Excluded for reporting both 'Too hot' and 'Too cold', Puerto Rico: n = 11 (3.0%)
[f] 'Infrequent temperature extremes' is defined as self-reported trouble sleeping due to feeling too hot < 3 times per week and trouble sleeping due to feeling too cold < 3 times per week in the past month.
[g] 'Too hot' is defined as self-reported trouble sleeping due to feeling too hot ≥ 3 times per week in the past month.
[h] 'Too cold' is defined as self-reported trouble sleeping due to feeling too cold ≥ 3 times per week in the past month.
[i] Educational attainment was assessed at baseline.
[j] Reasons for trouble sleeping ≥ 3 times per week in the past month include: unable to fall asleep within 30 minutes, waking up in the middle of the night or early morning, waking up to use the bathroom, cannot breathe comfortably, coughing or snoring loudly, having bad dreams, having pain, or other non-specified reasons.
[k] Sleep duration is based on reported bed and wake times or reported average sleep duration. Participants who reported ≤ 2 hours or ≥ 23 hours of sleep are excluded. Short: <7 hours; Recommended: 7-9 hours; Long >9 hours
[l] Long sleep onset latency is defined as not falling asleep within 30 minutes at least three times a week during the past month.
[m] Poor sleep maintenance is defined as waking up in the middle of the night or early morning at least three times a week during the past month.
[n] Insomnia symptoms is defined as having long sleep onset latency or poor sleep maintenance.
[o] Sleep medication use is defined as 'taking medicine (prescription or over the counter) to help you sleep' at least three times a week during the past month.
[p] Daytime dysfunction is defined as having trouble staying awake while driving, eating, or engaging in social activity at least three times a week during the past month.
[q] Healthcare provider diagnosed sleep apnea is defined as a current doctor or other health professional diagnosis of sleep apnea.

Supplemental Table 5. Sociodemographic characteristics, clinical characteristics, and sleep health dimensions among eligible Sister Study participants by menopausal status at 4th follow-up as well as extreme indoor temperature, Sister Study, 2017 – 2019, N = 33,545

**Menopausal status**	**Premenopausal [a]**				**Postmenopausal [b]**			
	Total n = 1,346 (4.0%)	Infrequent temperature extremes [c] n = 1,110 (82.5%)	Too hot [d ¥] n = 183 (13.6%)	Too cold [e §] n = 25 (1.9%)	Total n = 32,193 (96.0%)	Infrequent temperature extremes [c] n = 28,511 (88.6%)	Too hot [d ¥] n = 2,835 (8.8%)	Too cold [e §] n = 437 (1.4%)
**Sociodemographic characteristics**								
Age, years (mean ± SD) [ǂ]	51.2 ± 2.6	51.1 ± 2.6	51.7 ± 2.6	50.9 ± 2.7	67.8 ± 8.0	68.3 ± 8.0	63.3 ± 7.3	69.5 ± 8.3
Age [ǂ]								
< 67.2 yrs	1,346 (100.0)	1,110 (100.0)	183 (100.0)	25 (100.0)	15,432 (47.9)	12,977 (45.5)	1,997 (70.4)	166 (38.0)
≥ 67.2 yrs	0 (0.0)	0 (0.0)	0 (0.0)	0 (0.0)	16,761 (52.1)	15,534 (54.5)	838 (29.6)	271 (62.0)
Race and ethnicity [ǂ]								
Hispanic/Latina	87 (6.5)	79 (7.1)	7 (3.8)	1 (4.0)	1,138 (3.5)	963 (3.4)	105 (3.7)	45 (10.3)
Non-Hispanic Black	143 (10.6)	104 (9.4)	29 (15.9)	5 (20.0)	2,055 (6.4)	1,696 (6.0)	295 (10.4)	29 (6.6)
Non-Hispanic White	1,116 (82.9)	927 (83.5)	147 (80.3)	19 (76.0)	29,000 (90.1)	25,852 (90.7)	2,435 (85.9)	363 (83.1)
Educational attainment [f ǂ]								
<High school / GED	112 (8.3)	87 (7.8)	18 (9.8)	5 (20.0)	4,349 (13.5)	3,792 (13.3)	410 (14.5)	88 (20.1)
Some college / technical	352 (26.2)	276 (24.9)	55 (30.1)	8 (32.0)	10,236 (31.8)	8,968 (31.5)	963 (34.0)	160 (36.6)
≥ Bachelor's or higher	882 (65.5)	747 (67.3)	110 (60.1)	12 (48.0)	17,604 (54.7)	15,748 (55.2)	1,462 (51.6)	189 (43.3)
Missing	*0 (0.0)*	*0 (0.0)*	*0 (0.0)*	*0 (0.0)*	*4 (0.0)*	*3 (0.0)*	*0 (0.0)*	*0 (0.0)*
Annual household income [ǂ]								
< $20,000 - $49,999	144 (10.7)	116 (10.5)	22 (12.0)	1 (4.0)	8,751 (27.2)	7,828 (27.5)	617 (21.8)	195 (44.6)
$50,000 – $99,999	345 (25.6)	272 (24.5)	53 (29.0)	9 (36.0)	12,031 (37.4)	10,737 (37.7)	1,018 (35.9)	125 (28.6)
≥ $100,000	857 (63.7)	722 (65.1)	108 (59.0)	15 (60.0)	11,411 (35.5)	9,946 (34.9)	1,200 (42.3)	117 (26.8)
Marital status [ǂ]								
Married or living as though married	1,025(76.2)	852 (76.8)	137 (74.9)	21 (84.0)	21,775 (67.6)	19,014 (66.7)	2,188(77.2)	275 (62.9)
Never married, divorced, widowed, or separated	321 (23.8)	258 (23.2)	46 (25.1)	4 (16.0)	10,418 (32.4)	9,497 (33.3)	647 (22.8)	162 (37.1)
Region of residence								
Northeast	230 (17.1)	192 (17.3)	31 (16.9)	2 (8.0)	5,321 (16.5)	4,743 (16.6)	461 (16.3)	62 (14.2)
Midwest	376 (27.9)	314 (28.3)	50 (27.3)	7 (28.0)	8,608 (26.7)	7,652 (26.8)	742 (26.2)	101 (23.1)
South	437 (32.5)	350 (31.5)	65 (35.5)	10 (40.0)	10,657 (33.1)	9,419 (33.0)	963 (34.0)	137 (31.4)
West	282 (21.0)	235 (21.2)	36 (19.7)	5 (20.0)	7,258 (22.6)	6,415 (22.5)	643 (22.7)	107 (24.5)
Puerto Rico	21 (1.6)	19 (1.7)	1 (0.6)	1 (4.0)	349 (1.1)	282 (1.0)	26 (0.9)	30 (6.9)
**Clinical characteristics**								
Body Mass Index, kg/m^2 [ǂ]								
18.5 - 24.9	496 (36.9)	426 (38.4)	47 (25.7)	10 (40.0)	12,630 (39.2)	11,358 (39.8)	941 (33.2)	180 (41.2)
25.0 - 29.9	390 (29.0)	320 (28.8)	57 (31.2)	5 (20.0)	10,375 (32.2)	9,172 (32.2)	956 (33.7)	118 (27.0)
≥30	446 (33.1)	354 (31.9)	75 (41.0)	10 (40.0)	8,772 (27.3)	7,612 (26.7)	906 (32.0)	133 (30.4)
Missing	*14 (1.0)*	*10 (0.9)*	*4 (2.2)*	*0 (0.0)*	*416 (1.3)*	*369 (1.3)*	*32 (1.1)*	*6 (1.4)*

Trouble sleeping for any other reason [g ǂ]								
Yes	791 (58.8)	588 (53)	158 (86.3)	20 (80.0)	23,057 (71.6)	19,754 (69.3)	2,524 (89.0)	386 (88.3)
No	555 (41.2)	522 (47)	25 (13.7)	5 (20.0)	9,135 (28.4)	8,756 (30.7)	311 (11.0)	51 (11.7)
Missing	*0 (0.0)*	*0 (0.0)*	*0 (0.0)*	*0 (0.0)*	*1 (0.0)*	*1 (0.0)*	*0 (0.0)*	*0 (0.0)*
**Sleep health characteristics**								
Average weekly sleep duration [h ǂ]								
Short	183 (13.6)	146 (13.2)	27 (14.8)	6 (24.0)	3,640 (11.3)	3,136 (11.0)	374 (13.2)	70 (16.0)
Recommended	1,023 (76)	858 (77.3)	130 (71)	16 (64.0)	23,367 (72.6)	20,895 (73.3)	1,952 (68.9)	262 (60.0)
Long	134 (10.0)	102 (9.2)	25 (13.7)	3 (12.0)	4,788 (14.9)	4,131 (14.5)	483 (17.0)	88 (20.1)
Missing	*6 (0.5)*	*4 (0.4)*	*1 (0.6)*	*0 (0.0)*	*398 (1.2)*	*349 (1.2)*	*26 (0.9)*	*17 (3.9)*
Long sleep onset latency [i ǂ]								
Yes	125 (9.3)	76 (6.9)	36 (19.7)	2 (8.0)	4,057 (12.6)	3,120 (10.9)	653 (23.0)	118 (27.0)
No	1,207 (89.7)	1,024 (92.3)	144 (78.7)	23 (92.0)	27,740 (86.2)	25,036 (87.8)	2,158 (76.1)	308 (70.5)
Missing	*14 (1.0)*	*10 (0.9)*	*3 (1.6)*	*0 (0.0)*	*396 (1.2)*	*355 (1.3)*	*24 (0.9)*	*11 (2.5)*
Poor sleep maintenance [j ǂ]								
Yes	478 (35.5)	330 (29.7)	117 (63.9)	14 (56.0)	12,779 (39.7)	10,379 (36.4)	1,847 (65.2)	248 (56.8)
No	856 (63.6)	773 (69.6)	61 (33.3)	11 (44.0)	19,018 (59.1)	17,764 (62.3)	971 (34.3)	183 (41.9)
Missing	*12 (0.9)*	*7 (0.6)*	*5 (2.7)*	*0 (0.0)*	*396 (1.2)*	*368 (1.3)*	*17 (0.6)*	*6 (1.4)*
Insomnia symptoms [k ǂ]								
Yes	512 (38.0)	354 (31.9)	125 (68.3)	14 (56.0)	13,840 (43.0)	11,264 (39.5)	1,977 (69.7)	265 (60.6)
No	827 (61.4)	750 (67.6)	57 (31.2)	11 (44.0)	18,124 (56.3)	17,034 (59.8)	850 (30.0)	167 (38.2)
Missing	*7 (0.5)*	*6 (0.5)*	*1 (0.6)*	*0 (0.0)*	*229 (0.7)*	*213 (0.8)*	*8 (0.3)*	*5 (1.1)*
Sleep medication use [l ǂ]								
Yes	181 (13.5)	139 (12.5)	30 (16.4)	6 (24.0)	6,109 (19.0)	5,245 (18.4)	631 (22.3)	115 (26.3)
No	1,159 (86.1)	965 (86.9)	153 (83.6)	19 (76.0)	25,928 (80.5)	23,133 (81.1)	2,190 (77.3)	318 (72.8)
Missing	*6 (0.5)*	*6 (0.5)*	*0 (0.0)*	*0 (0.0)*	*156 (0.5)*	*133 (0.5)*	*14 (0.5)*	*4 (0.9)*
Daytime dysfunction [m]								
Yes	8 (0.6)	5 (0.5)	1 (0.6)	2 (8.0)	243 (0.8)	180 (0.6)	37 (1.3)	14 (3.2)
No	1,326 (98.5)	1,095 (98.7)	180 (98.4)	23 (92.0)	31,555 (98.0)	27,974 (98.1)	2,769 (97.7)	419 (95.9)
Missing	*12 (0.9)*	*10 (0.9)*	*2 (1.1)*	*0 (0.0)*	*395 (1.2)*	*357 (1.3)*	*29 (1.0)*	*4 (0.9)*
Healthcare provider diagnosed sleep apnea [n ǂ]								
Yes	93 (6.9)	73 (6.6)	14 (7.7)	2 (8.0)	3,359 (10.4)	2,880 (10.1)	354 (12.5)	74 (16.9)
No	1,247 (92.6)	1,031 (92.9)	169 (92.4)	23 (92.0)	28,479 (88.5)	25,319 (88.8)	2,459 (86.7)	350 (80.1)
Missing	*6 (0.5)*	*6 (0.5)*	*0 (0.0)*	*0 (0.0)*	*355 (1.1)*	*312 (1.1)*	*22 (0.8)*	*13 (3.0)*

Data presented as mean ± standard deviation or n (%)

Note: N=438 (1.3%) participants who reported both ‘too hot’ and ‘too cold’ temperatures were not included in the analysis.

Abbreviations: SD, Standard Deviation; GED, General Educational Development

[¥]Chi-square test p-values indicate significant differences between the overall prevalence of ‘too hot’ by menopausal status.

[§]Chi-square test p-values indicate significant differences between the overall prevalence of ‘too cold’ by menopausal status.

[ǂ]Chi-square or ANOVA test p-values indicate significant differences in characteristics between the total populations of each menopausal status category.

[a] Excluded for reporting both ‘Too hot’ and ‘Too cold’, Premenopausal: n = 28 (2.1%)
[b] Excluded for reporting both ‘Too hot’ and ‘Too cold’, Postmenopausal: n = 410 (1.3%)
[c] ‘Infrequent temperature extremes’ is defined as self-reported trouble sleeping due to feeling too hot < 3 times per week and trouble sleeping due to feeling too cold < 3 times per week in the past month.
[d] ’Too hot’ is defined as self-reported trouble sleeping due to feeling too hot ≥ 3 times per week in the past month.
[e] ’Too cold’ is defined as self-reported trouble sleeping due to feeling too cold ≥ 3 times per week in the past month.
[f] Educational attainment was assessed at baseline.
[g] Reasons for trouble sleeping ≥ 3 times per week in the past month include: unable to fall asleep within 30 minutes, waking up in the middle of the night or early morning, waking up to use the bathroom, cannot breathe comfortably, coughing or snoring loudly, having bad dreams, having pain, or other non-specified reasons.
[h] Sleep duration is based on reported bed and wake times or reported average sleep duration. Participants who reported ≤ 2 hours or ≥ 23 hours of sleep are excluded. Short: <7 hours; Recommended: 7-9 hours; Long >9 hours
[i] Long sleep onset latency is defined as not falling asleep within 30 minutes at least three times a week during the past month.
[j] Poor sleep maintenance is defined as waking up in the middle of the night or early morning at least three times a week during the past month.
[k] Insomnia symptoms is defined as long sleep onset latency or poor sleep maintenance.
[l] Sleep medication use is defined as 'taking medicine (prescription or over the counter) to help you sleep' at least three times a week during the past month.
[m] Daytime dysfunction is defined as having trouble staying awake while driving, eating, or engaging in social activity at least three times a week during the past month.
[n] Healthcare provider diagnosed sleep apnea is defined as a current doctor or other health professional diagnosis of sleep apnea

Supplemental Table 6. Prevalence ratios (95% confidence intervals) for associations between perceived indoor temperature extremes and sleep health dimensions, Sister Study, 2017-2019, N=33,545

	Indoor temperature: Too hot [a]				Indoor temperature: Too cold [b]			
	Crude	Model 1 [c]	Model 2	Model 3	Crude	Model 1 [c]	Model 2	Model 3
Weekly sleep duration [d]								
Short	**1.23 (1.12,1.36)**[ǂ]	**1.19 (1.08,1.31)**[‡]	1.09 (0.99,1.21)	1.09 (0.99,1.20)	**1.66 (1.35,2.03)**[‡]	**1.60 (1.31,1.96)**[‡]	**1.46 (1.20,1.78)**[‡]	**1.46 (1.20,1.78)**[‡]
Recommended	1.00 (Ref)	1.00 (Ref)	1.00 (Ref)	1.00 (Ref)	1.00 (Ref)	1.00 (Ref)	1.00 (Ref)	1.00 (Ref)
Long	**1.20 (1.10,1.30)**[‡]	**1.13 (1.03,1.22)**[‡]	**1.26 (1.15,1.37)**[‡]	**1.25 (1.15,1.36)**[‡]	**1.51 (1.25,1.81)**[‡]	**1.42 (1.18,1.70)**[‡]	**1.35 (1.12,1.62)**[‡]	**1.35 (1.13,1.62)**[‡]
Long sleep onset latency [e]								
Yes	**2.11 (1.96,2.27)**[‡]	**1.89 (1.76,2.04)**[‡]	**1.91 (1.77,2.06)**[‡]	**1.88 (1.74,2.03)**[‡]	**2.44 (2.09,2.86)**[‡]	**2.14 (1.82,2.50)**[‡]	**1.99 (1.70,2.33)**[‡]	**1.99 (1.70,2.33)**[‡]
No	1.00 (Ref)	1.00 (Ref)	1.00 (Ref)	1.00 (Ref)	1.00 (Ref)	1.00 (Ref)	1.00 (Ref)	1.00 (Ref)
Poor sleep maintenance [f]								
Yes	**1.79 (1.74,1.85)**[‡]	**1.58 (1.53,1.63)**[‡]	**1.57 (1.52,1.62)**[‡]	**1.57 (1.52,1.62)**[‡]	**1.56 (1.44,1.69)**[‡]	**1.33 (1.22,1.44)**[‡]	**1.34 (1.24,1.45)**[‡]	**1.34 (1.23,1.45)**[‡]
No	1.00 (Ref)	1.00 (Ref)	1.00 (Ref)	1.00 (Ref)	1.00 (Ref)	1.00 (Ref)	1.00 (Ref)	1.00 (Ref)
Insomnia symptoms [g]								
Yes	**1.77 (1.72,1.82)**[‡]	**1.57 (1.53,1.62)**[‡]	**1.57 (1.52,1.61)**[‡]	**1.56 (1.52,1.61)**[‡]	**1.54 (1.43,1.66)**[‡]	**1.32 (1.23,1.43)**[‡]	**1.33 (1.24,1.44)**[‡]	**1.33 (1.23,1.43**[)‡]
No	1.00 (Ref)	1.00 (Ref)	1.00 (Ref)	1.00 (Ref)	1.00 (Ref)	1.00 (Ref)	1.00 (Ref)	1.00 (Ref)
Sleep medication use [h]								
Yes	**1.20 (1.12,1.29)**[‡]	**1.10 (1.02,1.18)**[‡]	**1.18 (1.10,1.27)**[‡]	**1.18 (1.09,1.27)**[‡]	**1.44 (1.23,1.68)**[‡]	**1.33 (1.13,1.55)**[‡]	**1.31 (1.12,1.53)**[‡]	**1.31 (1.12,1.53)**[‡]
No	1.00 (Ref)	1.00 (Ref)	1.00 (Ref)	1.00 (Ref)	1.00 (Ref)	1.00 (Ref)	1.00 (Ref)	1.00 (Ref)
Daytime dysfunction [i]								
Yes	**2.03 (1.44,2.88)**[‡]	**1.79 (1.26,2.54)**[‡]	**1.79 (1.25,2.55)‡**	**1.76 (1.23,2.52)**[‡]	**5.59 (3.38,9.23)**[‡]	**4.94 (2.99,8.16)**[‡]	**4.81 (2.91,7.95)**[‡]	**4.69 (2.83,7.77)**[‡]
No	1.00 (Ref)	1.00 (Ref)	1.00 (Ref)	1.00 (Ref)	1.00 (Ref)	1.00 (Ref)	1.00 (Ref)	1.00 (Ref)
Healthcare professional diagnosed sleep apnea [j]								
Yes	**1.22 (1.10,1.35)**[‡]	**1.19 (1.07,1.32)**[‡]	**1.26 (1.14,1.40)**[‡]	**1.20 (1.09,1.33)**[‡]	**1.67 (1.36,2.06)**[‡]	**1.63 (1.32,2.01)**[‡]	**1.64 (1.33,2.01)**[‡]	**1.62 (1.33,1.97)**[‡]
No	1.00 (Ref)	1.00 (Ref)	1.00 (Ref)	1.00 (Ref)	1.00 (Ref)	1.00 (Ref)	1.00 (Ref)	1.00 (Ref)

Note: N=438 (1.3%) participants who reported both 'too hot' and 'too cold' temperatures were not included in the analysis.

Model 1: Adjusted for frequent trouble sleeping for any reason other than feeling too hot or feeling too cold.
Model 2: Adjusted for frequent trouble sleeping for any reason other than feeling too hot or feeling too cold, age (continuous), age^2, race and ethnicity (Hispanic/Latina, non-Hispanic Black, non-Hispanic White), annual household income (<$20,000 - $49,999, $50,000 - $99,999, ≥$100,000), marital status (married or living as though married, divorced/widowed/separated/never married), and region of residence (Northeast, Midwest, South, West, and Puerto Rico).
Model 3: Adjusted for trouble sleeping for any reason other than feeling too hot or feeling too cold, age (continuous), age^2, race and ethnicity (Hispanic/Latina, non-Hispanic Black, non-Hispanic White), annual household income (<$20,000 - $49,999, $50,000 - $99,999, ≥$100,000), marital status (married or living as though married, divorced/widowed/separated/never married), region of residence (Northeast, Midwest, South, West, and Puerto Rico), body mass index (BMI: underweight, recommended, overweight, obesity), and menopausal status (premenopausal, postmenopausal).

[a] Too hot' is defined as self-reported trouble sleeping due to feeling too hot ≥ 3 times per week vs. trouble sleeping due to feeling too hot < 3 times per week and trouble sleeping due to feeling too cold < 3 times per week.
[b] 'Too cold' is defined as self-reported trouble sleeping due to feeling too cold ≥ 3 times per week vs. trouble sleeping due to feeling too hot < 3 times per week and trouble sleeping due to feeling too cold < 3 times per week.

[c] In models for weekly sleep duration, sleep medication use, daytime dysfunction, and healthcare professional diagnosed sleep apnea, reasons for trouble sleeping for any reason other than feeling too hot or feeling too cold ≥ 3 times per week in the past month include: unable to fall asleep within 30 minutes, waking up in the middle of the night or early morning, waking up to use the bathroom, cannot breathe comfortably, coughing or snoring loudly, having bad dreams, having pain, or other non-specified reasons. In models for long sleep onset latency, poor sleep maintenance, and insomnia symptoms, reasons for trouble sleeping for any reason other than feeling too hot or feeling too cold ≥ 3 times per week in the past month include waking up to use the bathroom, cannot breathe comfortably, coughing or snoring loudly, having bad dreams, having pain, or other non-specified reasons.
[d] Sleep duration is based on reported bed and wake times or reported average sleep duration. Participants who reported ≤ 2 hours or ≥ 23 hours of sleep are excluded. Short: <7 hours; Recommended: 7-9 hours; Long >9 hours
[e] Long sleep onset latency is defined as not falling asleep within 30 minutes at least three times a week during the past month.
[f] Poor sleep maintenance is defined as waking up in the middle of the night or early morning at least three times a week during the past month.
[g] Insomnia symptoms is defined as long sleep onset latency or poor sleep maintenance.
[h] Sleep medication use is defined as 'taking medicine (prescription or over the counter) to help you sleep' at least three times a week during the past month.
[i] Daytime dysfunction is defined as having trouble staying awake while driving, eating, or engaging in social activity at least three times a week during the past month.
[j] Healthcare provider diagnosed sleep apnea is defined as a current doctor or other health professional diagnosis of sleep apnea.
ƚ Remained significant after false discovery rate correction. False discovery rate-corrected P-value was considered statistically significant at the 0.05 level.

Supplemental Table 7A. Prevalence ratios (95% confidence intervals) for associations between perceived indoor temperature extremes and sleep health dimensions by race and ethnicity, Sister Study, 2017-2019, N=33,545

	**Indoor temperature: Too hot [a]**				**Indoor temperature: Too cold [b]**			
	Hispanic/Latina	Non-Hispanic Black	Non-Hispanic White	Wald p-values	Hispanic/Latina	Non-Hispanic Black	Non-Hispanic White	Wald p-values
Weekly sleep duration [c]								
Short	1.19 (0.79,1.80)	0.97 (0.81,1.17)	**1.12 (1.00,1.26)‡**	0.3834	0.92 (0.43,1.98)	1.38 (0.96,2.00)	**1.57 (1.25,1.99)‡**	0.3929
Recommended	1.00 (ref)	1.00 (ref)	1.00 (ref)	---	1.00 (ref)	1.00 (ref)	1.00 (ref)	---
Long	**1.47 (1.02,2.13)‡**	0.95 (0.69,1.32)	**1.27 (1.16,1.39)‡**	0.1690	1.15 (0.61,2.14)	1.27 (0.53,3.02)	**1.38 (1.14,1.68)‡**	0.8460
Long sleep onset latency [d]								
Yes	**2.24 (1.70,2.96)‡**	**2.04 (1.65,2.52)‡**	**1.84 (1.69,2.00)‡**	0.3079	**1.70 (1.08,2.67)‡**	1.52 (0.77,2.99)	**2.08 (1.75,2.47)‡**	0.5131
No	1.00 (ref)	1.00 (ref)	1.00 (ref)	---	1.00 (ref)	1.00 (ref)	1.00 (ref)	---
Poor sleep maintenance [e]								
Yes	**1.68 (1.41,1.99)‡**	**1.47 (1.31,1.65)‡**	**1.58 (1.53,1.63)‡**	0.4053	**1.58 (1.18,2.11)‡**	**1.39 (1.05,1.85)‡**	**1.31 (1.20,1.43)‡**	0.4680
No	1.00 (ref)	1.00 (ref)	1.00 (ref)	---	1.00 (ref)	1.00 (ref)	1.00 (ref)	---
Insomnia symptoms [f]								
Yes	**1.75 (1.51,2.02)‡**	**1.49 (1.34,1.65)‡**	**1.57 (1.52,1.61)‡**	0.2065	**1.50 (1.16,1.95)‡**	**1.45 (1.12,1.88)‡**	**1.30 (1.20,1.42)‡**	0.4825
No	1.00 (ref)	1.00 (ref)	1.00 (ref)	---	1.00 (ref)	1.00 (ref)	1.00 (ref)	---
Sleep medication use [g]								
Yes	1.13 (0.72,1.76)	1.27 (0.93,1.72)	**1.17 (1.09,1.27)‡**	0.8807	1.53 (0.94,2.48)	1.08 (0.43,2.73)	**1.30 (1.10,1.54)‡**	0.7548
No	1.00 (ref)	1.00 (ref)	1.00 (ref)	---	1.00 (ref)	1.00 (ref)	1.00 (ref)	---
Daytime dysfunction [h]								
Yes	0.76 (0.09,6.37)	2.32 (0.79,6.82)	**1.77 (1.21,2.60)‡**	0.6548	**7.15 (1.79,28.56)‡**	**12.52 (3.51,44.59)‡**	**3.84 (2.09,7.08)‡**	0.2220
No	1.00 (ref)	1.00 (ref)	1.00 (ref)	---	1.00 (ref)	1.00 (ref)	1.00 (ref)	---
Healthcare professional diagnosed sleep apnea [i]								
Yes	1.52 (0.90,2.58)	1.29 (0.99,1.68)	**1.18 (1.05,1.32)‡**	0.5567	**3.12 (1.68,5.79)‡**	**2.39 (1.40,4.08)‡**	**1.45 (1.15,1.82)‡**	**0.0276**
No	1.00 (ref)	1.00 (ref)	1.00 (ref)	---	1.00 (ref)	1.00 (ref)	1.00 (ref)	---

Note: N=438 (1.3%) participants who reported both ‘too hot’ and ‘too cold’ temperatures were not included in the analysis.

Models are adjusted for frequent trouble sleeping for any reason other than feeling too hot or feeling too cold, age (continuous), age^2, annual household income (<$20,000 - $49,999, $50,000 - $99,999, ≥$100,000), marital status (married or living as though married, divorced/widowed/separated/never married), region of residence (Northeast, Midwest, South, West, and Puerto Rico), body mass index (BMI: underweight, recommended, overweight, obesity), and menopausal status (premenopausal, postmenopausal). In models for weekly sleep duration, sleep medication use, daytime dysfunction, and healthcare professional diagnosed sleep apnea, reasons for trouble sleeping for any reason other than feeling too hot or feeling too cold ≥ 3 times per week in the past month include: unable to fall asleep within 30 minutes, waking up in the middle of the night or early morning, waking up to use the bathroom, cannot breathe comfortably, coughing or snoring loudly, having bad dreams, having pain, or other non-specified reasons. In models for long sleep onset latency, poor sleep maintenance, and insomnia symptoms, reasons for trouble sleeping for any reason other than feeling too hot or feeling too cold ≥ 3 times per week in the past month include waking up to use the bathroom, cannot breathe comfortably, coughing or snoring loudly, having bad dreams, having pain, or other non-specified reasons.

[a] 'Too hot' is defined as self-reported trouble sleeping due to feeling too hot ≥ 3 times per week vs. trouble sleeping due to feeling too hot < 3 times per week and trouble sleeping due to feeling too cold < 3 times per week.
[b] 'Too cold' is defined as self-reported trouble sleeping due to feeling too cold ≥ 3 times per week vs. trouble sleeping due to feeling too hot < 3 times per week and trouble sleeping due to feeling too cold < 3 times per week.
[c] Sleep duration is based on reported bed and wake times or reported average sleep duration. Participants who reported ≤ 2 hours or ≥ 23 hours of sleep are excluded. Short: <7 hours; Recommended: 7-9 hours; Long >9 hours
[d] Long sleep onset latency is defined as not falling asleep within 30 minutes at least three times a week during the past month.
[e] Poor sleep maintenance is defined as waking up in the middle of the night or early morning at least three times a week during the past month.
[f] Insomnia is defined as long sleep onset latency or poor sleep maintenance.
[g] Sleep medication use is defined as 'taking medicine (prescription or over the counter) to help you sleep' at least three times a week during the past month.
[h] Daytime dysfunction is defined as having trouble staying awake while driving, eating, or engaging in social activity at least three times a week during the past month.
[i] Healthcare provider diagnosed sleep apnea is defined as a current doctor or other health professional diagnosis of sleep apnea.
[ł] Remained significant after false discovery rate correction. False discovery rate-corrected P-value was considered statistically significant at the 0.05 level.

Supplemental Table 7B. Relative excess risk due to interaction (RERI) between perceived indoor temperature extremes and race and ethnicity, Sister Study, 2017-2019, N=33,545

	**Indoor temperature: Too hot [a]**			**Indoor temperature: Too cold [b]**		
	Hispanic/Latina	Non-Hispanic Black	Non-Hispanic White	Hispanic/Latina	Non-Hispanic Black	Non-Hispanic White
Weekly sleep duration [c]						
Short	0.17 (-0.55, 0.89)	-0.19 (-0.65, 0.27)		-0.70 (-1.83, 0.43)	0.37 (-0.93, 1.67)	
Recommended	---	---	1.00 (Ref)	---	---	1.00 (Ref)
Long	0.30 (-0.32, 0.92)	-0.31 (-0.64, 0.02)		-0.20 (-1.11, 0.71)	-0.11 (-1.24, 1.02)	
Long sleep onset latency [d]						
Yes	**0.79 (0.06, 1.52)**	0.19 (-0.20, 0.58)	1.00 (Ref)	-0.20 (-1.20, 0.80)	-0.56 (-1.65, 0.53)	1.00 (Ref)
No	---	---		---	---	
Poor sleep maintenance [e]						
Yes	0.02 (-0.21, 0.25)	**-0.15 (-0.29, -0.01)**	1.00 (Ref)	0.20 (-0.21, 0.61)	0.04 (-0.33, 0.41)	1.00 (Ref)
No	---	---		---	---	
Insomnia symptoms [f]						
Yes	0.12 (-0.07, 0.31)	**-0.13 (-0.26, -0.00)**	1.00 (Ref)	0.15 (-0.21, 0.51)	0.10 (-0.25, 0.45)	1.00 (Ref)
No	---	---		---	---	
Sleep medication use [g]						
Yes	-0.09 (-0.41, 0.23)	-0.03 (-0.25, 0.19)	1.00 (Ref)	0.02 (-0.47, 0.51)	-0.26 (-0.84, 0.32)	1.00 (Ref)
No	---	---		---	---	
Daytime dysfunction [h]						
Yes	-1.19 (-4.02, 1.64)	0.24 (-1.52, 2.00)	1.00 (Ref)	8.61 (-8.14, 25.36)	6.21 (-5.18, 17.60)	1.00 (Ref)
No	---	---		---	---	
Healthcare professional diagnosed sleep apnea [i]						
Yes	0.22 (-0.36, 0.80)	0.08 (-0.24, 0.40)	1.00 (Ref)	1.14 (-0.27, 2.55)	0.81 (-0.36, 1.98)	1.00 (Ref)
No	---	---		---	---	

Relative Excess Risk due to Interaction (RERI) = 0: no additive interaction; RERI >0: positive additive interaction; RERI <0: negative additive interaction

Models are adjusted for frequent trouble sleeping for any reason other than feeling too hot or feeling too cold, age (continuous), age^2, annual household income (<$20,000 - $49,999, $50,000 - $99,999, ≥$100,000), marital status (married or living as though married, divorced/widowed/separated/never married), region of residence (Northeast, Midwest, South, West, and Puerto Rico), body mass index (BMI: underweight, recommended, overweight, obesity), and menopausal status (premenopausal, postmenopausal). In models for weekly sleep duration, sleep medication use, daytime dysfunction, and healthcare professional diagnosed sleep apnea, reasons for trouble sleeping for any reason other than feeling too hot or feeling too cold ≥ 3 times per week in the past month include: unable to fall asleep within 30 minutes, waking up in the middle of the night or early morning, waking up to use the bathroom, cannot breathe comfortably, coughing or snoring loudly, having bad dreams, having pain, or other non-specified reasons. In models for long sleep onset latency, poor sleep maintenance, and insomnia symptoms, reasons for trouble sleeping for any reason other than feeling too hot or feeling too cold ≥ 3 times per week in the past month include waking up to use the bathroom, cannot breathe comfortably, coughing or snoring loudly, having bad dreams, having pain, or other non-specified reasons.

[a] 'Too hot' is defined as self-reported trouble sleeping due to feeling too hot ≥ 3 times per week vs. trouble sleeping due to feeling too hot < 3 times per week and trouble sleeping due to feeling too cold < 3 times per week.
[b] 'Too cold' is defined as self-reported trouble sleeping due to feeling too cold ≥ 3 times per week vs. trouble sleeping due to feeling too hot < 3 times per week and trouble sleeping due to feeling too cold < 3 times per week.

[c] Sleep duration is based on reported bed and wake times or reported average sleep duration. Participants who reported ≤ 2 hours or ≥ 23 hours of sleep are excluded. Short: <7 hours; Recommended: 7-9 hours; Long >9 hours
[d] Long sleep onset latency is defined as not falling asleep within 30 minutes at least three times a week during the past month.
[e] Poor sleep maintenance is defined as waking up in the middle of the night or early morning at least three times a week during the past month.
[f] Insomnia is defined as long sleep onset latency or poor sleep maintenance.
[g] Sleep medication use is defined as 'taking medicine (prescription or over the counter) to help you sleep' at least three times a week during the past month.
[h] Daytime dysfunction is defined as having trouble staying awake while driving, eating, or engaging in social activity at least three times a week during the past month.
[i] Healthcare provider diagnosed sleep apnea is defined as a current doctor or other health professional diagnosis of sleep apnea.

Supplemental Table 8A. Prevalence ratios (95% confidence intervals) for associations between perceived indoor temperature extremes and sleep health dimensions by annual household income, Sister Study, 2017-2019, N = 33,545

	Indoor temperature: Too hot [a]				Indoor temperature: Too cold [b]			
	<$20,000 - $49,999	$50,000 - $99,999	≥ $100,000	Wald p-values	<$20,000 - $49,999	$50,000 - $99,999	≥ $100,000	Wald p-values
Weekly sleep duration [c]								
Short	1.02 (0.84,1.24)	**1.20 (1.03,1.40)**‡	1.02 (0.87,1.20)	0.2546	**1.47 (1.10,1.96)**‡	**1.49 (1.03,2.16)**‡	1.41 (0.95,2.09)	0.9772
Recommended	1.00 (Ref)	1.00 (Ref)	1.00 (Ref)	---	1.00 (Ref)	1.00 (Ref)	1.00 (Ref)	---
Long	**1.22 (1.05,1.43)**‡	**1.17 (1.01,1.35)**‡	**1.35 (1.17,1.54)**‡	0.3616	**1.36 (1.05,1.76)**‡	1.02 (0.68,1.53)	**1.73 (1.25,2.40)**‡	0.1325
Long sleep onset latency [d]								
Yes	**1.89 (1.65,2.15)**‡	**2.02 (1.80,2.28)**‡	**1.73 (1.51,1.98)**‡	0.2201	**2.00 (1.62,2.48)**‡	**1.91 (1.40,2.60)**‡	**2.06 (1.44,2.94)**‡	0.9480
No	1.00 (Ref)	1.00 (Ref)	1.00 (Ref)	---	1.00 (Ref)	1.00 (Ref)	1.00 (Ref)	---
Poor sleep maintenance [e]								
Yes	**1.54 (1.45,1.64)**‡	**1.54 (1.46,1.62)**‡	**1.62 (1.54,1.70)**‡	0.2592	**1.33 (1.18,1.50)**‡	**1.46 (1.26,1.68)**‡	**1.22 (1.04,1.44)**‡	0.2815
No	1.00 (Ref)	1.00 (Ref)	1.00 (Ref)	---	1.00 (Ref)	1.00 (Ref)	1.00 (Ref)	---
Insomnia symptoms [f]								
Yes	**1.53 (1.45,1.62)**‡	**1.53 (1.46,1.60)**‡	**1.61 (1.54,1.68)**‡	0.2131	**1.32 (1.18,1.48)**‡	**1.41 (1.23,1.61)**‡	**1.27 (1.09,1.47)**‡	0.5785
No	1.00 (Ref)	1.00 (Ref)	1.00 (Ref)	---	1.00 (Ref)	1.00 (Ref)	1.00 (Ref)	---
Sleep medication use [g]								
Yes	**1.29 (1.12,1.48)**‡	**1.19 (1.05,1.34)**‡	1.10 (0.98,1.24)	0.2363	**1.36 (1.08,1.70)**‡	1.00 (0.72,1.40)	**1.59 (1.22,2.09)**‡	0.1085
No	1.00 (Ref)	1.00 (Ref)	1.00 (Ref)	---	1.00 (Ref)	1.00 (Ref)	1.00 (Ref)	---
Daytime dysfunction [h]								
Yes	1.68 (0.89,3.16)	**2.01 (1.12,3.60)**‡	1.61 (0.87,2.97)	0.8570	**2.76 (1.14,6.66)**‡	**7.96 (3.72,17.03)**‡	**5.24 (1.96,14.04)**‡	0.2042
No	1.00 (Ref)	1.00 (Ref)	1.00 (Ref)	---	1.00 (Ref)	1.00 (Ref)	1.00 (Ref)	---
Healthcare professional diagnosed sleep apnea [i]								
Yes	**1.25 (1.04,1.50)**‡	**1.20 (1.02,1.41)**‡	1.16 (0.97,1.38)	0.8342	**1.58 (1.19,2.09)**‡	**1.70 (1.17,2.45)**‡	**1.62 (1.06,2.47)**‡	0.9554
No	1.00 (Ref)	1.00 (Ref)	1.00 (Ref)	---	1.00 (Ref)	1.00 (Ref)	1.00 (Ref)	---

Note: N=438 (1.3%) participants who reported both 'too hot' and 'too cold' temperatures were not included in the analysis.

Models are adjusted for trouble sleeping for any reason other than feeling too hot or feeling too cold, age (continuous), age^2, race and ethnicity (Hispanic/Latina, non-Hispanic Black, non-Hispanic White), marital status (married or living as though married, divorced/widowed/separated/never married), region of residence (Northeast, Midwest, South, West, and Puerto Rico), body mass index (BMI: underweight, recommended, overweight, obesity), and menopausal status (premenopausal, postmenopausal). In models for weekly sleep duration, sleep medication use, daytime dysfunction, and healthcare professional diagnosed sleep apnea, reasons for trouble sleeping for any reason other than feeling too hot or feeling too cold ≥ 3 times per week in the past month include: unable to fall asleep within 30 minutes, waking up in the middle of the night or early morning, waking up to use the bathroom, cannot breathe comfortably, coughing or snoring loudly, having bad dreams, having pain, or other non-specified reasons. In models for long sleep onset latency, poor sleep maintenance, and insomnia symptoms, reasons for trouble sleeping for any reason other than feeling too hot or feeling too cold ≥ 3 times per week in the past month include waking up to use the bathroom, cannot breathe comfortably, coughing or snoring loudly, having bad dreams, having pain, or other non-specified reasons.

[a] 'Too hot' is defined as self-reported trouble sleeping due to feeling too hot ≥ 3 times per week vs. trouble sleeping due to feeling too hot < 3 times per week and trouble sleeping due to feeling too cold < 3 times per week.

[b] 'Too cold' is defined as self-reported trouble sleeping due to feeling too cold ≥ 3 times per week vs. trouble sleeping due to feeling too hot < 3 times per week and trouble sleeping due to feeling too cold < 3 times per week.
[c] Sleep duration is based on reported bed and wake times or reported average sleep duration. Participants who reported ≤ 2 hours or ≥ 23 hours of sleep are excluded. Short: <7 hours; Recommended: 7-9 hours; Long >9 hours
[d] Long sleep onset latency is defined as not falling asleep within 30 minutes at least three times a week during the past month.
[e] Poor sleep maintenance is defined as waking up in the middle of the night or early morning at least three times a week during the past month.
[f] Insomnia is defined as long sleep onset latency or poor sleep maintenance.
[g] Sleep medication use is defined as 'taking medicine (prescription or over the counter) to help you sleep' at least three times a week during the past month.
[h] Daytime dysfunction is defined as having trouble staying awake while driving, eating, or engaging in social activity at least three times a week during the past month.
[i] Healthcare provider diagnosed sleep apnea is defined as a current doctor or other health professional diagnosis of sleep apnea.
[ⱡ] Remained significant after false discovery rate correction. False discovery rate-corrected P-value was considered statistically significant at the 0.05 level.

Supplemental Table 8B. Relative excess risk due to interaction (RERI) between perceived temperature extremes and annual household income, Sister Study, 2017-2019, N = 33,545

	**Indoor temperature: Too hot [a]**			**Indoor temperature: Too cold [b]**		
	<$20,000 - $49,999	$50,000 - $99,999	≥ $100,000	<$20,000 - $49,999	$50,000 - $99,999	≥ $100,000
Weekly sleep duration [c]						
Short	0.00 (-0.28, 0.28)	0.19 (-0.05, 0.43)		0.17 (-0.58, 0.92)	0.11 (-0.69, 0.91)	
Recommended	---	---	1.00 (Ref)	---	---	1.00 (Ref)
Long	-0.02 (-0.33, 0.29)	-0.15 (-0.41, 0.11)		-0.21 (-0.96, 0.54)	-0.71 (-1.45, 0.03)	
Long sleep onset latency [d]	---	---		---	---	
Yes	**0.58 (0.19, 0.97)**	**0.51 (0.18, 0.84)**	1.00 (Ref)	0.48 (-0.48, 1.44)	0.06 (-0.95, 1.07)	1.00 (Ref)
No	---	---		---	---	
Poor sleep maintenance [e]						
Yes	-0.08 (-0.18, 0.02)	**-0.09 (-0.18, -0.00)**	1.00 (Ref)	0.11 (-0.14, 0.36)	0.23 (-0.05, 0.51)	1.00 (Ref)
No	---	---		---	---	
Insomnia symptoms [f]						
Yes	-0.07 (-0.16, 0.02)	**-0.08 (-0.16, -0.00)**	1.00 (Ref)	0.06 (-0.18, 0.30)	0.14 (-0.13, 0.41)	1.00 (Ref)
No	---	---		---	---	
Sleep medication use [g]						
Yes	**0.24 (0.01, 0.47)**	0.10 (-0.09, 0.29)	1.00 (Ref)	-0.17 (-0.72, 0.38)	**-0.59 (-1.16, -0.02)**	1.00 (Ref)
No	---	---		---	---	
Daytime dysfunction [h]						
Yes	0.47 (-1.38, 2.32)	0.48 (-0.98, 1.94)	1.00 (Ref)	-1.41 (-7.74, 4.92)	3.29 (-4.77, 11.35)	1.00 (Ref)
No	---	---		---	---	
Healthcare professional diagnosed sleep apnea [i]						
Yes	0.12 (-0.19, 0.43)	0.05 (-0.23, 0.33)	1.00 (Ref)	0.03 (-0.81, 0.87)	0.12 (-0.82, 1.06)	1.00 (Ref)
No	---	---		---	---	

Relative Excess Risk due to Interaction (RERI) = 0: no additive interaction; RERI >0: positive additive interaction; RERI <0: negative additive interaction

Models are adjusted for trouble sleeping for any reason other than feeling too hot or feeling too cold, age (continuous), age^2, race and ethnicity (Hispanic/Latina, non-Hispanic Black, non-Hispanic White), marital status (married or living as though married, divorced/widowed/separated/never married), region of residence (Northeast, Midwest, South, West, and Puerto Rico), body mass index (BMI: underweight, recommended, overweight, obesity), and menopausal status (premenopausal, postmenopausal). In models for weekly sleep duration, sleep medication use, daytime dysfunction, and healthcare professional diagnosed sleep apnea, reasons for trouble sleeping for any reason other than feeling too hot or feeling too cold ≥ 3 times per week in the past month include: unable to fall asleep within 30 minutes, waking up in the middle of the night or early morning, waking up to use the bathroom, cannot breathe comfortably, coughing or snoring loudly, having bad dreams, having pain, or other non-specified reasons. In models for long sleep onset latency, poor sleep maintenance, and insomnia symptoms, reasons for trouble sleeping for any reason other than feeling too hot or feeling too cold ≥ 3 times per week in the past month include waking up to use the bathroom, cannot breathe comfortably, coughing or snoring loudly, having bad dreams, having pain, or other non-specified reasons.

[a] 'Too hot' is defined as self-reported trouble sleeping due to feeling too hot ≥ 3 times per week vs. trouble sleeping due to feeling too hot < 3 times per week and trouble sleeping due to feeling too cold < 3 times per week.
[b] 'Too cold' is defined as self-reported trouble sleeping due to feeling too cold ≥ 3 times per week vs. trouble sleeping due to feeling too hot < 3 times per week and trouble sleeping due to feeling too cold < 3 times per week.
[c] Sleep duration is based on reported bed and wake times or reported average sleep duration. Participants who reported ≤ 2 hours or ≥ 23 hours of sleep are excluded. Short: <7 hours; Recommended: 7-9 hours; Long >9 hours
[d] Long sleep onset latency is defined as not falling asleep within 30 minutes at least three times a week during the past month.
[e] Poor sleep maintenance is defined as waking up in the middle of the night or early morning at least three times a week during the past month.
[f] Insomnia is defined as long sleep onset latency or poor sleep maintenance.
[g] Sleep medication use is defined as 'taking medicine (prescription or over the counter) to help you sleep' at least three times a week during the past month.
[h] Daytime dysfunction is defined as having trouble staying awake while driving, eating, or engaging in social activity at least three times a week during the past month.
[i] Healthcare provider diagnosed sleep apnea is defined as a current doctor or other health professional diagnosis of sleep apnea.

Supplemental Table 9. Relative excess risk due to interaction (RERI) between perceived indoor temperature extremes and region of residence, Sister Study, 2017-2019, N=33,545

	Indoor temperature: Too hot [a]					Indoor temperature: Too cold [b]				
	**Northeast**	**Midwest**	**South**	**West**	**Puerto Rico**	**Northeast**	**Midwest**	**South**	**West**	**Puerto Rico**
Weekly sleep duration [c]										
Short	-0.11 (-0.41, 0.19)	**0.41 (0.15, 0.67)**		0.17 (-0.10, 0.44)	1.27 (-0.08, 2.62)	0.83 (-0.21, 1.87)	-0.04 (-0.77, 0.69)		-0.28 (-1.01, 0.45)	-0.52 (-1.51, 0.47)
Recommended	---	---	1.00 (Ref)	---	---	---	---	1.00 (Ref)	---	---
Long	-0.06 (-0.34, 0.22)	0.08 (-0.16, 0.32)		0.17 (-0.09, 0.43)	**1.45 (0.48, 2.42)**	-0.10 (-0.87, 0.67)	-0.28 (-0.87, 0.31)		0.29 (-0.35, 0.93)	-0.11 (-0.93, 0.71)
Long sleep onset latency [d]										
Yes	-0.27 (-0.63, 0.09)	**-0.32 (-0.63, -0.01)**	1.00 (Ref)	-0.28 (-0.61, 0.05)	-0.50 (-1.48, 0.48)	-0.12 (-1.04, 0.80)	0.47 (-0.31, 1.25)	1.00 (Ref)	0.19 (-0.60, 0.98)	0.37 (-0.75, 1.49)
No	---	---		---	---	---	---		---	---
Poor sleep maintenance [e]										
Yes	0.08 (-0.03, 0.19)	0.01 (-0.09, 0.11)	1.00 (Ref)	0.00 (-0.11, 0.11)	0.17 (-0.32, 0.66)	-0.18 (-0.55, 0.19)	0.06 (-0.22, 0.34)	1.00 (Ref)	-0.22 (-0.51, 0.07)	0.23 (-0.25, 0.71)
No	---	---		---	---	---	---		---	---
Insomnia symptoms [f]										
Yes	0.07 (-0.03, 0.17)	0.00 (-0.09, 0.09)	1.00 (Ref)	0.02 (-0.07, 0.11)	0.16 (-0.27, 0.59)	-0.22 (-0.56, 0.12)	0.03 (-0.22, 0.28)	1.00 (Ref)	-0.22 (-0.49, 0.05)	0.21 (-0.21, 0.63)
No	---	---		---	---	---	---		---	---
Sleep medication use [g]										
Yes	-0.03 (-0.27, 0.21)	-0.13 (-0.33, 0.07)	1.00 (Ref)	-0.17 (-0.38, 0.04)	0.14 (-1.06, 1.34)	0.40 (-0.23, 1.03)	0.22 (-0.29, 0.73)	1.00 (Ref)	-0.19 (-0.69, 0.31)	1.33 (-0.06, 2.72)
No	---	---		---	---	---	---		---	---
Daytime dysfunction [h]										
Yes	---	---	1.00 (Ref)	---	---	---	---	1.00 (Ref)	---	---
No	---	---		---	---	---	---		---	---
Healthcare professional diagnosed sleep apnea [i]										
Yes	-0.03 (-0.37, 0.31)	-0.16 (-0.45, 0.13)	1.00 (Ref)	-0.22 (-0.55, 0.11)	0.67 (-0.71, 2.05)	-0.49 (-1.40, 0.42)	**-0.96 (-1.73, -0.19)**	1.00 (Ref)	-0.06 (-0.99, 0.87)	0.93 (-0.68, 2.54)
No	---	---		---	---	---	---		---	---

Relative Excess Risk due to Interaction (RERI) = 0: no additive interaction; RERI >0: positive additive interaction; RERI <0: negative additive interaction

Models are adjusted for trouble sleeping for any reason other than feeling too hot or feeling too cold, age (continuous), age^2, race and ethnicity (Hispanic/Latina, non-Hispanic Black, non-Hispanic White), annual household income (<$20,000 - $49,999, $50,000 - $99,999, ≥$100,000), body mass index (BMI: underweight, recommended, overweight, obesity), marital status (married or living as though married, divorced/widowed/separated/never married), and menopausal status (premenopausal, postmenopausal). In models for weekly sleep duration, sleep medication use, daytime dysfunction, and healthcare professional diagnosed sleep apnea, reasons for trouble sleeping for any reason other than feeling too hot or feeling too cold ≥ 3 times per week in the past month include: unable to fall asleep within 30 minutes, waking up in the middle of the night or early morning, waking up to use the bathroom, cannot breathe comfortably, coughing or snoring loudly, having bad dreams, having pain, or other non-specified reasons. In models for long sleep onset latency, poor sleep maintenance, and insomnia symptoms, reasons for trouble sleeping for any reason other than feeling too hot or feeling too cold ≥ 3 times per week in the past month include waking up to use the bathroom, cannot breathe comfortably, coughing or snoring loudly, having bad dreams, having pain, or other non-specified reasons.

[a] 'Too hot' is defined as self-reported trouble sleeping due to feeling too hot ≥ 3 times per week vs. trouble sleeping due to feeling too hot < 3 times per week and trouble sleeping due to feeling too cold < 3 times per week.
[b] 'Too cold' is defined as self-reported trouble sleeping due to feeling too cold ≥ 3 times per week vs. trouble sleeping due to feeling too hot < 3 times per week and trouble sleeping due to feeling too cold < 3 times per week.
[c] Sleep duration is based on reported bed and wake times or reported average sleep duration. Participants who reported ≤ 2 hours or ≥ 23 hours of sleep are excluded. Short: <7 hours; Recommended: 7-9 hours; Long >9 hours
[d] Long sleep onset latency is defined as not falling asleep within 30 minutes at least three times a week during the past month.
[e] Poor sleep maintenance is defined as waking up in the middle of the night or early morning at least three times a week during the past month.
[f] Insomnia is defined as long sleep onset latency or poor sleep maintenance.
[g] Sleep medication use is defined as 'taking medicine (prescription or over the counter) to help you sleep' at least three times a week during the past month.
[h] Daytime dysfunction is defined as having trouble staying awake while driving, eating, or engaging in social activity at least three times a week during the past month.

[i] Healthcare provider diagnosed sleep apnea is defined as a current doctor or other health professional diagnosis of sleep apnea.

Supplemental Table 10. Relative excess risk due to interaction (RERI) between perceived indoor temperature extremes and menopausal status, Sister Study, 2017 – 2019, N = 33,545

	Indoor temperature: Too hot [a]		Indoor temperature: Too cold [b]	
	**Premenopausal**	**Postmenopausal**	**Premenopausal**	**Postmenopausal**
Weekly sleep duration [c]				
Short		0.12 (-0.25, 0.49)		-0.05 (-1.12, 1.02)
Recommended	1.0 (Ref)	---	1.0 (Ref)	---
Long		-0.13 (-0.65, 0.39)		-0.00 (-1.42, 1.42)
Long sleep onset latency [d]				
Yes	1.0 (Ref)	-0.10 (-0.77, 0.57)	1.0 (Ref)	**1.69 (0.43, 2.95)**
No		---		---
Poor sleep maintenance [e]				
Yes	1.0 (Ref)	-0.11 (-0.24, 0.02)	1.0 (Ref)	-0.07 (-0.54, 0.40)
No		---		---
Insomnia symptoms [f]				
Yes	1.0 (Ref)	-0.10 (-0.20, 0.00)	1.0 (Ref)	0.01 (-0.42, 0.44)
No		---		---
Sleep medication use [g]				
Yes	1.0 (Ref)	0.08 (-0.30, 0.46)	1.0 (Ref)	-0.44 (-1.67, 0.79)
No		---		---
Daytime dysfunction [h]				
Yes	1.0 (Ref)	1.29 (-0.94, 3.52)	1.0 (Ref)	-8.85 (-29.77, 12.87)
No		---		---
Healthcare professional diagnosed sleep apnea [i]				
Yes	1.0 (Ref)	0.24 (-0.26, 0.74)	1.0 (Ref)	0.56 (-0.99, 2.11)
No		---		---

Relative Excess Risk due to Interaction (RERI) = 0: no additive interaction; RERI >0: positive additive interaction; RERI <0: negative additive interaction

Models are adjusted for trouble sleeping for any reason other than feeling too hot or feeling too cold, age (continuous), age^2, race and ethnicity (Hispanic/Latina, non-Hispanic Black, non-Hispanic White), annual household income (<$20,000 - $49,999, $50,000 - $99,999, ≥$100,000), marital status (married or living as though married, divorced/widowed/separated/never married), region of residence (Northeast, Midwest, South, West, and Puerto Rico), and body mass index (BMI: underweight, recommended, overweight, obesity). In models for weekly sleep duration, sleep medication use, daytime dysfunction, and healthcare professional diagnosed sleep apnea, reasons for trouble sleeping for any reason other than feeling too hot or feeling too cold ≥ 3 times per week in the past month include: unable to fall asleep within 30 minutes, waking up in the middle of the night or early morning, waking up to use the bathroom, cannot breathe comfortably, coughing or snoring loudly, having bad dreams, having pain, or other non-specified reasons. In models for long sleep onset latency, poor sleep maintenance, and insomnia symptoms, reasons for trouble sleeping for any reason other than feeling too hot or feeling too cold ≥ 3 times per week in the past month include waking up to use the bathroom, cannot breathe comfortably, coughing or snoring loudly, having bad dreams, having pain, or other non-specified reasons.

[a] 'Too hot' is defined as self-reported trouble sleeping due to feeling too hot ≥ 3 times per week vs. trouble sleeping due to feeling too hot < 3 times per week and trouble sleeping due to feeling too cold < 3 times per week.

[b] 'Too cold' is defined as self-reported trouble sleeping due to feeling too cold ≥ 3 times per week vs. trouble sleeping due to feeling too hot < 3 times per week and trouble sleeping due to feeling too cold < 3 times per week.
[c] Sleep duration is based on reported bed and wake times or reported average sleep duration. Participants who reported ≤ 2 hours or ≥ 23 hours of sleep are excluded. Short: <7 hours; Recommended: 7-9 hours; Long >9 hours
[d] Long sleep onset latency is defined as not falling asleep within 30 minutes at least three times a week during the past month.
[e] Poor sleep maintenance is defined as waking up in the middle of the night or early morning at least three times a week during the past month.
[f] Insomnia is defined as long sleep onset latency or poor sleep maintenance.
[g] Sleep medication use is defined as 'taking medicine (prescription or over the counter) to help you sleep' at least three times a week during the past month.
[h] Daytime dysfunction is defined as having trouble staying awake while driving, eating, or engaging in social activity at least three times a week during the past month.
[i] Healthcare provider diagnosed sleep apnea is defined as a current doctor or other health professional diagnosis of sleep apnea.

Supplemental Table 11. Sociodemographic, clinical, and sleep health characteristics among participants who reported trouble sleeping for any reason [a], overall and by perceived indoor temperature extremes, Sister Study, 2017-2019, N = 23,853

	Total N = 23,853 (100.0)	Infrequent temperature extremes [b] n = 20,347 (85.3)	Too hot [c] n = 2,682 (11.2)	Too cold [d] n = 406 (1.7)
**Sociodemographic characteristics**				
Age, years (mean ± SD)	67.61 ± 8.45	68.31 ± 8.31	62.78 ± 7.65	68.90 ± 9.04
Age (categorical years)				
< 67.2	11,411 (47.8)	9,025 (44.4)	1,920 (71.6)	164 (40.4)
≥ 67.2	12,442 (52.2)	11,322 (55.6)	762 (28.4)	242 (59.6)
Race and ethnicity				
Hispanic/Latina	783 (3.3)	622 (3.1)	100 (3.7)	36 (8.9)
Non-Hispanic Black	1,494 (6.3)	1,150 (5.7)	276 (10.3)	31 (7.6)
Non-Hispanic White	21,576 (90.5)	18,575 (91.3)	2,306 (86.0)	339 (83.5)
Educational attainment [e]				
≤ High school or GED	3,205 (13.4)	2,684 (13.2)	384 (14.3)	79 (19.5)
Some college/technical	7,603 (31.9)	6,394 (31.4)	913 (34.0)	150 (36.9)
≥ Bachelor's degree or higher	13,042 (54.7)	11,267 (55.4)	1,385 (51.6)	177 (43.6)
Missing	3 (0.0)	2 (0.0)	0 (0.0)	0 (0.0)
Annual household income				
< $20,000 - $49,999	6,614 (27.7)	5,741 (28.2)	585 (21.8)	176 (43.3)
$50,000 - $99,999	8,820 (37.0)	7,605 (37.4)	943 (35.2)	119 (29.3)
≥ $100,000	8,419 (35.3)	7,001 (34.4)	1,154 (43.0)	111 (27.3)
Marital status				
Married or living as though married	16,124 (67.6)	13,508 (66.4)	2,065 (77.0)	251 (61.8)
Never married, divorced, widowed, or separated	7,729 (32.4)	6,839 (33.6)	617 (23.0)	155 (38.2)
Region of residence				
Northeast	3,876 (16.2)	3,334 (16.4)	430 (16.0)	55 (13.5)
Midwest	6,290 (26.4)	5,375 (26.4)	702 (26.2)	101 (24.9)
South	7,916 (33.2)	6,722 (33.0)	921 (34.3)	131 (32.3)
West	5,522 (23.2)	4,725 (23.2)	605 (22.6)	96 (23.6)
Puerto Rico	249 (1.0)	191 (0.9)	24 (0.9)	23 (5.7)
**Clinical characteristics**				
Body Mass Index, kg/m^2				
Recommended (18.5 - 24.9)	9,092 (38.1)	7,891 (38.8)	877 (32.7)	166 (40.9)

Overweight (25.0 - 29.9)	7,633 (32.0)	6,507 (32.0)	895 (33.4)	101 (24.9)
Obesity (≥ 30)	6,823 (28.6)	5,691 (28.0)	878 (32.7)	133 (32.8)
Missing	305 (1.3)	258 (1.3)	32 (1.2)	6 (1.5)
Menopausal status				
Premenopausal	791 (3.3)	588 (2.9)	158 (5.9)	20 (4.9)
Postmenopausal	23,057 (96.7)	19,754 (97.1)	2,524 (94.1)	386 (95.1)
Missing or never had period	5 (0.0)	5 (0.0)	0 (0.0)	0 (0.0)
**Sleep health dimensions**				
Average weekly sleep duration [f]				
Short (< 7)	2,844 (11.9)	2,348 (11.5)	369 (13.8)	67 (16.5)
Recommended (7-9)	16,898 (70.8)	14,567 (71.6)	1,825 (68.0)	242 (59.6)
Long (> 9)	3,819 (16.0)	3,188 (15.7)	461 (17.2)	83 (20.4)
Missing	292 (1.2)	244 (1.2)	27 (1.0)	14 (3.4)
Long sleep onset latency [g]				
Yes	4,183 (17.5)	3,197 (15.7)	689 (25.7)	120 (29.6)
No	19,462 (81.6)	16,977 (83.4)	1,973 (73.6)	278 (68.5)
Missing	208 (0.9)	173 (0.9)	20 (0.7)	8 (2.0)
Poor sleep maintenance [h]				
Yes	13,260 (55.6)	10,712 (52.6)	1,964 (73.2)	262 (64.5)
No	10,403 (43.6)	9,465 (46.5)	705 (26.3)	142 (35.0)
Missing	190 (0.8)	170 (0.8)	13 (0.5)	2 (0.5)
Insomnia symptoms [i]				
Yes	14,355 (60.2)	11,621 (57.1)	2,102 (78.4)	279 (68.7)
No	9,431 (39.5)	8,667 (42.6)	577 (21.5)	125 (30.8)
Missing	67 (0.3)	59 (0.3)	3 (0.1)	2 (0.5)
Sleep medication use [j]				
Yes	5,077 (21.3)	4,219 (20.7)	623 (23.2)	114 (28.1)
No	18,674 (78.3)	16,047 (78.9)	2,047 (76.3)	288 (70.9)
Missing	102 (0.4)	81 (0.4)	12 (0.4)	4 (1.0)
Daytime dysfunction [k]				
Yes	219 (0.9)	155 (0.8)	36 (1.3)	16 (3.9)
No	23,356 (97.9)	19,953 (98.1)	2,615 (97.5)	386 (95.1)
Missing	278 (1.2)	239 (1.2)	31 (1.2)	4 (1.0)
Healthcare provider diagnosed sleep apnea [l]				
Yes	2,564 (10.7)	2,117 (10.4)	325 (12.1)	71 (17.5)

No	21,023 (88.1)	18,002 (88.5)	2,338 (87.2)	324 (79.8)
Missing	266 (1.1)	228 (1.1)	19 (0.7)	11 (2.7)

Abbreviations: SD, Standard Deviation; GED: General Educational Development
Note: N = 418 (1.8%) participants who reported both 'too hot' and 'too cold' temperatures were not included in this sensitivity analysis.
[a] Reasons for trouble sleeping ≥ 3 times per week in the past month include: unable to fall asleep within 30 minutes, waking up in the middle of the night or early morning, waking up to use the bathroom, cannot breathe comfortably, coughing or snoring loudly, having bad dreams, having pain, or other non-specified reasons.
[b] ‘'Infrequent temperature extremes’ is defined as self-reported trouble sleeping due to feeling too hot < 3 times per week and trouble sleeping due to feeling too cold < 3 times per week in the past month.
[c] ’Too hot’ is defined as self-reported trouble sleeping due to feeling too hot ≥ 3 times per week in the past month.
[d] ’Too cold’ is defined as self-reported trouble sleeping due to feeling too cold ≥ 3 times per week in the past month.
[e] Educational attainment was assessed at baseline.
[f] Sleep duration is based on reported bed and wake times or reported average sleep duration. Participants who reported ≤ 2 or ≥ 23 hours of sleep are excluded. Short: < 7 hours; Recommended: 7-9 hours; Long ≥ 9 hours.
[g] Long sleep onset latency is defined as not falling asleep within 30 minutes at least three times a week during the past month.
[h] Poor sleep maintenance is defined as waking up in the middle of the night or early morning at least three times a week during the past month.
[i] Insomnia symptoms is defined as long sleep onset latency or poor sleep maintenance.
[j] Sleep medication use is defined as 'taking medicine (prescription or over the counter) to help you sleep' at least three times a week during the past month.
[k] Daytime dysfunction is defined as having trouble staying awake while driving, eating, or engaging in social activity at least three times a week during the past month.
[l] Healthcare provider diagnosed sleep apnea is defined as a current doctor or other health professional diagnosis of sleep apnea.

Supplemental Table 12. Sociodemographic, clinical, and sleep health characteristics among participants who reported trouble sleeping for any reason [a], by race and ethnicity as well as perceived indoor temperature extremes, Sister Study, 2017-2019, N = 23,853

Race and ethnicity	Hispanic/Latina [b]				Non-Hispanic Black [c]				Non-Hispanic White [d]			
	Total n =783 (3.3%)	Infrequent temperature extremes [e] n = 622 (79.4%)	Too hot [f ¥] n = 100 (12.8%)	Too cold [g §] n = 36 (4.6%)	Total n = 1,494 (6.3%)	Infrequent temperature extremes [e] n = 1,150 (77.0%)	Too hot [f ¥] n = 276 (18.5%)	Too cold [g §] n = 31 (2.1%)	Total n = 21,576 (90.5%)	Infrequent temperature extremes [e] n = 18,575 (86.1%)	Too hot [f ¥] n = 2,306 (10.7%)	Too cold [g §] n = 339 (1.6%)
**Sociodemographic characteristics**												
Age, years [ƚ]	64.20 ± 8.55	64.85 ± 8.50	60.35 ± 7.80	66.00 ± 8.96	65.68 ± 8.16	66.60 ± 8.03	62.49 ± 7.62	65.04 ± 10.43	67.86 ± 8.42	68.53 ± 8.29	62.92 ± 7.63	69.56 ± 8.78
Age, years [ƚ]												
< 67.2	502 (64.1)	381 (61.3)	82 (82.0)	21 (58.3)	839 (56.2)	599 (52.1)	197 (71.4)	15 (48.4)	10,070 (46.7)	8,045 (43.3)	1,641 (71.2)	128 (37.8)
≥ 67.2	281 (35.9)	241 (38.7)	18 (18.0)	15 (41.7)	655 (43.8)	551 (47.9)	79 (28.6)	16 (51.6)	11,506 (53.3)	10,530 (56.7)	665 (28.8)	211 (62.2)
Educational attainment [ƚ h]												
< High school or GED	143 (18.3)	108 (17.4)	19 (19.0)	9 (25.0)	121 (8.1)	88 (7.7)	25 (9.1)	7 (22.6)	2,941 (13.6)	2,488 (13.4)	340 (14.7)	63 (18.6)
Some college / technical	256 (32.7)	196 (31.5)	37 (37.0)	11 (30.6)	469 (31.4)	344 (29.9)	101 (36.6)	10 (32.3)	6,878 (31.9)	5,854 (31.5)	775 (33.6)	129 (38.1)
≥ Bachelor's or higher	384 (49.0)	318 (51.1)	44 (44.0)	16 (44.4)	904 (60.5)	718 (62.4)	150 (54.3)	14 (45.2)	11,754 (54.5)	10,231 (55.1)	1,191 (51.6)	147 (43.4)
Missing	*0 (0.0)*	*0 (0.0)*	*0 (0.0)*	*0 (0.0)*	*0 (0.0)*	*0 (0.0)*	*0 (0.0)*	*0 (0.0)*	*3 (0.0)*	*2 (0.0)*	*0 (0.0)*	*0 (0.0)*
Annual household income [ƚ]												
< $20,000 - $49,999	386 (49.3)	301 (48.4)	38 (38.0)	31 (86.1)	536 (35.9)	426 (37.0)	79 (28.6)	16 (51.6)	5,692 (26.4)	5,014 (27.0)	468 (20.3)	129 (38.1)
$50,000 - $99,999	209 (26.7)	170 (27.3)	29 (29.0)	3 (8.3)	568 (38.0)	427 (37.1)	121 (43.8)	6 (19.4)	8,043 (37.3)	7,008 (37.7)	793 (34.4)	110 (32.4)
≥ $100,000	188 (24.0)	151 (24.3)	33 (33.0)	2 (5.6)	390 (26.1)	297 (25.8)	76 (27.5)	9 (29.0)	7,841 (36.3)	6,553 (35.3)	1,045 (45.3)	100 (29.5)
Marital status [ƚ]												
Married or living as though married	499 (63.7)	388 (62.4)	74 (74.0)	18 (50.0)	681 (45.6)	499 (43.4)	157 (56.9)	9 (29.0)	14,944 (69.3)	12,621 67.9)	1,834 (79.5)	224 (66.1)
Never married, divorced, widowed, or separated	284 (36.3)	234 (37.6)	26 (26.0)	18 (50.0)	813 (54.4)	651 (56.6)	119 (43.1)	22 (71.0)	6,632 (30.7)	5,954 (32.1)	472 (20.5)	115 (33.9)
Region of residence [ƚ]												
Northeast	63 (8.0)	45 (7.2)	14 (14.0)	1 (2.8)	129 (8.6)	102 (8.9)	22 (8.0)	2 (6.5)	3,684 (17.1)	3,187 (17.2)	394 (17.1)	52 (15.3)
Midwest	47 (6.0)	41 (6.6)	6 (6.0)	0 (0.0)	284 (19.0)	223 (19.4)	50 (18.1)	6 (19.4)	5,959 (27.6)	5,111 (27.5)	646 (28.0)	95 (28.0)
South	212 (27.1)	172 (27.7)	28 (28.0)	7 (19.4)	953 (63.8)	718 (62.4)	189 (68.5)	18 (58.1)	6,751 (31.3)	5,832 (31.4)	704 (30.5)	106 (31.3)
West	215 (27.5)	176 (28.3)	28 (28.0)	5 (13.9)	128 (8.6)	107 (9.3)	15 (5.4)	5 (16.1)	5,179 (24.0)	4,442 (23.9)	562 (24.4)	86 (25.4)
Puerto Rico	246 (31.4)	188 (30.2)	24 (24.0)	23 (63.9)	0 (0.0)	0 (0.0)	0 (0.0)	0 (0.0)	3 (0.0)	3 (0.0)	0 (0.0)	0 (0.0)
**Clinical characteristics**												
Body Mass Index, kg/m^2 [ƚ]												
Recommended (18.5 - 24.9)	241 (30.8)	196 (31.5)	30 (30.0)	10 (27.8)	233 (15.6)	182 (15.8)	42 (15.2)	5 (16.1)	8,618 (39.9)	7,513 (40.4)	805 (34.9)	151 (44.5)
Overweight (25.0 - 29.9)	273 (34.9)	215 (34.6)	33 (33.0)	14 (38.9)	488 (32.7)	385 (33.5)	79 (28.6)	9 (29.0)	6,872 (31.9)	5,907 (31.8)	783 (34.0)	78 (23.0)
Obesity (≥ 30)	261 (33.3)	205 (33.0)	36 (36.0)	12 (33.3)	756 (50.6)	570 (49.6)	154 (55.8)	16 (51.6)	5,806 (26.9)	4,916 (26.5)	688 (29.8)	105 (31.0)
Missing	*8 (1.0)*	*6 (1.0)*	*1 (1.0)*	*0 (0.0)*	*17 (1.1)*	*13 (1.1)*	*1 (0.4)*	*1 (3.2)*	*280 (1.3)*	*239 (1.3)*	*30 (1.3)*	*5 (1.5)*
Menopausal status [ƚ]												
Premenopausal	50 (6.4)	42 (6.8)	7 (7.0)	1 (2.8)	85 (5.7)	53 (4.6)	24 (8.7)	5 (16.1)	656 (3.0)	493 (2.7)	127 (5.5)	14 (4.1)
Postmenopausal	733 (93.6)	580 (93.2)	93 (93.0)	35 (97.2)	1,407 (94.2)	1,095 (95.2)	252 (91.3)	26 (83.9)	20,917 (96.9)	18,079 (97.3)	2,179 (94.5)	325 (95.9)

Missing or never had a period	*0 (0.0)*	*0 (0.0)*	*0 (0.0)*	*0 (0.0)*	*2 (0.1)*	*2 (0.2)*	*0 (0.0)*	*0 (0.0)*	*3 (0.0)*	*3 (0.0)*	*0 (0.0)*	*0 (0.0)*
**Sleep health dimensions**												
Average weekly sleep duration [ᵼ i]												
Short	128 (16.3)	102 (16.4)	17 (17.0)	4 (11.1)	450 (30.1)	336 (29.2)	84 (30.4)	14 (45.2)	2,266 (10.5)	1,910 (10.3)	268 (11.6)	49 (14.5)
Recommended	502 (64.1)	408 (65.6)	60 (60.0)	22 (61.1)	817 (54.7)	639 (55.6)	151 (54.7)	12 (38.7)	15,579 (72.2)	13,520 (72.8)	1,614 (70.0)	208 (61.4)
Long	132 (16.9)	99 (15.9)	20 (20.0)	6 (16.7)	195 (13.1)	153 (13.3)	33 (12.0)	4 (12.9)	3,492 (16.2)	2,936 (15.8)	408 (17.7)	73 (21.5)
Missing	*21 (2.7)*	*13 (2.1)*	*3 (3.0)*	*4 (11.1)*	*32 (2.1)*	*22 (1.9)*	*8 (2.9)*	*1 (3.2)*	*239 (1.1)*	*209 (1.1)*	*16 (0.7)*	*9 (2.7)*
Long sleep onset latency [ᵼ j]												
Yes	216 (27.6)	146 (23.5)	41 (41.0)	14 (38.9)	327 (21.9)	210 (18.3)	90 (32.6)	7 (22.6)	3,640 (16.9)	2,841 (15.3)	558 (24.2)	99 (29.2)
No	545 (69.6)	462 (74.3)	55 (55.0)	19 (52.8)	1,144 (76.6)	920 (80.0)	184 (66.7)	23 (74.2)	17,773 (82.4)	15,595 (84.0)	1,734 (75.2)	236 (69.6)
Missing	*22 (2.8)*	*14 (2.3)*	*4 (4.0)*	*3 (8.3)*	*23 (1.5)*	*20 (1.7)*	*2 (0.7)*	*1 (3.2)*	*163 (0.8)*	*139 (0.7)*	*14 (0.6)*	*4 (1.2)*
Poor sleep maintenance [k]												
Yes	410 (52.4)	300 (48.2)	68 (68.0)	23 (63.9)	793 (53.1)	568 (49.4)	179 (64.9)	19 (61.3)	12,057 (55.9)	9,844 (53.0)	1,717 (74.5)	220 (64.9)
No	355 (45.3)	308 (49.5)	31 (31.0)	11 (30.6)	685 (45.9)	569 (49.5)	94 (34.1)	12 (38.7)	9,363 (43.4)	8,588 (46.2)	580 (25.2)	119 (35.1)
Missing	*18 (2.3)*	*14 (2.3)*	*1 (1.0)*	*2 (5.6)*	*16 (1.1)*	*13 (1.1)*	*3 (1.1)*	*0 (0.0)*	*156 (0.7)*	*143 (0.8)*	*9 (0.4)*	*0 (0.0)*
Insomnia symptoms [l]												
Yes	465 (59.4)	342 (55.0)	79 (79.0)	25 (69.4)	864 (57.8)	616 (53.6)	195 (70.7)	21 (67.7)	13,026 (60.4)	10,663 (57.4)	1,828 (79.3)	233 (68.7)
No	305 (39.0)	271 (43.6)	20 (20.0)	9 (25.0)	625 (41.8)	529 (46.0)	81 (29.3)	10 (32.3)	8,501 (39.4)	7,867 (42.4)	476 (20.6)	106 (31.3)
Missing	*13 (1.7)*	*9 (1.4)*	*1 (1.0)*	*2 (5.6)*	*5 (0.3)*	*5 (0.4)*	*0 (0.0)*	*0 (0.0)*	*49 (0.2)*	*45 (0.2)*	*2 (0.1)*	*0 (0.0)*
Sleep medication use [ᵼ m]												
Yes	152 (19.4)	113 (18.2)	18 (18.0)	10 (27.8)	204 (13.7)	151 (13.1)	40 (14.5)	5 (16.1)	4,721 (21.9)	3,955 (21.3)	565 (24.5)	99 (29.2)
No	615 (78.5)	499 (80.2)	82 (82.0)	23 (63.9)	1,280 (85.7)	993 (86.3)	233 (84.4)	26 (83.9)	16,779 (77.8)	14,555 (78.4)	1,732 (75.1)	239 (70.5)
Missing	*16 (2.0)*	*10 (1.6)*	*0 (0.0)*	*3 (8.3)*	*10 (0.7)*	*6 (0.5)*	*3 (1.1)*	*0 (0.0)*	*76 (0.4)*	*65 (0.3)*	*9 (0.4)*	*1 (0.3)*
Daytime dysfunction [n]												
Yes	13 (1.7)	8 (1.3)	1 (1.0)	2 (5.6)	16 (1.1)	9 (0.8)	4 (1.4)	3 (9.7)	190 (0.9)	138 (0.7)	31 (1.3)	11 (3.2)
No	754 (96.3)	601 (96.6)	98 (98.0)	33 (91.7)	1,463 (97.9)	1,130 (98.3)	268 (97.1)	28 (90.3)	21,139 (98.0)	18,222 (98.1)	2,249 (97.5)	325 (95.9)
Missing	*16 (2.0)*	*13 (2.1)*	*1 (1.0)*	*1 (2.8)*	*15 (1.0)*	*11 (1.0)*	*4 (1.4)*	*0 (0.0)*	*247 (1.1)*	*215 (1.2)*	*26 (1.1)*	*3 (0.9)*
Healthcare provider diagnosed sleep apnea [ᵼ o]												
Yes	77 (9.8)	55 (8.8)	12 (12.0)	7 (19.4)	239 (16.0)	170 (14.8)	51 (18.5)	9 (29.0)	2,248 (10.4)	1,892 (10.2)	262 (11.4)	55 (16.2)
No	693 (88.5)	558 (89.7)	88 (88.0)	28 (77.8)	1,225 (82.0)	956 (83.1)	220 (79.7)	21 (67.7)	19,105 (88.5)	16,488 (88.8)	2,030 (88.0)	275 (81.1)
Missing	*13 (1.7)*	*9 (1.4)*	*0 (0.0)*	*1 (2.8)*	*30 (2.0)*	*24 (2.1)*	*5 (1.8)*	*1 (3.2)*	*223 (1.0)*	*195 (1.0)*	*14 (0.6)*	*9 (2.7)*

Abbreviations: SD, Standard Deviation; GED: General Educational Development

Note: N = 418 (1.8%) participants who reported both 'too hot' and 'too cold' temperatures were not included in this sensitivity analysis.

[¥] Chi-square test p-values indicate significant differences between the overall prevalence of ‘too hot’ by race and ethnicity.

[§]Chi-square test p-values indicate significant differences between the overall prevalence of ‘too cold’ by race and ethnicity.

[ᵼ] Chi-square or Analysis of Variance (ANOVA) test p-values indicate significant differences in characteristics between the total populations of each race and ethnicity.

[a] Reasons for trouble sleeping ≥ 3 times per week in the past month include: unable to fall asleep within 30 minutes, waking up in the middle of the night or early morning, waking up to use the bathroom, cannot breathe comfortably, coughing or snoring loudly, having bad dreams, having pain, or other non-specified reasons.

[b] Excluded for reporting both ‘Too hot’ and ‘Too cold’, Hispanic/Latina: n = 25 (3.2%).

[c] Excluded for reporting both ‘Too hot’ and ‘Too cold’, Non-Hispanic Black: n = 37 (2.5%).

[d] Excluded for reporting both ‘Too hot’ and ‘Too cold’, Non-Hispanic White: n = 356 (1.6%).

[e] ‘Infrequent temperature extremes’ is defined as self-reported trouble sleeping due to feeling too hot < 3 times per week and trouble sleeping due to feeling too cold < 3 times per week in the past month.

[f] ’Too hot’ is defined as self-reported trouble sleeping due to feeling too hot ≥ 3 times per week in the past month.

[g] ’Too cold’ is defined as self-reported trouble sleeping due to feeling too cold ≥ 3 times per week in the past month.

[h] Educational attainment was assessed at baseline.
[i] Sleep duration is based on reported bed and wake times or reported average sleep duration. Participants who reported ≤ 2 or ≥ 23 hours of sleep are excluded. Short: < 7 hours; Recommended: 7-9 hours; Long ≥ 9 hours.
[j] Long sleep onset latency is defined as not falling asleep within 30 minutes at least three times a week during the past month.
[k] Poor sleep maintenance is defined as waking up in the middle of the night or early morning at least three times a week during the past month.
[l] Insomnia symptoms is defined as long sleep onset latency or poor sleep maintenance.
[m] Sleep medication use is defined as 'taking medicine (prescription or over the counter) to help you sleep' at least three times a week during the past month.
[n] Daytime dysfunction is defined as having trouble staying awake while driving, eating, or engaging in social activity at least three times a week during the past month.
[o] Healthcare provider diagnosed sleep apnea is defined as a current doctor or other health professional diagnosis of sleep apnea.

Supplemental Table 13. Sociodemographic, clinical, and sleep health characteristics among participants who reported trouble sleeping for any reason [a], by annual household income as well as perceived indoor temperature extremes, Sister Study, 2017-2019, N = 23,853

**Annual household income**	**< $20,000 - $49,999 [b]**				**$50,000 - $99,999 [c]**				**≥ $100,000 [d]**			
	Total n = 6,614 (27.7%)	Infrequent temperature extremes [e] n = 5,741 (86.8%)	Too hot [f ¥] n = 585 (8.8%)	Too cold [g §] n = 176 (2.7%)	Total n = 8,820 (37.0%)	Infrequent temperature extremes [e] n = 7,605 (86.2%)	Too hot [f ¥] n = 943 (10.7%)	Too cold [g §] n = 119 (1.3%)	Total n = 8,419 (35.3%)	Infrequent temperature extremes [e] n = 7,001 (83.2%)	Too hot [f ¥] n = 1,154 (13.7%)	Too cold [g §] n = 111 (1.3%)
**Sociodemographic characteristics**												
Age, years (mean ± SD) [ᵻ]	70.79 ± 8.14	71.43 ± 7.88	65.33 ± 7.97	71.39 ± 8.96	68.07 ± 7.96	68.70 ± 7.83	63.81 ± 7.33	67.74 ± 8.47	64.62 ± 8.18	65.33 ± 8.14	60.64 ± 7.16	66.20 ± 8.79
Age, years [ᵻ]												
< 67.2	2,154 (32.6)	1,682 (29.3)	348 (59.5)	56 (31.8)	3,992 (45.3)	3,207 (42.2)	626 (66.4)	52 (43.7)	5,265 (62.5)	4,136 (59.1)	946 (82.0)	56 (50.5)
≥ 67.2	4,460 (67.4)	4,059 (70.7)	237 (40.5)	120 (68.2)	4,828 (54.7)	4,398 (57.8)	317 (33.6)	67 (56.3)	3,154 (37.5)	2,865 (40.9)	208 (18.0)	55 (49.5)
Race and ethnicity [ᵻ]												
Hispanic/Latina	386 (5.8)	301 (5.2)	38 (6.5)	31 (17.6)	209 (2.4)	170 (2.2)	29 (3.1)	3 (2.5)	188 (2.2)	151 (2.2)	33 (2.9)	2 (1.8)
Non-Hispanic Black	536 (8.1)	426 (7.4)	79 (13.5)	16 (9.1)	568 (6.4)	427 (5.6)	121 (12.8)	6 (5.0)	390 (4.6)	297 (4.2)	76 (6.6)	9 (8.1)
Non-Hispanic White	5,692 (86.1)	5,014 (87.3)	468 (80.0)	129 (73.3)	8,043 (91.2)	7,008 (92.1)	793 (84.1)	110 (92.4)	7,841 (93.1)	6,553 (93.6)	1,045 (90.6)	100 (90.1)
Missing	386 (5.8)	301 (5.2)	38 (6.5)	31 (17.6)	209 (2.4)	170 (2.2)	29 (3.1)	3 (2.5)	188 (2.2)	151 (2.2)	33 (2.9)	2 (1.8)
Educational attainment [ᵻ h]												
<High school or GED	1,600 (24.2)	1,362 (23.7)	158 (27.0)	51 (29.0)	1,140 (12.9)	957 (12.6)	143 (15.2)	18 (15.1)	465 (5.5)	365 (5.2)	83 (7.2)	10 (9.0)
Some college / technical	2,678 (40.5)	2,318 (40.4)	242 (41.4)	71 (40.3)	2,986 (33.9)	2,509 (33.0)	370 (39.2)	47 (39.5)	1,939 (23.0)	1,567 (22.4)	301 (26.1)	32 (28.8)
≥ Bachelor's or higher	2,334 (35.3)	2,059 (35.9)	185 (31.6)	54 (30.7)	4,694 (53.2)	4,139 (54.4)	430 (45.6)	54 (45.4)	6,014 (71.4)	5,069 (72.4)	770 (66.7)	69 (62.2)
Missing	2 (0.0)	2 (0.0)	0 (0.0)	0 (0.0)	0 (0.0)	0 (0.0)	0 (0.0)	0 (0.0)	1 (0.0)	0 (0.0)	0 (0.0)	0 (0.0)
Marital status [ᵻ]												
Married or living as though married	2,531 (38.3)	2,141 (37.3)	278 (47.5)	67 (38.1)	6,221 (70.5)	5,279 (69.4)	735 (77.9)	87 (73.1)	7,372 (87.6)	6,088 (87.0)	1,052 (91.2)	97 (87.4)
Never married, divorced, widowed, or separated	4,083 (61.7)	3,600 (62.7)	307 (52.5)	109 (61.9)	2,599 (29.5)	2,326 (30.6)	208 (22.1)	32 (26.9)	1,047 (12.4)	913 (13.0)	102 (8.8)	14 (12.6)
Region of residence [ᵻ]												
Northeast	905 (13.7)	797 (13.9)	81 (13.8)	18 (10.2)	1,343 (15.2)	1,192 (15.7)	113 (12.0)	20 (16.8)	1,628 (19.3)	1,345 (19.2)	236 (20.5)	17 (15.3)
Midwest	1,893 (28.6)	1,667 (29.0)	148 (25.3)	50 (28.4)	2,475 (28.1)	2,124 (27.9)	276 (29.3)	27 (22.7)	1,922 (22.8)	1,584 (22.6)	278 (24.1)	24 (21.6)
South	2,184 (33.0)	1,882 (32.8)	212 (36.2)	48 (27.3)	2,916 (33.1)	2,486 (32.7)	336 (35.6)	41 (34.5)	2,816 (33.4)	2,354 (33.6)	373 (32.3)	42 (37.8)
West	1,433 (21.7)	1,247 (21.7)	125 (21.4)	38 (21.6)	2,046 (23.2)	1,768 (23.2)	215 (22.8)	30 (25.2)	2,043 (24.3)	1,710 (24.4)	265 (23.0)	28 (25.2)
Puerto Rico	199 (3.0)	148 (2.6)	19 (3.2)	22 (12.5)	40 (0.5)	35 (0.5)	3 (0.3)	1 (0.8)	10 (0.1)	8 (0.1)	2 (0.2)	0 (0.0)
**Clinical characteristics**												
Body Mass Index, kg/m^2 [ᵻ]												
Recommended (18.5 - 24.9)	2,076 (31.4)	1,844 (32.1)	148 (25.3)	59 (33.5)	3,294 (37.3)	2,884 (37.9)	299 (31.7)	54 (45.4)	3,722 (44.2)	3,163 (45.2)	430 (37.3)	53 (47.7)
Overweight (25.0 - 29.9)	2,099 (31.7)	1,827 (31.8)	187 (32.0)	48 (27.3)	2,877 (32.6)	2,498 (32.8)	313 (33.2)	23 (19.3)	2,657 (31.6)	2,182 (31.2)	395 (34.2)	30 (27.0)
Obesity (≥ 30)	2,343 (35.4)	1,984 (34.6)	245 (41.9)	66 (37.5)	2,536 (28.8)	2,130 (28.0)	317 (33.6)	41 (34.5)	1,944 (23.1)	1,577 (22.5)	316 (27.4)	26 (23.4)
Missing	96 (1.5)	86 (1.5)	5 (0.9)	3 (1.7)	113 (1.3)	93 (1.2)	14 (1.5)	1 (0.8)	96 (1.1)	79 (1.1)	13 (1.1)	2 (1.8)
Menopausal status [ᵻ]												
Premenopausal	91 (1.4)	67 (1.2)	18 (3.1)	1 (0.6)	209 (2.4)	144 (1.9)	48 (5.1)	9 (7.6)	491 (5.8)	377 (5.4)	92 (8.0)	10 (9.0)
Postmenopausal	6,520 (98.6)	5,671 (98.8)	567 (96.9)	175 (99.4)	8,610 (97.6)	7,460 (98.1)	895 (94.9)	110 (92.4)	7,927 (94.2)	6,623 (94.6)	1,062 (92.0)	101 (91.0)

Missing or never had a period	3 (0.0)	3 (0.1)	0 (0.0)	0 (0.0)	1 (0.0)	1 (0.0)	0 (0.0)	0 (0.0)	1 (0.0)	1 (0.0)	0 (0.0)	0 (0.0)
**Sleep health dimensions**												
Average weekly sleep duration [ǂ i]												
Short	932 (14.1)	792 (13.8)	85 (14.5)	33 (18.8)	1,027 (11.6)	840 (11.0)	147 (15.6)	19 (16.0)	885 (10.5)	716 (10.2)	137 (11.9)	15 (13.5)
Recommended	4,255 (64.3)	3,748 (65.3)	362 (61.9)	94 (53.4)	6,327 (71.7)	5,510 (72.5)	638 (67.7)	81 (68.1)	6,316 (75.0)	5,309 (75.8)	825 (71.5)	67 (60.4)
Long	1,280 (19.4)	1,084 (18.9)	125 (21.4)	38 (21.6)	1,384 (15.7)	1,182 (15.5)	151 (16.0)	18 (15.1)	1,155 (13.7)	922 (13.2)	185 (16.0)	27 (24.3)
Missing	147 (2.2)	117 (2.0)	13 (2.2)	11 (6.3)	82 (0.9)	73 (1.0)	7 (0.7)	1 (0.8)	63 (0.7)	54 (0.8)	7 (0.6)	2 (1.8)
Long sleep onset latency [ǂ j]												
Yes	1,420 (21.5)	1,106 (19.3)	192 (32.8)	64 (36.4)	1,561 (17.7)	1,186 (15.6)	274 (29.1)	31 (26.1)	1,202 (14.3)	905 (12.9)	223 (19.3)	25 (22.5)
No	5,099 (77.1)	4,559 (79.4)	382 (65.3)	107 (60.8)	7,196 (81.6)	6,361 (83.6)	666 (70.6)	88 (73.9)	7,167 (85.1)	6,057 (86.5)	925 (80.2)	83 (74.8)
Missing	95 (1.4)	76 (1.3)	11 (1.9)	5 (2.8)	63 (0.7)	58 (0.8)	3 (0.3)	0 (0.0)	50 (0.6)	39 (0.6)	6 (0.5)	3 (2.7)
Poor sleep maintenance [ǂ k]												
Yes	3,634 (54.9)	3,011 (52.4)	426 (72.8)	112 (63.6)	4,832 (54.8)	3,957 (52.0)	677 (71.8)	82 (68.9)	4,794 (56.9)	3,744 (53.5)	861 (74.6)	68 (61.3)
No	2,900 (43.8)	2,657 (46.3)	157 (26.8)	62 (35.2)	3,926 (44.5)	3,592 (47.2)	262 (27.8)	37 (31.1)	3,577 (42.5)	3,216 (45.9)	286 (24.8)	43 (38.7)
Missing	80 (1.2)	73 (1.3)	2 (0.3)	2 (1.1)	62 (0.7)	56 (0.7)	4 (0.4)	0 (0.0)	48 (0.6)	41 (0.6)	7 (0.6)	0 (0.0)
Insomnia symptoms [l]												
Yes	3,961 (59.9)	3,288 (57.3)	458 (78.3)	120 (68.2)	5,263 (59.7)	4,316 (56.8)	731 (77.5)	85 (71.4)	5,131 (60.9)	4,017 (57.4)	913 (79.1)	74 (66.7)
No	2,619 (39.6)	2,425 (42.2)	126 (21.5)	54 (30.7)	3,538 (40.1)	3,270 (43.0)	212 (22.5)	34 (28.6)	3,274 (38.9)	2,972 (42.5)	239 (20.7)	37 (33.3)
Missing	34 (0.5)	28 (0.5)	1 (0.2)	2 (1.1)	19 (0.2)	19 (0.2)	0 (0.0)	0 (0.0)	14 (0.2)	12 (0.2)	2 (0.2)	0 (0.0)
Sleep medication use [ǂ m]												
Yes	1,531 (23.1)	1,276 (22.2)	160 (27.4)	55 (31.3)	1,896 (21.5)	1,595 (21.0)	229 (24.3)	25 (21.0)	1,650 (19.6)	1,348 (19.3)	234 (20.3)	34 (30.6)
No	5,038 (76.2)	4,430 (77.2)	422 (72.1)	118 (67.0)	6,893 (78.2)	5,986 (78.7)	708 (75.1)	93 (78.2)	6,743 (80.1)	5,631 (80.4)	917 (79.5)	77 (69.4)
Missing	45 (0.7)	35 (0.6)	3 (0.5)	3 (1.7)	31 (0.4)	24 (0.3)	6 (0.6)	1 (0.8)	26 (0.3)	22 (0.3)	3 (0.3)	0 (0.0)
Daytime dysfunction [ǂ n]												
Yes	82 (1.2)	63 (1.1)	10 (1.7)	5 (2.8)	71 (0.8)	48 (0.6)	13 (1.4)	7 (5.9)	66 (0.8)	44 (0.6)	13 (1.1)	4 (3.6)
No	6,447 (97.5)	5,602 (97.6)	570 (97.4)	169 (96.0)	8,635 (97.9)	7,460 (98.1)	915 (97.0)	111 (93.3)	8,274 (98.3)	6,891 (98.4)	1,130 (97.9)	106 (95.5)
Missing	85 (1.3)	76 (1.3)	5 (0.9)	2 (1.1)	114 (1.3)	97 (1.3)	15 (1.6)	1 (0.8)	79 (0.9)	66 (0.9)	11 (1.0)	1 (0.9)
Healthcare provider diagnosed sleep apnea [ǂ o]												
Yes	879 (13.3)	737 (12.8)	94 (16.1)	33 (18.8)	942 (10.7)	780 (10.3)	113 (12.0)	22 (18.5)	743 (8.8)	600 (8.6)	118 (10.2)	16 (14.4)
No	5,615 (84.9)	4,906 (85.5)	481 (82.2)	136 (77.3)	7,783 (88.2)	6,740 (88.6)	826 (87.6)	93 (78.2)	7,625 (90.6)	6,356 (90.8)	1,031 (89.3)	95 (85.6)
Missing	120 (1.8)	98 (1.7)	10 (1.7)	7 (4.0)	95 (1.1)	85 (1.1)	4 (0.4)	4 (3.4)	51 (0.6)	45 (0.6)	5 (0.4)	0 (0.0)

Abbreviations: SD, Standard Deviation; GED: General Educational Development

Note: N = 418 (1.8%) participants who reported both 'too hot' and 'too cold' temperatures were not included in this sensitivity analysis.

[¥] Chi-square test *p*-values indicate significant differences between the overall prevalence of ‘too hot’ by annual household income.

[§] Chi-square test *p*-values indicate significant differences between the overall prevalence of ‘too cold’ by annual household income.

[ǂ] Chi-square or Analysis of Variance (ANOVA) test *p*-values indicate significant differences in characteristics between the total populations of each annual household income category.

[a] Reasons for trouble sleeping ≥ 3 times per week in the past month include: unable to fall asleep within 30 minutes, waking up in the middle of the night or early morning, waking up to use the bathroom, cannot breathe comfortably, coughing or snoring loudly, having bad dreams, having pain, or other non-specified reasons.
[b] Excluded for reporting both ‘Too hot’ and ‘Too cold’, < $20,000 - $49,999: n = 112 (1.7%).
[c] Excluded for reporting both ‘Too hot’ and ‘Too cold’, $50,000 - $99,999: n = 153 (1.7%).
[d] Excluded for reporting both ‘Too hot’ and ‘Too cold’, ≥ $100,000: n = 153 (1.8%).
[e] ‘Infrequent temperature extremes’ is defined as self-reported trouble sleeping due to feeling too hot < 3 times per week and trouble sleeping due to feeling too cold < 3 times per week in the past month.
[f] ‘Too hot’ is defined as self-reported trouble sleeping due to feeling too hot ≥ 3 times per week in the past month.
[g] ‘Too cold’ is defined as self-reported trouble sleeping due to feeling too cold ≥ 3 times per week in the past month.

[h] Educational attainment was assessed at baseline.
[i] Sleep duration is based on reported bed and wake times or reported average sleep duration. Participants who reported ≤ 2 or ≥ 23 hours of sleep are excluded. Short: < 7 hours; Recommended: 7-9 hours; Long ≥ 9 hours.
[j] Long sleep onset latency is defined as not falling asleep within 30 minutes at least three times a week during the past month.
[k] Poor sleep maintenance is defined as waking up in the middle of the night or early morning at least three times a week during the past month.
[l] Insomnia symptoms is defined as long sleep onset latency or poor sleep maintenance.
[m] Sleep medication use is defined as 'taking medicine (prescription or over the counter) to help you sleep' at least three times a week during the past month.
[n] Daytime dysfunction is defined as having trouble staying awake while driving, eating, or engaging in social activity at least three times a week during the past month.
[o] Healthcare provider diagnosed sleep apnea is defined as a current doctor or other health professional diagnosis of sleep apnea.

Supplemental Table 14. Sociodemographic, clinical, and sleep health characteristics among participants who reported trouble sleeping for any reason [a], by region of residence as well as perceived indoor temperature extremes, Sister Study, 2017-2019, N = 23,853

Region	Northeast [b]				Midwest [c]				South [d]				West [e]				Puerto Rico [f]			
	Total n = 3,876 (16.2%)	Infrequent temperature extremes[g] n = 3,334 (86.0%)	Too hot[h ¥] n = 430 (11.1%)	Too cold[i §] n = 55 (1.4%)	Total n = 6,290 (26.4%)	Infrequent temperature extremes[g] n = 5,375 (85.5%)	Too hot[h ¥] n = 702 (11.2%)	Too cold[i §] n = 101 (1.6%)	Total n = 7,916 (33.2%)	Infrequent temperature extremes[g] n = 6,722 (84.9%)	Too hot[h ¥] n = 921 (11.6%)	Too cold[i §] n = 131 (1.7%)	Total n = 5,522 (23.2%)	Infrequent temperature extremes[g] n = 4,725 (85.6%)	Too hot[h ¥] n = 605 (11.0%)	Too cold[i §] n = 96 (1.7%)	Total n = 249 (1.0%)	Infrequent temperature extremes[g] n = 191 (76.7%)	Too hot[h ¥] n = 24 (9.6%)	Too cold[i §] n =23 (9.2%)
**Sociodemographic characteristics**																				
Age, years (mean ± SD)[ł]	67.23 ± 8.58	68.00 ± 8.45	61.77 ± 7.41	68.83 ± 8.31	67.41 ± 8.47	68.16 ± 8.32	62.24 ± 7.48	68.08 ± 9.13	67.61 ± 8.44	68.31 ± 8.31	63.01 ± 7.69	68.64 ± 9.74	68.17 ± 8.35	68.78 ± 8.22	63.71 ± 7.84	70.80 ± 8.15	66.16 ± 8.01	66.44 ± 7.97	64.12 ± 7.63	66.23 ± 8.97
Age,[ł] years																				
< 67.2	1,940 (50.1)	1,538 (46.1)	336 (78.1)	20 (36.4)	3,068 (48.8)	2,414 (44.9)	527 (75.1)	44 (43.6)	3,764 (47.5)	2,963 (44.1)	640 (69.5)	57 (43.5)	2,504 (45.3)	2,010 (42.5)	401 (66.3)	29 (30.2)	135 (54.2)	100 (52.4)	16 (66.7)	14 (60.9)
≥ 67.2	1,936 (49.9)	1,796 (53.9)	94 (21.9)	35 (63.6)	3,222 (51.2)	2,961 (55.1)	175 (24.9)	57 (56.4)	4,152 (52.5)	3,759 (55.9)	281 (30.5)	74 (56.5)	3,018 (54.7)	2,715 (57.5)	204 (33.7)	67 (69.8)	114 (45.8)	91 (47.6)	8 (33.3)	9 (39.1)
Race and ethnicity [ł]																				
Hispanic/Latina	63 (1.6)	45 (1.3)	14 (3.3)	1 (1.8)	47 (0.7)	41 (0.8)	6 (0.9)	0 (0.0)	212 (2.7)	172 (2.6)	28 (3.0)	7 (5.3)	215 (3.9)	176 (3.7)	28 (4.6)	5 (5.2)	246 (98.8)	188 (98.4)	24 (100.0)	23 (100.0)
Non-Hispanic Black	129 (3.3)	102 (3.1)	22 (5.1)	2 (3.6)	284 (4.5)	223 (4.1)	50 (7.1)	6 (5.9)	953 (12.0)	718 (10.7)	189 (20.5)	18 (13.7)	128 (2.3)	107 (2.3)	15 (2.5)	5 (5.2)	0 (0.0)	0 (0.0)	0 (0.0)	0 (0.0)
Non-Hispanic White	3,684 (95.0)	3,187 (95.6)	394 (91.6)	52 (94.5)	5,959 (94.7)	5,111 (95.1)	646 (92.0)	95 (94.1)	6,751 (85.3)	5,832 (86.8)	704 (76.4)	106 (80.9)	5,179 (93.8)	4,442 (94.0)	562 (92.9)	86 (89.6)	3 (1.2)	3 (1.6)	0 (0.0)	0 (0.0)
Educational attainment [ł j]																				
<High school or GED	513 (13.2)	432 (13.0)	59 (13.7)	15 (27.3)	1,046 (16.6)	880 (16.4)	129 (18.4)	23 (22.8)	1,021 (12.9)	866 (12.9)	113 (12.3)	26 (19.8)	572 (10.4)	469 (9.9)	75 (12.4)	11 (11.5)	53 (21.3)	37 (19.4)	8 (33.3)	4 (17.4)
Some college / Technical	1,082 (27.9)	910 (27.3)	133 (30.9)	19 (34.5)	2,125 (33.8)	1,808 (33.6)	233 (33.2)	42 (41.6)	2,526 (31.9)	2,111 (31.4)	320 (34.7)	46 (35.1)	1,803 (32.7)	1,517 (32.1)	220 (36.4)	36 (37.5)	67 (26.9)	48 (25.1)	7 (29.2)	7 (30.4)
≥ Bachelor's or Higher	2,279 (58.8)	1,991 (59.7)	238 (55.3)	21 (38.2)	3,119 (49.6)	2,687 (50.0)	340 (48.4)	36 (35.6)	4,368 (55.2)	3,744 (55.7)	488 (53.0)	59 (45.0)	3,147 (57.0)	2,739 (58.0)	310 (51.2)	49 (51.0)	129 (51.8)	106 (55.5)	9 (37.5)	12 (52.2)
Missing	*2 (0.1)*	*1 (0.0)*	*0 (0.0)*	*0 (0.0)*	*0 (0.0)*	*0 (0.0)*	*0 (0.0)*	*0 (0.0)*	*1 (0.0)*	*1 (0.0)*	*0 (0.0)*	*0 (0.0)*	*0 (0.0)*	*0 (0.0)*	*0 (0.0)*	*0 (0.0)*	*0 (0.0)*	*0 (0.0)*	*0 (0.0)*	*0 (0.0)*
Annual household income [ł]																				
< $20,000 - $49,999	905 (23.3)	797 (23.9)	81 (18.8)	18 (32.7)	1,893 (30.1)	1,667 (31.0)	148 (21.1)	50 (49.5)	2,184 (27.6)	1,882 (28.0)	212 (23.0)	48 (36.6)	1,433 (26.0)	1,247 (26.4)	125 (20.7)	38 (39.6)	199 (79.9)	148 (77.5)	19 (79.2)	22 (95.7)
$50,000 – $99,999	1,343 (34.6)	1,192 (35.8)	113 (26.3)	20 (36.4)	2,475 (39.3)	2,124 (39.5)	276 (39.3)	27 (26.7)	2,916 (36.8)	2,486 (37.0)	336 (36.5)	41 (31.3)	2,046 (37.1)	1,768 (37.4)	215 (35.5)	30 (31.3)	40 (16.1)	35 (18.3)	3 (12.5)	1 (4.3)
≥ $100,000	1,628 (42.0)	1,345 (40.3)	236 (54.9)	17 (30.9)	1,922 (30.6)	1,584 (29.5)	278 (39.6)	24 (23.8)	2,816 (35.6)	2,354 (35.0)	373 (40.5)	42 (32.1)	2,043 (37.0)	1,710 (36.2)	265 (43.8)	28 (29.2)	10 (4.0)	8 (4.2)	2 (8.3)	0 (0.0)
Marital status [ł]																				
Married or living as though married	2,643 (68.2)	2,223 (66.7)	339 (78.8)	39 (70.9)	4,341 (69.0)	3,642 (67.8)	553 (78.8)	65 (64.4)	5,305 (67.0)	4,443 (66.1)	687 (74.6)	80 (61.1)	3,676 (66.6)	3,077 (65.1)	469 (77.5)	56 (58.3)	159 (63.9)	123 (64.4)	17 (70.8)	11 (47.8)
Never married, divorced, widowed, or separated	1,233 (31.8)	1,111 (33.3)	91 (21.2)	16 (29.1)	1,949 (31.0)	1,733 (32.2)	149 (21.2)	36 (35.6)	2,611 (33.0)	2,279 (33.9)	234 (25.4)	51 (38.9)	1,846 (33.4)	1,648 (34.9)	136 (22.5)	40 (41.7)	90 (36.1)	68 (35.6)	7 (29.2)	12 (52.2)

**Clinical characteristics**																				
Body Mass Index, kg/m^2 [‡]																				
Recommended (18.5 - 24.9)	1,571 (40.5)	1,377 (41.3)	146 (34.0)	21 (38.2)	2,152 (34.2)	1,854 (34.5)	221 (31.5)	38 (37.6)	2,882 (36.4)	2,530 (37.6)	260 (28.2)	47 (35.9)	2,416 (43.8)	2,080 (44.0)	240 (39.7)	53 (55.2)	71 (28.5)	50 (26.2)	10 (41.7)	7 (30.4)
Overweight (25.0 - 29.9)	1,216 (31.4)	1,048 (31.4)	138 (32.1)	12 (21.8)	2,068 (32.9)	1,764 (32.8)	237 (33.8)	30 (29.7)	2,548 (32.2)	2,161 (32.1)	309 (33.6)	31 (23.7)	1,698 (30.7)	1,452 (30.7)	203 (33.6)	19 (19.8)	103 (41.4)	82 (42.9)	8 (33.3)	9 (39.1)
Obesity (≥ 30)	1,041 (26.9)	868 (26.0)	141 (32.8)	21 (38.2)	1,998 (31.8)	1,696 (31.6)	233 (33.2)	33 (32.7)	2,372 (30.0)	1,934 (28.8)	343 (37.2)	50 (38.2)	1,340 (24.3)	1,136 (24.0)	155 (25.6)	22 (22.9)	72 (28.9)	57 (29.8)	6 (25.0)	7 (30.4)
Missing	*48 (1.2)*	*41 (1.2)*	*5 (1.2)*	*1 (1.8)*	*72 (1.1)*	*61 (1.1)*	*11 (1.6)*	*0 (0.0)*	*114 (1.4)*	*97 (1.4)*	*9 (1.0)*	*3 (2.3)*	*68 (1.2)*	*57 (1.2)*	*7 (1.2)*	*2 (2.1)*	*3 (1.2)*	*2 (1.0)*	*0 (0.0)*	*0 (0.0)*
Menopausal status																				
Premenopausal	131 (3.4)	98 (2.9)	26 (6.0)	2 (3.6)	210 (3.3)	157 (2.9)	42 (6.0)	6 (5.9)	270 (3.4)	197 (2.9)	56 (6.1)	8 (6.1)	166 (3.0)	124 (2.6)	33 (5.5)	3 (3.1)	14 (5.6)	12 (6.3)	1 (4.2)	1 (4.3)
Postmenopausal	3,745 (96.6)	3,236 (97.1)	404 (94.0)	53 (96.4)	6,080 (96.7)	5,218 (97.1)	660 (94.0)	95 (94.1)	7,642 (96.5)	6,521 (97.0)	865 (93.9)	123 (93.9)	5,355 (97.0)	4,600 (97.4)	572 (94.5)	93 (96.9)	235 (94.4)	179 (93.7)	23 (95.8)	22 (95.7)
Missing or never had a period	*0 (0.0)*	*0 (0.0)*	*0 (0.0)*	*0 (0.0)*	*0 (0.0)*	*0 (0.0)*	*0 (0.0)*	*0 (0.0)*	*4 (0.1)*	*4 (0.1)*	*0 (0.0)*	*0 (0.0)*	*1 (0.0)*	*1 (0.0)*	*0 (0.0)*	*0 (0.0)*	*0 (0.0)*	*0 (0.0)*	*0 (0.0)*	*0 (0.0)*
**Sleep health dimensions**																				
Average weekly sleep duration [‡ k]																				
Short	522 (13.5)	442 (13.3)	58 (13.5)	15 (27.3)	704 (11.2)	564 (10.5)	108 (15.4)	17 (16.8)	1,004 (12.7)	824 (12.3)	129 (14.0)	22 (16.8)	572 (10.4)	486 (10.3)	68 (11.2)	11 (11.5)	42 (16.9)	32 (16.8)	6 (25.0)	2 (8.7)
Recommended	2,715 (70.0)	2,344 (70.3)	303 (70.5)	29 (52.7)	4,539 (72.2)	3,930 (73.1)	477 (67.9)	66 (65.3)	5,502 (69.5)	4,719 (70.2)	621 (67.4)	77 (58.8)	3,986 (72.2)	3,446 (72.9)	416 (68.8)	55 (57.3)	156 (62.7)	128 (67.0)	8 (33.3)	15 (65.2)
Long	584 (15.1)	498 (14.9)	66 (15.3)	9 (16.4)	972 (15.5)	815 (15.2)	112 (16.0)	15 (14.9)	1,316 (16.6)	1,104 (16.4)	160 (17.4)	27 (20.6)	908 (16.4)	747 (15.8)	116 (19.2)	28 (29.2)	39 (15.7)	24 (12.6)	7 (29.2)	4 (17.4)
Missing	*55 (1.4)*	*50 (1.5)*	*3 (0.7)*	*2 (3.6)*	*75 (1.2)*	*66 (1.2)*	*5 (0.7)*	*3 (3.0)*	*94 (1.2)*	*75 (1.1)*	*11 (1.2)*	*5 (3.8)*	*56 (1.0)*	*46 (1.0)*	*5 (0.8)*	*2 (2.1)*	*12 (4.8)*	*7 (3.7)*	*3 (12.5)*	*2 (8.7)*
Long sleep onset latency [‡ l]																				
Yes	634 (16.4)	499 (15.0)	102 (23.7)	13 (23.6)	1,064 (16.9)	822 (15.3)	161 (22.9)	34 (33.7)	1,501 (19.0)	1,126 (16.8)	278 (30.2)	34 (26.0)	910 (16.5)	700 (14.8)	141 (23.3)	28 (29.2)	74 (29.7)	50 (26.2)	7 (29.2)	11 (47.8)
No	3,211 (82.8)	2,809 (84.3)	324 (75.3)	41 (74.5)	5,177 (82.3)	4,511 (83.9)	537 (76.5)	66 (65.3)	6,344 (80.1)	5,535 (82.3)	638 (69.3)	95 (72.5)	4,571 (82.8)	3,989 (84.4)	461 (76.2)	67 (69.8)	159 (63.9)	133 (69.6)	13 (54.2)	9 (39.1)
Missing	*31 (0.8)*	*26 (0.8)*	*4 (0.9)*	*1 (1.8)*	*49 (0.8)*	*42 (0.8)*	*4 (0.6)*	*1 (1.0)*	*71 (0.9)*	*61 (0.9)*	*5 (0.5)*	*2 (1.5)*	*41 (0.7)*	*36 (0.8)*	*3 (0.5)*	*1 (1.0)*	*16 (6.4)*	*8 (4.2)*	*4 (16.7)*	*3 (13.0)*
Poor sleep maintenance [‡ m]																				
Yes	2,228 (57.5)	1,808 (54.2)	339 (78.8)	33 (60.0)	3,437 (54.6)	2,781 (51.7)	501 (71.4)	70 (69.3)	4,336 (54.8)	3,477 (51.7)	661 (71.8)	85 (64.9)	3,141 (56.9)	2,565 (54.3)	447 (73.9)	59 (61.5)	118 (47.4)	81 (42.4)	16 (66.7)	15 (65.2)
No	1,620 (41.8)	1,499 (45.0)	90 (20.9)	22 (40.0)	2,809 (44.7)	2,557 (47.6)	196 (27.9)	31 (30.7)	3,515 (44.4)	3,184 (47.4)	257 (27.9)	46 (35.1)	2,341 (42.4)	2,124 (45.0)	155 (25.6)	37 (38.5)	118 (47.4)	101 (52.9)	7 (29.2)	6 (26.1)
Missing	*28 (0.7)*	*27 (0.8)*	*1 (0.2)*	*0 (0.0)*	*44 (0.7)*	*37 (0.7)*	*5 (0.7)*	*0 (0.0)*	*65 (0.8)*	*61 (0.9)*	*3 (0.3)*	*0 (0.0)*	*40 (0.7)*	*36 (0.8)*	*3 (0.5)*	*0 (0.0)*	*13 (5.2)*	*9 (4.7)*	*1 (4.2)*	*2 (8.7)*
Insomnia symptoms [‡ n]																				
Yes	2,380 (61.4)	1,939 (58.2)	356 (82.8)	34 (61.8)	3,739 (59.4)	3,035 (56.5)	536 (76.4)	74 (73.3)	4,742 (59.9)	3,814 (56.7)	712 (77.3)	92 (70.2)	3,357 (60.8)	2,737 (57.9)	480 (79.3)	62 (64.6)	137 (55.0)	96 (50.3)	18 (75.0)	17 (73.9)
No	1,489 (38.4)	1,388 (41.6)	74 (17.2)	21 (38.2)	2,536 (40.3)	2,328 (43.3)	164 (23.4)	27 (26.7)	3,154 (39.8)	2,889 (43.0)	209 (22.7)	39 (29.8)	2,151 (39.0)	1,974 (41.8)	125 (20.7)	34 (35.4)	101 (40.6)	88 (46.1)	5 (20.8)	4 (17.4)
Missing	*7 (0.2)*	*7 (0.2)*	*0 (0.0)*	*0 (0.0)*	*15 (0.2)*	*12 (0.2)*	*2 (0.3)*	*0 (0.0)*	*20 (0.3)*	*19 (0.3)*	*0 (0.0)*	*0 (0.0)*	*14 (0.3)*	*14 (0.3)*	*0 (0.0)*	*0 (0.0)*	*11 (4.4)*	*7 (3.7)*	*1 (4.2)*	*2 (8.7)*

Sleep medication use [ł, o]																				
Yes	732 (18.9)	608 (18.2)	94 (21.9)	18 (32.7)	1,297 (20.6)	1,085 (20.2)	148 (21.1)	31 (30.7)	1,744 (22.0)	1,435 (21.3)	231 (25.1)	33 (25.2)	1,239 (22.4)	1,047 (22.2)	143 (23.6)	22 (22.9)	65 (26.1)	44 (23.0)	7 (29.2)	10 (43.5)
No	3,129 (80.7)	2,715 (81.4)	333 (77.4)	37 (67.3)	4,968 (79.0)	4,268 (79.4)	551 (78.5)	70 (69.3)	6,139 (77.6)	5,262 (78.3)	685 (74.4)	96 (73.3)	4,261 (77.2)	3,659 (77.4)	461 (76.2)	73 (76.0)	177 (71.1)	143 (74.9)	17 (70.8)	12 (52.2)
Missing	*15 (0.4)*	*11 (0.3)*	*3 (0.7)*	*0 (0.0)*	*25 (0.4)*	*22 (0.4)*	*3 (0.4)*	*0 (0.0)*	*33 (0.4)*	*25 (0.4)*	*5 (0.5)*	*2 (1.5)*	*22 (0.4)*	*19 (0.4)*	*1 (0.2)*	*1 (1.0)*	*7 (2.8)*	*4 (2.1)*	*0 (0.0)*	*1 (4.3)*
Daytime dysfunction [ł, p]																				
Yes	31 (0.8)	24 (0.7)	5 (1.2)	2 (3.6)	53 (0.8)	43 (0.8)	7 (1.0)	1 (1.0)	87 (1.1)	55 (0.8)	16 (1.7)	8 (6.1)	46 (0.8)	33 (0.7)	7 (1.2)	4 (4.2)	2 (0.8)	0 (0.0)	1 (4.2)	1 (4.3)
No	3,801 (98.1)	3,270 (98.1)	421 (97.9)	53 (96.4)	6,160 (97.9)	5,268 (98.0)	684 (97.4)	99 (98.0)	7,744 (97.8)	6,596 (98.1)	893 (97.0)	122 (93.1)	5,414 (98.0)	4,636 (98.1)	595 (98.3)	90 (93.8)	237 (95.2)	183 (95.8)	22 (91.7)	22 (95.7)
Missing	*44 (1.1)*	*40 (1.2)*	*4 (0.9)*	*0 (0.0)*	*77 (1.2)*	*64 (1.2)*	*11 (1.6)*	*1 (1.0)*	*85 (1.1)*	*71 (1.1)*	*12 (1.3)*	*1 (0.8)*	*62 (1.1)*	*56 (1.2)*	*3 (0.5)*	*2 (2.1)*	*10 (4.0)*	*8 (4.2)*	*1 (4.2)*	*0 (0.0)*
Healthcare provider diagnosed sleep apnea [ł, q]																				
Yes	353 (9.1)	294 (8.8)	47 (10.9)	9 (16.4)	662 (10.5)	560 (10.4)	77 (11.0)	9 (8.9)	890 (11.2)	709 (10.5)	132 (14.3)	30 (22.9)	644 (11.7)	547 (11.6)	66 (10.9)	18 (18.8)	15 (6.0)	7 (3.7)	3 (12.5)	5 (21.7)
No	3,478 (89.7)	3,000 (90.0)	379 (88.1)	45 (81.8)	5,552 (88.3)	4,754 (88.4)	618 (88.0)	87 (86.1)	6,939 (87.7)	5,935 (88.3)	785 (85.2)	97 (74.0)	4,827 (87.4)	4,134 (87.5)	535 (88.4)	77 (80.2)	227 (91.2)	179 (93.7)	21 (87.5)	18 (78.3)
Missing	*45 (1.2)*	*40 (1.2)*	*4 (0.9)*	*1 (1.8)*	*76 (1.2)*	*61 (1.1)*	*7 (1.0)*	*5 (5.0)*	*87 (1.1)*	*78 (1.2)*	*4 (0.4)*	*4 (3.1)*	*51 (0.9)*	*44 (0.9)*	*4 (0.7)*	*1 (1.0)*	*7 (2.8)*	*5 (2.6)*	*0 (0.0)*	*0 (0.0)*

Abbreviations: SD, Standard Deviation; GED: General Educational Development

Note: N = 418 (1.8%) participants who reported both 'too hot' and 'too cold' temperatures were not included in this sensitivity analysis.

[¥] Chi-square test *p*-values indicate significant differences between the overall prevalence of 'too hot' by region of residence.

[§] Chi-square test *p*-values indicate significant differences between the overall prevalence of 'too cold' by region of residence.

[ł] Chi-square or Analysis of Variance (ANOVA) test *p*-values indicate significant differences in characteristics between the total populations of each region of residence.

[a] Reasons for trouble sleeping ≥ 3 times per week in the past month include: unable to fall asleep within 30 minutes, waking up in the middle of the night or early morning, waking up to use the bathroom, cannot breathe comfortably, coughing or snoring loudly, having bad dreams, having pain, or other non-specified reasons.

[b] Excluded for reporting both 'Too hot' and 'Too cold', Northeast: n = 57 (1.5%).

[c] Excluded for reporting both 'Too hot' and 'Too cold', Midwest: n = 112 (1.8%).

[d] Excluded for reporting both 'Too hot' and 'Too cold', South: n = 142 (1.8%).

[e] Excluded for reporting both 'Too hot' and 'Too cold', West: n = 96 (1.7%).

[f] Excluded for reporting both 'Too hot' and 'Too cold', Puerto Rico: n = 11 (4.4%).

[g] 'Infrequent temperature extremes' is defined as self-reported trouble sleeping due to feeling too hot < 3 times per week and trouble sleeping due to feeling too cold < 3 times per week in the past month.

[h] 'Too hot' is defined as self-reported trouble sleeping due to feeling too hot ≥ 3 times per week in the past month.

[i] 'Too cold' is defined as self-reported trouble sleeping due to feeling too cold ≥ 3 times per week in the past month.

[j] Educational attainment was assessed at baseline.

[k] Sleep duration is based on reported bed and wake times or reported average sleep duration. Participants who reported ≤ 2 or ≥ 23 hours of sleep are excluded. Short: < 7 hours; Recommended: 7-9 hours; Long ≥ 9 hours.

[l] Long sleep onset latency is defined as not falling asleep within 30 minutes at least three times a week during the past month.

[m] Poor sleep maintenance is defined as waking up in the middle of the night or early morning at least three times a week during the past month.

[n] Insomnia symptoms is defined as long sleep onset latency or poor sleep maintenance.

[o] Sleep medication use is defined as 'taking medicine (prescription or over the counter) to help you sleep' at least three times a week during the past month.

[p] Daytime dysfunction is defined as having trouble staying awake while driving, eating, or engaging in social activity at least three times a week during the past month.

[q] Healthcare provider diagnosed sleep apnea is defined as a current doctor or other health professional diagnosis of sleep apnea.

Supplemental Table 15. Sociodemographic, clinical, and sleep health characteristics among participants who reported trouble sleeping for any reason [a], by menopausal status as well as perceived indoor temperature extremes, Sister Study, 2017-2019, N = 23,853

**Menopausal status**	**Premenopausal [b]**				**Postmenopausal [c]**			
	Total n = 791 (3.3%)	Infrequent temperature extremes [d] n = 588 (74.3%)	Too hot [e ¥] n = 158 (20.0%)	Too cold [f §] n = 20 (2.5%)	Total n = 23,057 (96.7%)	Infrequent temperature extremes [d] n = 19,754 (85.7%)	Too hot [e ¥] n = 2,524 (10.9%)	Too cold [f §] n = 386 (1.7%)
**Sociodemographic characteristics**								
Age, years (mean ± SD) [ǂ]	51.34 ± 2.60	51.21 ± 2.60	51.74 ± 2.58	51.33 ± 2.79	68.16 ± 8.02	68.82 ± 7.88	63.47 ± 7.33	69.81 ± 8.28
Age, years (n (%)) [ǂ]								
< 67.2	791 (100.0)	588 (100.0)	158 (100.0)	20 (100.0)	10,620 (46.1)	8,437 (42.7)	1,762 (69.8)	144 (37.3)
≥ 67.2	0 (0.0)	0 (0.0)	0 (0.0)	0 (0.0)	12,437 (53.9)	11,317 (57.3)	762 (30.2)	242 (62.7)
Race and ethnicity [ǂ]								
Hispanic/Latina	50 (6.3)	42 (7.1)	7 (4.4)	1 (5.0)	733 (3.2)	580 (2.9)	93 (3.7)	35 (9.1)
Non-Hispanic Black	85 (10.7)	53 (9.0)	24 (15.2)	5 (25.0)	1,407 (6.1)	1,095 (5.5)	252 (10.0)	26 (6.7)
Non-Hispanic White	656 (82.9)	493 (83.8)	127 (80.4)	14 (70.0)	20,917 (90.7)	18,079 (91.5)	2,179 (86.3)	325 (84.2)
Educational attainment [ǂ g]								
<High school or GED	70 (8.8)	48 (8.2)	15 (9.5)	5 (25.0)	3,134 (13.6)	2,635 (13.3)	369 (14.6)	74 (19.2)
Some college /technical	222 (28.1)	155 (26.4)	48 (30.4)	8 (40.0)	7,379 (32.0)	6,237 (31.6)	865 (34.3)	142 (36.8)
≥ Bachelor's or higher	499 (63.1)	385 (65.5)	95 (60.1)	7 (35.0)	12,541 (54.4)	10,880 (55.1)	1,290 (51.1)	170 (44.0)
Missing	*0 (0.0)*	*0 (0.0)*	*0 (0.0)*	*0 (0.0)*	*3 (0.0)*	*2 (0.0)*	*0 (0.0)*	*0 (0.0)*
Annual household income [ǂ]								
< $20,000 - $49,999	91 (11.5)	67 (11.4)	18 (11.4)	1 (5.0)	6,520 (28.3)	5,671 (28.7)	567 (22.5)	175 (45.3)
$50,000 – $99,999	209 (26.4)	144 (24.5)	48 (30.4)	9 (45.0)	8,610 (37.3)	7,460 (37.8)	895 (35.5)	110 (28.5)
≥ $100,000	491 (62.1)	377 (64.1)	92 (58.2)	10 (50.0)	7,927 (34.4)	6,623 (33.5)	1,062 (42.1)	101 (26.2)
Marital status [ǂ]								
Married or living as though married	598 (75.6)	445 (75.7)	122 (77.2)	16 (80.0)	15,524 (67.3)	13,061 (66.1)	1,943 (77.0)	235 (60.9)
Never married, divorced, widowed, or separated	193 (24.4)	143 (24.3)	36 (22.8)	4 (20.0)	7,533 (32.7)	6,693 (33.9)	581 (23.0)	151 (39.1)
Region of residence								
Northeast	131 (16.6)	98 (16.7)	26 (16.5)	2 (10.0)	3,745 (16.2)	3,236 (16.4)	404 (16.0)	53 (13.7)
Midwest	210 (26.5)	157 (26.7)	42 (26.6)	6 (30.0)	6,080 (26.4)	5,218 (26.4)	660 (26.1)	95 (24.6)
South	270 (34.1)	197 (33.5)	56 (35.4)	8 (40.0)	7,642 (33.1)	6,521 (33.0)	865 (34.3)	123 (31.9)
West	166 (21.0)	124 (21.1)	33 (20.9)	3 (15.0)	5,355 (23.2)	4,600 (23.3)	572 (22.7)	93 (24.1)
Puerto Rico	14 (1.8)	12 (2.0)	1 (0.6)	1 (5.0)	235 (1.0)	179 (0.9)	23 (0.9)	22 (5.7)
**Clinical characteristics**								
Body Mass Index, kg/m^2 [ǂ]								
Recommended (18.5 - 24.9)	271 (34.3)	208 (35.4)	44 (27.8)	7 (35.0)	8,819 (38.2)	7,681 (38.9)	833 (33.0)	159 (41.2)
Overweight (25.0 - 29.9)	229 (29.0)	171 (29.1)	47 (29.7)	4 (20.0)	7,403 (32.1)	6,335 (32.1)	848 (33.6)	97 (25.1)
Obesity (≥ 30)	282 (35.7)	204 (34.7)	63 (39.9)	9 (45.0)	6,539 (28.4)	5,485 (27.8)	815 (32.3)	124 (32.1)
Missing	*9 (1.1)*	*5 (0.9)*	*4 (2.5)*	*0 (0.0)*	*296 (1.3)*	*253 (1.3)*	*28 (1.1)*	*6 (1.6)*
**Sleep health dimensions**								

Average weekly sleep duration [ǂ h]								
Short	112 (14.2)	79 (13.4)	26 (16.5)	5 (25.0)	2,732 (11.8)	2,269 (11.5)	343 (13.6)	62 (16.1)
Recommended	573 (72.4)	435 (74.0)	108 (68.4)	12 (60.0)	16,321 (70.8)	14,128 (71.5)	1,717 (68.0)	230 (59.6)
Long	101 (12.8)	71 (12.1)	23 (14.6)	3 (15.0)	3,717 (16.1)	3,116 (15.8)	438 (17.4)	80 (20.7)
Missing	*5 (0.6)*	*3 (0.5)*	*1 (0.6)*	*0 (0.0)*	*287 (1.2)*	*241 (1.2)*	*26 (1.0)*	*14 (3.6)*
Long sleep onset latency [i]								
Yes	125 (15.8)	76 (12.9)	36 (22.8)	2 (10.0)	4,057 (17.6)	3,120 (15.8)	653 (25.9)	118 (30.6)
No	659 (83.3)	508 (86.4)	120 (75.9)	18 (90.0)	18,799 (81.5)	16,465 (83.4)	1,853 (73.4)	260 (67.4)
Missing	*7 (0.9)*	*4 (0.7)*	*2 (1.3)*	*0 (0.0)*	*201 (0.9)*	*169 (0.9)*	*18 (0.7)*	*8 (2.1)*
Poor sleep maintenance [ǂ j]								
Yes	478 (60.4)	330 (56.1)	117 (74.1)	14 (70.0)	12,779 (55.4)	10,379 (52.5)	1,847 (73.2)	248 (64.2)
No	308 (38.9)	256 (43.5)	38 (24.1)	6 (30.0)	10,093 (43.8)	9,207 (46.6)	667 (26.4)	136 (35.2)
Missing	*5 (0.6)*	*2 (0.3)*	*3 (1.9)*	*0 (0.0)*	*185 (0.8)*	*168 (0.9)*	*10 (0.4)*	*2 (0.5)*
Insomnia symptoms [ǂ k]								
Yes	512 (64.7)	354 (60.2)	125 (79.1)	14 (70.0)	13,840 (60.0)	11,264 (57.0)	1,977 (78.3)	265 (68.7)
No	277 (35.0)	232 (39.5)	33 (20.9)	6 (30.0)	9,152 (39.7)	8,433 (42.7)	544 (21.6)	119 (30.8)
Missing	*2 (0.3)*	*2 (0.3)*	*0 (0.0)*	*0 (0.0)*	*65 (0.3)*	*57 (0.3)*	*3 (0.1)*	*2 (0.5)*
Sleep medication use [ǂ l]								
Yes	138 (17.4)	100 (17.0)	28 (17.7)	5 (25.0)	4,939 (21.4)	4,119 (20.9)	595 (23.6)	109 (28.2)
No	650 (82.2)	485 (82.5)	130 (82.3)	15 (75.0)	18,019 (78.1)	15,557 (78.8)	1,917 (76.0)	273 (70.7)
Missing	*3 (0.4)*	*3 (0.5)*	*0 (0.0)*	*0 (0.0)*	*99 (0.4)*	*78 (0.4)*	*12 (0.5)*	*4 (1.0)*
Daytime dysfunction [m]								
Yes	7 (0.9)	4 (0.7)	1 (0.6)	2 (10.0)	211 (0.9)	150 (0.8)	35 (1.4)	14 (3.6)
No	777 (98.2)	579 (98.5)	155 (98.1)	18 (90.0)	22,575 (97.9)	19,370 (98.1)	2,460 (97.5)	368 (95.3)
Missing	*7 (0.9)*	*5 (0.9)*	*2 (1.3)*	*0 (0.0)*	*271 (1.2)*	*234 (1.2)*	*29 (1.1)*	*4 (1.0)*
Healthcare provider diagnosed sleep apnea [ǂ n]								
Yes	60 (7.6)	45 (7.7)	11 (7.0)	1 (5.0)	2,503 (10.9)	2,071 (10.5)	314 (12.4)	70 (18.1)
No	726 (91.8)	538 (91.5)	147 (93.0)	19 (95.0)	20,293 (88.0)	17,460 (88.4)	2,191 (86.8)	305 (79.0)
Missing	*5 (0.6)*	*5 (0.9)*	*0 (0.0)*	*0 (0.0)*	*261 (1.1)*	*223 (1.1)*	*19 (0.8)*	*11 (2.8)*

Abbreviations: SD, Standard Deviation; GED: General Educational Development

Note: N = 418 (1.8%) participants who reported both 'too hot' and 'too cold' temperatures were not included in this sensitivity analysis.

[¥] Chi-square test *p*-values indicate significant differences between the overall prevalence of 'too hot' by menopausal status.

[§] Chi-square test *p*-values indicate significant differences between the overall prevalence of 'too cold' by menopausal status.

[ǂ] Chi-square or Analysis of Variance (ANOVA) test *p*-values indicate significant differences in characteristics between the total populations of each menopausal status category.

[a] Reasons for trouble sleeping ≥ 3 times per week in the past month include: unable to fall asleep within 30 minutes, waking up in the middle of the night or early morning, waking up to use the bathroom, cannot breathe comfortably, coughing or snoring loudly, having bad dreams, having pain, or other non-specified reasons.

[b] Excluded for reporting both 'Too hot' and 'Too cold', Premenopausal: n = 25 (3.2%).

[c] Excluded for reporting both 'Too hot' and 'Too cold', Postmenopausal: n = 393 (1.7%).

[d] 'Infrequent temperature extremes' is defined as self-reported trouble sleeping due to feeling too hot < 3 times per week and trouble sleeping due to feeling too cold < 3 times per week in the past month.

[e] 'Too hot' is defined as self-reported trouble sleeping due to feeling too hot ≥ 3 times per week in the past month.

[f] 'Too cold' is defined as self-reported trouble sleeping due to feeling too cold ≥ 3 times per week in the past month.

[g] Educational attainment was assessed at baseline.

[h] Sleep duration is based on reported bed and wake times or reported average sleep duration. Participants who reported ≤ 2 or ≥ 23 hours of sleep are excluded. Short: < 7 hours; Recommended: 7-9 hours; Long ≥ 9 hours.

[i] Long sleep onset latency is defined as not falling asleep within 30 minutes at least three times a week during the past month.

[j] Poor sleep maintenance is defined as waking up in the middle of the night or early morning at least three times a week during the past month.
[k] Insomnia symptoms is defined as long sleep onset latency or poor sleep maintenance.
[l] Sleep medication use is defined as 'taking medicine (prescription or over the counter) to help you sleep' at least three times a week during the past month.
[m] Daytime dysfunction is defined as having trouble staying awake while driving, eating, or engaging in social activity at least three times a week during the past month.
[n] Healthcare provider diagnosed sleep apnea is defined as a current doctor or other health professional diagnosis of sleep apnea.

Supplemental Table 16. Prevalence ratios (95% confidence intervals) for associations between perceived indoor temperature extremes and sleep health dimensions among participants who reported trouble sleeping for any reason [a], Sister Study, 2017-2019

	Indoor temperature: Too hot [b]	Indoor temperature: Too cold [c]
Weekly sleep duration [d]		
Short	**1.14 (1.03,1.27)‡**	**1.43 (1.16,1.76)‡**
Recommended	1.00 (ref)	1.00 (ref)
Long	**1.23 (1.12,1.35)‡**	**1.36 (1.13,1.65)‡**
Long sleep onset latency [e]		
Yes	**1.80 (1.66,1.96)‡**	**1.87 (1.58,2.21)‡**
No	1.00 (ref)	1.00 (ref)
Poor sleep maintenance [f]		
Yes	**1.43 (1.38,1.47)‡**	**1.26 (1.16,1.36)‡**
No	1.00 (ref)	1.00 (ref)
Insomnia symptoms [g]		
Yes	**1.42 (1.38,1.46)‡**	**1.24 (1.15,1.34)‡**
No	1.00 (ref)	1.00 (ref)
Sleep medication use [h]		
Yes	**1.18 (1.09,1.27)‡**	**1.34 (1.14,1.57)‡**
No	1.00 (ref)	1.00 (ref)
Daytime dysfunction [i]		
Yes	**1.75 (1.21,2.54)‡**	**4.86 (2.91,8.09)‡**
No	1.00 (ref)	1.00 (ref)
Healthcare professional diagnosed sleep apnea [j]		
Yes	**1.16 (1.04,1.29)‡**	**1.65 (1.35,2.03)‡**
No	1.00 (ref)	1.00 (ref)

Note: N = 418 (1.8%) participants who reported both 'too hot' and 'too cold' temperatures were not included in this sensitivity analysis.

Models are adjusted for age (continuous), age^2, race and ethnicity (Hispanic/Latina, non-Hispanic Black, non-Hispanic White), annual household income (<$20,000 - $49,999, $50,000 - $99,999, ≥$100,000), marital status (married or living as though married, divorced/widowed/separated/never married), region of residence (Northeast, Midwest, South, West, and Puerto Rico), body mass index (BMI: underweight, recommended, overweight, obesity), and menopausal status (premenopausal, postmenopausal).

[a] In models for weekly sleep duration, sleep medication use, daytime dysfunction, and healthcare professional diagnosed sleep apnea, reasons for trouble sleeping for any reason other than feeling too hot or feeling too cold ≥ 3 times per week in the past month include: unable to fall asleep within 30 minutes, waking up in the middle of the night or early morning, waking up to use the bathroom, cannot breathe comfortably, coughing or snoring loudly, having bad dreams, having pain, or other non-specified reasons (n = 23,853). In models for long sleep onset latency, poor sleep maintenance, and insomnia symptoms, reasons for trouble sleeping for any reason other than feeling too hot or feeling too cold ≥ 3 times per week in the past month include waking up to use the bathroom, cannot breathe comfortably, coughing or snoring loudly, having bad dreams, having pain, or other non-specified reasons (n = 21,641).
[b] 'Too hot' is defined as self-reported trouble sleeping due to feeling too hot ≥ 3 times per week vs. trouble sleeping due to feeling too hot < 3 times per week and trouble sleeping due to feeling too cold < 3 times per week.

[c] 'Too cold' is defined as self-reported trouble sleeping due to feeling too cold ≥ 3 times per week vs. trouble sleeping due to feeling too hot < 3 times per week and trouble sleeping due to feeling too cold < 3 times per week.
[d] Sleep duration is based on reported bed and wake times or reported average sleep duration. Participants who reported ≤ 2 or ≥ 23 hours of sleep are excluded. Short: <7 hours; Recommended: 7-9 hours; Long ≥ 9 hours.
[e] Long sleep onset latency is defined as not falling asleep within 30 minutes at least three times a week during the past month.
[f] Poor sleep maintenance is defined as waking up in the middle of the night or early morning at least three times a week during the past month.
[g] Insomnia symptoms is defined as long sleep onset latency or poor sleep maintenance.
[h] Sleep medication use is defined as 'taking medicine (prescription or over the counter) to help you sleep' at least three times a week during the past month.
[i] Daytime dysfunction is defined as having trouble staying awake while driving, eating, or engaging in social activity at least three times a week during the past month.
[j] Healthcare provider diagnosed sleep apnea is defined as a current doctor or other health professional diagnosis of sleep apnea.
[ŧ] Remained significant after false discovery rate correction. False discovery rate-corrected *p*-value was considered statistically significant at the $\alpha = 0.05$ level.

Supplemental Table 17A. Prevalence ratios (95% confidence intervals) for associations between perceived indoor temperature extremes and sleep health dimensions by race and ethnicity, among participants who reported trouble sleeping for any reason [a], Sister Study, 2017-2019

	Indoor temperature: Too hot [b]				Indoor temperature: Too cold [c]			
	Hispanic/Latina	Non-Hispanic Black	Non-Hispanic White	Wald *p*-values	Hispanic/Latina	Non-Hispanic Black	Non-Hispanic White	Wald *p*-values
Weekly sleep duration [d]								
Short	1.15 (0.73,1.82)	1.06 (0.87,1.29)	**1.17 (1.03,1.32)‡**	0.7029	0.73 (0.28,1.88)	**1.54 (1.07,2.19)‡**	**1.51 (1.18,1.94)‡**	0.3298
Recommended	1.00 (Ref)	1.00 (Ref)	1.00 (Ref)	---	1.00 (Ref)	1.00 (Ref)	1.00 (Ref)	---
Long	1.42 (0.93,2.16)	0.98 (0.70,1.38)	**1.24 (1.13,1.37)‡**	0.3402	1.15 (0.56,2.38)	1.46 (0.62,3.41)	**1.38 (1.13,1.69)‡**	0.8859
Long sleep onset latency [e]								
Yes	**2.03 (1.51,2.72)‡**	**2.02 (1.61,2.52)‡**	**1.76 (1.61,1.93)‡**	0.3987	1.50 (0.92,2.45)	1.13 (0.51,2.51)	**1.99 (1.66,2.39)‡**	0.2492
No	1.00 (Ref)	1.00 (Ref)	1.00 (Ref)	---	1.00 (Ref)	1.00 (Ref)	1.00 (Ref)	---
Poor sleep maintenance [f]								
Yes	**1.45 (1.22,1.72)‡**	**1.36 (1.21,1.53)‡**	**1.43 (1.39,1.48)‡**	0.6953	**1.46 (1.10,1.94)‡**	**1.37 (1.03,1.82)‡**	**1.23 (1.13,1.35)‡**	0.4502
No	1.00 (Ref)	1.00 (Ref)	1.00 (Ref)	---	1.00 (Ref)	1.00 (Ref)	1.00 (Ref)	---
Insomnia symptoms [g]								
Yes	**1.50 (1.30,1.74)‡**	**1.39 (1.25,1.55)‡**	**1.42 (1.38,1.46)‡**	0.6751	**1.38 (1.06,1.79)‡**	**1.38 (1.06,1.80)‡**	**1.22 (1.12,1.32)‡**	0.4668
No	1.00 (Ref)	1.00 (Ref)	1.00 (Ref)	---	1.00 (Ref)	1.00 (Ref)	1.00 (Ref)	---
Sleep medication use [h]								
Yes	1.06 (0.68,1.67)	1.15 (0.83,1.58)	**1.19 (1.10,1.28)‡**	0.8813	1.37 (0.82,2.30)	1.03 (0.41,2.61)	**1.35 (1.14,1.60)‡**	0.8529
No	1.00 (Ref)	1.00 (Ref)	1.00 (Ref)	---	1.00 (Ref)	1.00 (Ref)	1.00 (Ref)	---
Daytime dysfunction [i]								
Yes	0.69 (0.08,5.95)	2.01 (0.60,6.69)	**1.81 (1.22,2.68)‡**	0.6754	**6.40 (1.56,26.28)‡**	**12.60 (3.37,47.10)‡**	**4.05 (2.19,7.51)‡**	0.2910
No	1.00 (Ref)	1.00 (Ref)	1.00 (Ref)	---	1.00 (Ref)	1.00 (Ref)	1.00 (Ref)	---
Healthcare professional diagnosed sleep apnea [j]								
Yes	1.39 (0.79,2.44)	1.27 (0.96,1.67)	**1.13 (1.00,1.28)‡**	0.6088	**2.71 (1.39,5.27)‡**	**2.11 (1.19,3.75)‡**	**1.53 (1.21,1.93)‡**	0.2008
No	1.00 (Ref)	1.00 (Ref)	1.00 (Ref)	---	1.00 (Ref)	1.00 (Ref)	1.00 (Ref)	---

Note: N = 418 (1.8%) participants who reported both 'too hot' and 'too cold' temperatures were not included in this sensitivity analysis.

Models are adjusted for age (continuous), age^2, annual household income (<$20,000 - $49,999, $50,000 - $99,999, ≥$100,000), marital status (married or living as though married, divorced/widowed/separated/never married), region of residence (Northeast, Midwest, South, West, and Puerto Rico), body mass index (BMI: underweight, recommended, overweight, obesity), and menopausal status (premenopausal, postmenopausal).

[a] In models for weekly sleep duration, sleep medication use, daytime dysfunction, and healthcare professional diagnosed sleep apnea, reasons for trouble sleeping for any reason other than feeling too hot or feeling too cold ≥ 3 times per week in the past month include: unable to fall asleep within 30 minutes, waking up in the middle of the night or early morning, waking up to use the bathroom, cannot breathe comfortably, coughing or snoring loudly, having bad dreams, having pain, or other non-specified reasons (n = 23,853). In models for long sleep onset latency, poor sleep maintenance, and insomnia symptoms, reasons for trouble sleeping for any reason other than feeling too hot or feeling too cold ≥ 3 times per week in the past month include waking up to use the bathroom, cannot breathe comfortably, coughing or snoring loudly, having bad dreams, having pain, or other non-specified reasons (n = 21,641).
[b] Too hot' is defined as self-reported trouble sleeping due to feeling too hot ≥ 3 times per week vs. trouble sleeping due to feeling too hot < 3 times per week and trouble sleeping due to feeling too cold < 3 times per week.
[c] 'Too cold' is defined as self-reported trouble sleeping due to feeling too cold ≥ 3 times per week vs. trouble sleeping due to feeling too hot < 3 times per week and trouble sleeping due to feeling too cold < 3 times per week.
[d] Sleep duration is based on reported bed and wake times or reported average sleep duration. Participants who reported ≤ 2 or ≥ 23 hours of sleep are excluded. Short: <7 hours; Recommended: 7-9 hours; Long ≥ 9 hours.
[e] Long sleep onset latency is defined as not falling asleep within 30 minutes at least three times a week during the past month.
[f] Poor sleep maintenance is defined as waking up in the middle of the night or early morning at least three times a week during the past month.
[g] Insomnia symptoms is defined as long sleep onset latency or poor sleep maintenance.
[h] Sleep medication use is defined as 'taking medicine (prescription or over the counter) to help you sleep' at least three times a week during the past month.

[i] Daytime dysfunction is defined as having trouble staying awake while driving, eating, or engaging in social activity at least three times a week during the past month.
[j] Healthcare provider diagnosed sleep apnea is defined as a current doctor or other health professional diagnosis of sleep apnea.
[ǂ] Remained significant after false discovery rate correction. False discovery rate-corrected *p*-value was considered statistically significant at the $\alpha = 0.05$ level.

Supplemental Table 17B. Relative excess risk due to interaction (RERI) between perceived indoor temperature extremes and race and ethnicity, among participants who reported trouble sleeping for any reason [a], Sister Study, 2017-2019

	Indoor temperature: Too hot [a]			Indoor temperature: Too cold [b]		
	**Hispanic/Latina**	**Non-Hispanic Black**	**Non-Hispanic White**	**Hispanic/Latina**	**Non-Hispanic Black**	**Non-Hispanic White**
Weekly sleep duration [d]						
Short	0.05 (-0.71, 0.81)	-0.02 (-0.54, 0.50)	1.00 (Ref)	-0.93 (-2.07, 0.21)	0.80 (-0.56, 2.16)	1.00 (Ref)
Recommended	---	---		---	---	
Long	0.23 (-0.41, 0.87)	-0.26 (-0.62, 0.10)		-0.20 (-1.21, 0.81)	0.09 (-1.19, 1.37)	
Long sleep onset latency [e]						
Yes	0.71 (-0.05, 1.47)	0.27 (-0.14, 0.68)	1.00 (Ref)	-0.33 (-1.34, 0.68)	-0.86 (-1.85, 0.13)	1.00 (Ref)
No	---	---		---	---	
Poor sleep maintenance [f]						
Yes	-0.02 (-0.22, 0.18)	-0.11 (-0.24, 0.02)	1.00 (Ref)	0.19 (-0.18, 0.56)	0.10 (-0.26, 0.46)	1.00 (Ref)
No	---	---		---	---	
Insomnia symptoms [g]						
Yes	0.06 (-0.11, 0.23)	-0.07 (-0.19, 0.05)	1.00 (Ref)	0.15 (-0.18, 0.48)	0.12 (-0.21, 0.45)	1.00 (Ref)
No	---	---		---	---	
Sleep medication use [h]						
Yes	-0.14 (-0.49, 0.21)	-0.10 (-0.33, 0.13)	1.00 (Ref)	-0.09 (-0.62, 0.44)	-0.33 (-0.95, 0.29)	1.00 (Ref)
No	---	---		---	---	
Daytime dysfunction [i]						
Yes	-1.42 (-4.44, 1.60)	-0.04 (-1.77, 1.69)	1.00 (Ref)	8.21 (-8.76, 25.18)	6.01 (-5.59, 17.61)	1.00 (Ref)
No	---	---		---	---	
Healthcare professional diagnosed sleep apnea [j]						
Yes	0.21 (-0.43, 0.85)	0.14 (-0.22, 0.50)	1.00 (Ref)	0.94 (-0.54, 2.42)	0.60 (-0.65, 1.85)	1.00 (Ref)
No	---	---		---	---	

Note: N = 418 (1.8%) participants who reported both 'too hot' and 'too cold' temperatures were not included in this sensitivity analysis.
RERI = 0: no additive interaction; RERI >0: positive additive interaction; RERI <0: negative additive interaction.

Models are adjusted for age (continuous), age^2, annual household income (<$20,000 - $49,999, $50,000 - $99,999, ≥$100,000), marital status (married or living as though married, divorced/widowed/separated/never married), region of residence (Northeast, Midwest, South, West, and Puerto Rico), body mass index (BMI: underweight, recommended, overweight, obesity), and menopausal status (premenopausal, postmenopausal).

[a] In models for weekly sleep duration, sleep medication use, daytime dysfunction, and healthcare professional diagnosed sleep apnea, reasons for trouble sleeping for any reason other than feeling too hot or feeling too cold ≥ 3 times per week in the past month include: unable to fall asleep within 30 minutes, waking up in the middle of the night or early morning, waking up to use the bathroom, cannot breathe comfortably, coughing or snoring loudly, having bad dreams, having pain, or other non-specified reasons (n = 23,853). In models for long sleep onset latency, poor sleep maintenance, and insomnia symptoms, reasons for trouble sleeping for any reason other than feeling too hot or feeling too cold ≥ 3 times per week in the past month include waking up to use the bathroom, cannot breathe comfortably, coughing or snoring loudly, having bad dreams, having pain, or other non-specified reasons (n = 21,641).
[b] Too hot' is defined as self-reported trouble sleeping due to feeling too hot ≥ 3 times per week vs. trouble sleeping due to feeling too hot < 3 times per week and trouble sleeping due to feeling too cold < 3 times per week.
[c] 'Too cold' is defined as self-reported trouble sleeping due to feeling too cold ≥ 3 times per week vs. trouble sleeping due to feeling too hot < 3 times per week and trouble sleeping due to feeling too cold < 3 times per week.
[d] Sleep duration is based on reported bed and wake times or reported average sleep duration. Participants who reported ≤ 2 or ≥ 23 hours of sleep are excluded. Short: <7 hours; Recommended: 7-9 hours; Long ≥ 9 hours.
[e] Long sleep onset latency is defined as not falling asleep within 30 minutes at least three times a week during the past month.
[f] Poor sleep maintenance is defined as waking up in the middle of the night or early morning at least three times a week during the past month.
[g] Insomnia symptoms is defined as long sleep onset latency or poor sleep maintenance.
[h] Sleep medication use is defined as 'taking medicine (prescription or over the counter) to help you sleep' at least three times a week during the past month.
[i] Daytime dysfunction is defined as having trouble staying awake while driving, eating, or engaging in social activity at least three times a week during the past month.

[j] Healthcare provider diagnosed sleep apnea is defined as a current doctor or other health professional diagnosis of sleep apnea.

Supplemental Table 18A. Prevalence ratios (95% confidence intervals) for associations between perceived indoor temperature extremes and sleep health dimensions by annual household income, among participants who reported trouble sleeping for any reason [a], Sister Study, 2017-2019

	**Indoor temperature: Too hot [b]**				**Indoor temperature: Too cold [c]**			
	**<$20,000 - $49,999**	**$50,000 - $99,999**	**≥ $100,000**	**Wald *p*-values**	**<$20,000 - $49,999**	**$50,000 - $99,999**	**≥ $100,000**	**Wald *p*-values**
Weekly sleep duration [d]								
Short	1.02 (0.83,1.25)	**1.26 (1.07,1.48)**‡	1.12 (0.94,1.33)	0.2460	**1.44 (1.07,1.93)**‡	1.43 (0.96,2.14)	1.41 (0.92,2.17)	0.9973
Recommended	1.00 (Ref)	1.00 (Ref)	1.00 (Ref)	---	1.00 (Ref)	1.00 (Ref)	1.00 (Ref)	---
Long	**1.22 (1.04,1.43)**‡	1.13 (0.97,1.33)	**1.33 (1.15,1.54)**‡	0.3291	1.31 (0.99,1.73)	1.04 (0.68,1.58)	**1.88 (1.35,2.60)**‡	0.0730
Long sleep onset latency [e]								
Yes	**1.72 (1.49,1.98)**‡	**1.98 (1.75,2.25)**‡	**1.68 (1.46,1.95)**‡	0.1590	**1.84 (1.47,2.31)**‡	**1.85 (1.33,2.56)**‡	**1.95 (1.33,2.88)**‡	0.9654
No	1.00 (Ref)	1.00 (Ref)	1.00 (Ref)	---	1.00 (Ref)	1.00 (Ref)	1.00 (Ref)	---
Poor sleep maintenance [f]								
Yes	**1.41 (1.32,1.50)**‡	**1.42 (1.35,1.50)**‡	**1.45 (1.38,1.52)**‡	0.7509	**1.27 (1.12,1.43)**‡	**1.31 (1.14,1.52)**‡	**1.18 (1.00,1.39)**‡	0.6297
No	1.00 (Ref)	1.00 (Ref)	1.00 (Ref)	---	1.00 (Ref)	1.00 (Ref)	1.00 (Ref)	---
Insomnia symptoms [g]								
Yes	**1.39 (1.31,1.47)**‡	**1.42 (1.36,1.49)**‡	**1.43 (1.37,1.50)**‡	0.6617	**1.24 (1.11,1.39)**‡	**1.27 (1.11,1.46)**‡	**1.21 (1.03,1.41)**‡	0.8874
No	1.00 (Ref)	1.00 (Ref)	1.00 (Ref)	---	1.00 (Ref)	1.00 (Ref)	1.00 (Ref)	---
Sleep medication use [h]								
Yes	**1.26 (1.09,1.45)**‡	**1.21 (1.07,1.37)**‡	1.10 (0.97,1.25)	0.3580	**1.39 (1.11,1.75)**‡	1.01 (0.71,1.43)	**1.62 (1.23,2.15)**‡	0.1151
No	1.00 (Ref)	1.00 (Ref)	1.00 (Ref)	---	1.00 (Ref)	1.00 (Ref)	1.00 (Ref)	---
Daytime dysfunction [i]								
Yes	1.47 (0.76,2.84)	**2.18 (1.17,4.05)**‡	1.67 (0.89,3.13)	0.6793	**2.66 (1.10,6.42)**‡	**9.17 (4.23,19.86)**‡	**5.64 (2.09,15.19)**‡	0.1176
No	1.00 (Ref)	1.00 (Ref)	1.00 (Ref)	---	1.00 (Ref)	1.00 (Ref)	1.00 (Ref)	---
Healthcare professional diagnosed sleep apnea [j]								
Yes	1.18 (0.97,1.44)	1.13 (0.95,1.35)	1.16 (0.96,1.39)	0.9457	**1.56 (1.16,2.09)**‡	**1.84 (1.27,2.66)**‡	**1.63 (1.03,2.57)**‡	0.7786
No	1.00 (Ref)	1.00 (Ref)	1.00 (Ref)	---	1.00 (Ref)	1.00 (Ref)	1.00 (Ref)	---

Note: N = 418 (1.8%) participants who reported both 'too hot' and 'too cold' temperatures were not included in this sensitivity analysis.

Models are adjusted for age (continuous), age^2, race and ethnicity (Hispanic/Latina, non-Hispanic Black, non-Hispanic White), marital status (married or living as though married, divorced/widowed/separated/never married), region of residence (Northeast, Midwest, South, West, and Puerto Rico), body mass index (BMI: underweight, recommended, overweight, obesity), and menopausal status (premenopausal, postmenopausal).

[a] In models for weekly sleep duration, sleep medication use, daytime dysfunction, and healthcare professional diagnosed sleep apnea, reasons for trouble sleeping for any reason other than feeling too hot or feeling too cold ≥ 3 times per week in the past month include: unable to fall asleep within 30 minutes, waking up in the middle of the night or early morning, waking up to use the bathroom, cannot breathe comfortably, coughing or snoring loudly, having bad dreams, having pain, or other non-specified reasons (n = 23,853). In models for long sleep onset latency, poor sleep maintenance, and insomnia symptoms, reasons for trouble sleeping for any reason other than feeling too hot or feeling too cold ≥ 3 times per week in the past month include waking up to use the bathroom, cannot breathe comfortably, coughing or snoring loudly, having bad dreams, having pain, or other non-specified reasons (n = 21,641).
[b] Too hot' is defined as self-reported trouble sleeping due to feeling too hot ≥ 3 times per week vs. trouble sleeping due to feeling too hot < 3 times per week and trouble sleeping due to feeling too cold < 3 times per week.
[c] 'Too cold' is defined as self-reported trouble sleeping due to feeling too cold ≥ 3 times per week vs. trouble sleeping due to feeling too hot < 3 times per week and trouble sleeping due to feeling too cold < 3 times per week.
[d] Sleep duration is based on reported bed and wake times or reported average sleep duration. Participants who reported ≤ 2 or ≥ 23 hours of sleep are excluded. Short: <7 hours; Recommended: 7-9 hours; Long ≥ 9 hours.
[e] Long sleep onset latency is defined as not falling asleep within 30 minutes at least three times a week during the past month.
[f] Poor sleep maintenance is defined as waking up in the middle of the night or early morning at least three times a week during the past month.
[g] Insomnia symptoms is defined as long sleep onset latency or poor sleep maintenance.
[h] Sleep medication use is defined as 'taking medicine (prescription or over the counter) to help you sleep' at least three times a week during the past month.

[i] Daytime dysfunction is defined as having trouble staying awake while driving, eating, or engaging in social activity at least three times a week during the past month.
[j] Healthcare provider diagnosed sleep apnea is defined as a current doctor or other health professional diagnosis of sleep apnea.
[ł] Remained significant after false discovery rate correction. False discovery rate-corrected *p*-value was considered statistically significant at the $\alpha = 0.05$ level.

Supplemental Table 18B. Relative excess risk due to interaction (RERI) between perceived temperature extremes and annual household income, among participants who reported trouble sleeping for any reason [a], Sister Study, 2017-2019

	Indoor temperature: Too hot [b]			Indoor temperature: Too cold [c]		
	**< $20,000 - $49,999**	**$50,000 - $99,999**	**≥ $100,000**	**< $20,000 - $49,999**	**$50,000 - $99,999**	**≥ $100,000**
Weekly sleep duration [d]						
Short	-0.09 (-0.40, 0.22)	0.16 (-0.11, 0.43)	1.00 (Ref)	0.15 (-0.65, 0.95)	0.05 (-0.80, 0.90)	1.00 (Ref)
Recommended	---	---		---	---	
Long	-0.06 (-0.40, 0.28)	-0.28 (-0.56, 0.00)		-0.43 (-1.22, 0.36)	**-0.83 (-1.62, -0.04)**	
Long sleep onset latency [e]						
Yes	**0.39 (0.01, 0.77)**	**0.49 (0.16, 0.82)**	1.00 (Ref)	0.35 (-0.62, 1.32)	0.07 (-0.97, 1.11)	1.00 (Ref)
No	---	---		---	---	
Poor sleep maintenance [f]						
Yes	-0.04 (-0.12, 0.04)	-0.03 (-0.10, 0.04)	1.00 (Ref)	0.09 (-0.15, 0.33)	0.13 (-0.14, 0.40)	1.00 (Ref)
No	---	---		---	---	
Insomnia symptoms [g]						
Yes	-0.01 (-0.07, 0.05)	-0.01 (-0.06, 0.04)	1.00 (Ref)	0.04 (-0.19, 0.27)	0.07 (-0.18, 0.32)	1.00 (Ref)
No	---	---		---	---	
Sleep medication use [h]						
Yes	0.20 (-0.04, 0.44)	0.12 (-0.08, 0.32)	1.00 (Ref)	-0.16 (-0.74, 0.42)	**-0.61 (-1.20, -0.02)**	1.00 (Ref)
No	---	---		---	---	
Daytime dysfunction [i]						
Yes	0.12 (-1.72, 1.96)	0.46 (-1.03, 1.95)	1.00 (Ref)	-1.81 (-8.52, 4.90)	3.35 (-5.15, 11.85)	1.00 (Ref)
No	---	---		---	---	
Healthcare professional diagnosed sleep apnea [j]						
Yes	0.04 (-0.28, 0.36)	-0.02 (-0.30, 0.26)	1.00 (Ref)	-0.02 (-0.90, 0.86)	0.24 (-0.76, 1.24)	1.00 (Ref)
No	---	---		---	---	

Note: N = 418 (1.8%) participants who reported both 'too hot' and 'too cold' temperatures were not included in this sensitivity analysis.
RERI = 0: no additive interaction; RERI >0: positive additive interaction; RERI <0: negative additive interaction.

Models are adjusted for age (continuous), age^2, race and ethnicity (Hispanic/Latina, non-Hispanic Black, non-Hispanic White), marital status (married or living as though married, divorced/widowed/separated/never married), region of residence (Northeast, Midwest, South, West, and Puerto Rico), body mass index (BMI: underweight, recommended, overweight, obesity), and menopausal status (premenopausal, postmenopausal).

[a] In models for weekly sleep duration, sleep medication use, daytime dysfunction, and healthcare professional diagnosed sleep apnea, reasons for trouble sleeping for any reason other than feeling too hot or feeling too cold ≥ 3 times per week in the past month include: unable to fall asleep within 30 minutes, waking up in the middle of the night or early morning, waking up to use the bathroom, cannot breathe comfortably, coughing or snoring loudly, having bad dreams, having pain, or other non-specified reasons (n = 23,853). In models for long sleep onset latency, poor sleep maintenance, and insomnia symptoms, reasons for trouble sleeping for any reason other than feeling too hot or feeling too cold ≥ 3 times per week in the past month include waking up to use the bathroom, cannot breathe comfortably, coughing or snoring loudly, having bad dreams, having pain, or other non-specified reasons (n = 21,641).
[b] Too hot' is defined as self-reported trouble sleeping due to feeling too hot ≥ 3 times per week vs. trouble sleeping due to feeling too hot < 3 times per week and trouble sleeping due to feeling too cold < 3 times per week.
[c] 'Too cold' is defined as self-reported trouble sleeping due to feeling too cold ≥ 3 times per week vs. trouble sleeping due to feeling too hot < 3 times per week and trouble sleeping due to feeling too cold < 3 times per week.

[d] Sleep duration is based on reported bed and wake times or reported average sleep duration. Participants who reported ≤ 2 or ≥ 23 hours of sleep are excluded. Short: <7 hours; Recommended: 7-9 hours; Long ≥ 9 hours.
[e] Long sleep onset latency is defined as not falling asleep within 30 minutes at least three times a week during the past month.
[f] Poor sleep maintenance is defined as waking up in the middle of the night or early morning at least three times a week during the past month.
[g] Insomnia symptoms is defined as long sleep onset latency or poor sleep maintenance.
[h] Sleep medication use is defined as 'taking medicine (prescription or over the counter) to help you sleep' at least three times a week during the past month.
[i] Daytime dysfunction is defined as having trouble staying awake while driving, eating, or engaging in social activity at least three times a week during the past month.
[j] Healthcare provider diagnosed sleep apnea is defined as a current doctor or other health professional diagnosis of sleep apnea.

Supplemental Table 19A. Prevalence ratios (95% confidence intervals) for associations between perceived indoor temperature extremes and sleep health dimensions by region of residence, among participants who reported trouble sleeping for any reason [a], Sister Study, 2017-2019

	Indoor temperature: Too hot [b]						Indoor temperature: Too cold [c]					
	**Northeast**	**Midwest**	**South**	**West**	**Puerto Rico**	**Wald *p*-values**	**Northeast**	**Midwest**	**South**	**West**	**Puerto Rico**	**Wald *p*-values**
Weekly sleep duration [d]												
Short	0.96 (0.75,1.24)	**1.44 (1.19,1.74)‡**	1.01 (0.86,1.20)	1.15 (0.91,1.46)	**2.33 (1.18,4.58)‡**	**0.0075**	**2.04 (1.37,3.02)‡**	**1.52 (1.01,2.30)‡**	1.38 (0.96,1.99)	1.23 (0.73,2.05)	0.55 (0.14,2.13)	0.2669
Recommended	1.00 (Ref)	1.00 (Ref)	1.00 (Ref)	1.00 (Ref)	1.00 (Ref)	---	1.00 (Ref)	1.00 (Ref)	1.00 (Ref)	1.00 (Ref)	1.00 (Ref)	---
Long	1.14 (0.90,1.43)	**1.24 (1.03,1.48)‡**	**1.16 (1.00,1.35)‡**	**1.33 (1.12,1.58)‡**	**3.23 (1.75,5.95)‡**	**0.0231**	1.28 (0.72,2.27)	1.06 (0.67,1.67)	1.35 (0.98,1.86)	**1.73 (1.24,2.40)‡**	1.29 (0.49,3.39)	0.5237
Long sleep onset latency [e]												
Yes	**1.71 (1.40,2.09)‡**	**1.63 (1.38,1.93)‡**	**2.02 (1.79,2.28)‡**	**1.74 (1.46,2.07)‡**	1.24 (0.58,2.64)	0.1880	1.37 (0.78,2.38)	**2.17 (1.61,2.93)‡**	**1.67 (1.21,2.31)‡**	**2.06 (1.48,2.89)‡**	**1.97 (1.16,3.36)‡**	0.5585
No	1.00 (Ref)	1.00 (Ref)	1.00 (Ref)	1.00 (Ref)	1.00 (Ref)	---	1.00 (Ref)	1.00 (Ref)	1.00 (Ref)	1.00 (Ref)	1.00 (Ref)	---
Poor sleep maintenance [f]												
Yes	**1.52 (1.42,1.62)‡**	**1.40 (1.31,1.49)‡**	**1.43 (1.36,1.51)‡**	**1.39 (1.30,1.48)‡**	**1.63 (1.16,2.29)‡**	0.2606	1.06 (0.81,1.39)	**1.41 (1.23,1.62)‡**	**1.23 (1.06,1.43)‡**	1.16 (0.97,1.38)	**1.70 (1.23,2.34)‡**	0.0770
No	1.00 (Ref)	1.00 (Ref)	1.00 (Ref)	1.00 (Ref)	1.00 (Ref)	---	1.00 (Ref)	1.00 (Ref)	1.00 (Ref)	1.00 (Ref)	1.00 (Ref)	---
Insomnia symptoms [g]												
Yes	**1.49 (1.40,1.58)‡**	**1.39 (1.31,1.47)‡**	**1.42 (1.35,1.49)‡**	**1.40 (1.33,1.48)‡**	**1.57 (1.17,2.12)‡**	0.4580	1.04 (0.81,1.35)	**1.37 (1.20,1.56)‡**	**1.25 (1.09,1.43)‡**	1.13 (0.96,1.34)	**1.63 (1.24,2.16)‡**	0.0729
No	1.00 (Ref)	1.00 (Ref)	1.00 (Ref)	1.00 (Ref)	1.00 (Ref)	---	1.00 (Ref)	1.00 (Ref)	1.00 (Ref)	1.00 (Ref)	1.00 (Ref)	---
Sleep medication use [h]												
Yes	**1.26 (1.03,1.53)‡**	1.10 (0.94,1.28)	**1.27 (1.12,1.43)‡**	1.09 (0.93,1.27)	1.24 (0.63,2.44)	0.4515	**1.78 (1.21,2.62)‡**	**1.49 (1.11,2.01)‡**	1.20 (0.89,1.63)	1.01 (0.69,1.47)	**1.91 (1.13,3.23)‡**	0.1408
No	1.00 (Ref)	1.00 (Ref)	1.00 (Ref)	1.00 (Ref)	1.00 (Ref)	---	1.00 (Ref)	1.00 (Ref)	1.00 (Ref)	1.00 (Ref)	1.00 (Ref)	---
Daytime dysfunction [i]												
Yes	---	---	---	---	---	---	---	---	---	---	---	---
No	1.00 (Ref)	1.00 (Ref)	1.00 (Ref)	1.00 (Ref)	1.00 (Ref)	---	1.00 (Ref)	1.00 (Ref)	1.00 (Ref)	1.00 (Ref)	1.00 (Ref)	---
Healthcare professional diagnosed sleep apnea [j]												
Yes	1.22 (0.92,1.61)	1.11 (0.89,1.39)	**1.26 (1.06,1.49)‡**	0.98 (0.78,1.24)	**3.86 (1.13,13.20)‡**	0.1482	1.61 (0.92,2.81)	0.90 (0.49,1.66)	**1.91 (1.40,2.61)‡**	**1.75 (1.17,2.61)‡**	**5.52 (1.95,15.59)‡**	**0.0467**
No	1.00 (Ref)	1.00 (Ref)	1.00 (Ref)	1.00 (Ref)	1.00 (Ref)	---	1.00 (Ref)	1.00 (Ref)	1.00 (Ref)	1.00 (Ref)	1.00 (Ref)	---

Note: N = 418 (1.8%) participants who reported both 'too hot' and 'too cold' temperatures were not included in this sensitivity analysis.

Models are adjusted age (continuous), age^2, race and ethnicity (Hispanic/Latina, non-Hispanic Black, non-Hispanic White), annual household income (<\$20,000 - \$49,999, \$50,000 - \$99,999, ≥\$100,000), marital status (married or living as though married, divorced/widowed/separated/never married), body mass index (BMI: underweight, recommended, overweight, obesity), and menopausal status (premenopausal, postmenopausal).

[a] In models for weekly sleep duration, sleep medication use, daytime dysfunction, and healthcare professional diagnosed sleep apnea, reasons for trouble sleeping for any reason other than feeling too hot or feeling too cold ≥ 3 times per week in the past month include: unable to fall asleep within 30 minutes, waking up in the middle of the night or early morning, waking up to use the bathroom, cannot breathe comfortably, coughing or snoring loudly, having bad dreams, having pain, or other non-specified reasons (n = 23,853). In models for long sleep onset latency, poor sleep maintenance, and insomnia symptoms, reasons for trouble sleeping for any reason other than feeling too hot or feeling too cold ≥ 3 times per week in the past month include waking up to use the bathroom, cannot breathe comfortably, coughing or snoring loudly, having bad dreams, having pain, or other non-specified reasons (n = 21,641).

[b] Too hot' is defined as self-reported trouble sleeping due to feeling too hot ≥ 3 times per week vs. trouble sleeping due to feeling too hot < 3 times per week and trouble sleeping due to feeling too cold < 3 times per week.

[c] 'Too cold' is defined as self-reported trouble sleeping due to feeling too cold ≥ 3 times per week vs. trouble sleeping due to feeling too hot < 3 times per week and trouble sleeping due to feeling too cold < 3 times per week.
[d] Sleep duration is based on reported bed and wake times or reported average sleep duration. Participants who reported ≤ 2 or ≥ 23 hours of sleep are excluded. Short: <7 hours; Recommended: 7-9 hours; Long ≥ 9 hours.
[e] Long sleep onset latency is defined as not falling asleep within 30 minutes at least three times a week during the past month.
[f] Poor sleep maintenance is defined as waking up in the middle of the night or early morning at least three times a week during the past month.
[g] Insomnia symptoms is defined as long sleep onset latency or poor sleep maintenance.
[h] Sleep medication use is defined as 'taking medicine (prescription or over the counter) to help you sleep' at least three times a week during the past month.
[i] Daytime dysfunction is defined as having trouble staying awake while driving, eating, or engaging in social activity at least three times a week during the past month.
[j] Healthcare provider diagnosed sleep apnea is defined as a current doctor or other health professional diagnosis of sleep apnea.
[ł] Remained significant after false discovery rate correction. False discovery rate-corrected *p*-value was considered statistically significant at the α = 0.05 level.

Supplemental Table 19B. Relative excess risk due to interaction (RERI) between perceived temperature extremes and region of residence, among participants who reported trouble sleeping for any reason [a], Sister Study, 2017-2019

	Indoor temperature: Too hot [b]					Indoor temperature: Too cold [c]				
	**Northeast**	**Midwest**	**South**	**West**	**Puerto Rico**	**Northeast**	**Midwest**	**South**	**West**	**Puerto Rico**
Weekly sleep duration [d]										
Short	-0.06 (-0.40, 0.28)	**0.39 (0.11, 0.67)**		0.13 (-0.17, 0.43)	1.29 (-0.14, 2.72)	0.89 (-0.19, 1.97)	0.10 (-0.66, 0.86)		-0.17 (-0.95, 0.61)	-0.81 (-1.72, 0.10)
Recommended	---	---	1.00 (Ref)	---	---	---	---	1.00 (Ref)	---	---
Long	-0.03 (-0.33, 0.27)	0.05 (-0.20, 0.30)		0.15 (-0.12, 0.42)	**1.22 (0.18, 2.26)**	-0.08 (-0.90, 0.74)	-0.30 (-0.91, 0.31)		0.34 (-0.34, 1.02)	-0.17 (-1.03, 0.69)
Long sleep onset latency [e]										
Yes	-0.31 (-0.69, 0.07)	**-0.43 (-0.75, -0.11)**	1.00 (Ref)	-0.33 (-0.67, 0.01)	-0.79 (-1.69, 0.11)	-0.31 (-1.22, 0.60)	0.41 (-0.38, 1.20)	1.00 (Ref)	0.32 (-0.51, 1.15)	0.34 (-0.78, 1.46)
No	---	---		---	---	---	---		---	---
Poor sleep maintenance [f]										
Yes	0.10 (0.00, 0.20)	-0.04 (-0.13, 0.05)	1.00 (Ref)	-0.03 (-0.12, 0.06)	0.15 (-0.29, 0.59)	-0.17 (-0.51, 0.17)	0.17 (-0.09, 0.43)	1.00 (Ref)	-0.07 (-0.34, 0.20)	0.40 (-0.05, 0.85)
No	---	---		---	---	---	---		---	---
Insomnia symptoms [g]										
Yes	0.08 (-0.01, 0.17)	-0.04 (-0.12, 0.04)	1.00 (Ref)	-0.01 (-0.09, 0.07)	0.10 (-0.26, 0.46)	-0.21 (-0.52, 0.10)	0.11 (-0.12, 0.34)	1.00 (Ref)	-0.12 (-0.37,0.13)	0.33 (-0.04, 0.70)
No										
Sleep medication use [h]										
Yes	-0.06 (-0.31, 0.19)	-0.18 (-0.39, 0.03)	1.00 (Ref)	-0.18 (-0.40, 0.04)	0.06 (-1.07, 1.19)	0.45 (-0.22, 1.12)	0.25 (-0.29, 0.79)	1.00 (Ref)	-0.20 (-0.73, 0.33)	1.06 (-0.26, 2.38)
No	---	---		---	---	---	---		---	---
Daytime dysfunction [i]										
Yes	---	---	1.00 (Ref)	---	---	---	---	1.00 (Ref)	---	---
No	---	---		---	---	---	---		---	---
Healthcare professional diagnosed sleep apnea [j]										
Yes	-0.06 (-0.42, 0.30)	-0.15 (-0.45, 0.15)	1.00 (Ref)	-0.28 (-0.62, 0.06)	0.78 (-0.67, 2.23)	-0.37 (-1.36, 0.62)	**-1.01 (-1.80, -0.22)**	1.00 (Ref)	-0.00 (-1.01, 1.01)	0.76 (-0.93, 2.45)
No	---	---		---	---	---	---		---	---

Note: N = 418 (1.8%) participants who reported both 'too hot' and 'too cold' temperatures were not included in this sensitivity analysis.
RERI = 0: no additive interaction; RERI >0: positive additive interaction; RERI <0: negative additive interaction.

Models are adjusted for age (continuous), age^2, race and ethnicity (Hispanic/Latina, non-Hispanic Black, non-Hispanic White), annual household income (<$20,000 - $49,999, $50,000 - $99,999, ≥$100,000), marital status (married or living as though married, divorced/widowed/separated/never married), body mass index (BMI: underweight, recommended, overweight, obesity), and menopausal status (premenopausal, postmenopausal).

[a] In models for weekly sleep duration, sleep medication use, daytime dysfunction, and healthcare professional diagnosed sleep apnea, reasons for trouble sleeping for any reason other than feeling too hot or feeling too cold ≥ 3 times per week in the past month include: unable to fall asleep within 30 minutes, waking up in the middle of the night or early morning, waking up to use the bathroom, cannot breathe comfortably, coughing or snoring loudly, having bad dreams, having pain, or other non-specified reasons (n = 23,853). In models for long sleep onset latency, poor sleep maintenance, and insomnia symptoms, reasons for trouble sleeping for any reason other than feeling too hot or feeling too cold ≥ 3 times per week in the past month include waking up to use the bathroom, cannot breathe comfortably, coughing or snoring loudly, having bad dreams, having pain, or other non-specified reasons (n = 21,641).
[b] Too hot' is defined as self-reported trouble sleeping due to feeling too hot ≥ 3 times per week vs. trouble sleeping due to feeling too hot < 3 times per week and trouble sleeping due to feeling too cold < 3 times per week.
[c] 'Too cold' is defined as self-reported trouble sleeping due to feeling too cold ≥ 3 times per week vs. trouble sleeping due to feeling too hot < 3 times per week and trouble sleeping due to feeling too cold < 3 times per week.
[d] Sleep duration is based on reported bed and wake times or reported average sleep duration. Participants who reported ≤ 2 or ≥ 23 hours of sleep are excluded. Short: <7 hours; Recommended: 7-9 hours; Long ≥ 9 hours.
[e] Long sleep onset latency is defined as not falling asleep within 30 minutes at least three times a week during the past month.
[f] Poor sleep maintenance is defined as waking up in the middle of the night or early morning at least three times a week during the past month.
[g] Insomnia symptoms is defined as long sleep onset latency or poor sleep maintenance.
[h] Sleep medication use is defined as 'taking medicine (prescription or over the counter) to help you sleep' at least three times a week during the past month.
[i] Daytime dysfunction is defined as having trouble staying awake while driving, eating, or engaging in social activity at least three times a week during the past month.

[j] Healthcare provider diagnosed sleep apnea is defined as a current doctor or other health professional diagnosis of sleep apnea.

Supplemental Table 20A. Prevalence ratios (95% confidence intervals) for associations between perceived indoor temperature extremes and sleep health dimensions by menopausal status, among participants who reported trouble sleeping for any reason [a], Sister Study, 2017-2019

	Indoor temperature: Too hot [b]			Indoor temperature: Too cold [c]		
	**Premenopausal**	**Postmenopausal**	**Wald *p*-values**	**Premenopausal**	**Postmenopausal**	**Wald *p*-values**
Weekly sleep duration [d]						
Short	1.13 (0.75,1.70)	**1.14 (1.03,1.27)‡**	0.9567	1.55 (0.74,3.22)	**1.42 (1.14,1.77)‡**	0.8276
Recommended	1.0 (Ref)	1.0 (Ref)	---	1.0 (Ref)	1.0 (Ref)	---
Long	1.15 (0.72,1.83)	**1.25 (1.13,1.37)‡**	0.7407	1.35 (0.47,3.84)	**1.36 (1.12,1.66)‡**	0.9855
Long sleep onset latency [e]						
Yes	**2.16 (1.45,3.21)‡**	**1.79 (1.65,1.95)‡**	0.3677	0.94 (0.25,3.60)	**1.90 (1.61,2.25)‡**	0.3085
No	1.0 (Ref)	1.0 (Ref)	---	1.0 (Ref)	1.0 (Ref)	---
Poor sleep maintenance [f]						
Yes	**1.45 (1.25,1.68)‡**	**1.43 (1.38,1.47)‡**	0.8261	**1.40 (1.00,1.96)‡**	**1.25 (1.15,1.36)‡**	0.5299
No	1.0 (Ref)	1.0 (Ref)	---	1.0 (Ref)	1.0 (Ref)	---
Insomnia symptoms [g]						
Yes	**1.32 (1.19,1.47)‡**	**1.35 (1.32,1.39)‡**	0.6549	1.18 (0.88,1.59)	**1.21 (1.12,1.29)‡**	0.9033
No	1.0 (Ref)	1.0 (Ref)	---	1.0 (Ref)	1.0 (Ref)	---
Sleep medication use [h]						
Yes	1.03 (0.70,1.50)	**1.19 (1.10,1.28)‡**	0.4659	1.50 (0.71,3.16)	**1.33 (1.13,1.56)‡**	0.7535
No	1.0 (Ref)	1.0 (Ref)	---	1.0 (Ref)	1.0 (Ref)	---
Daytime dysfunction [i]						
Yes	0.94 (0.11,8.39)	**1.79 (1.23,2.61)‡**	0.5693	**15.61 (2.92,83.52)‡**	**4.43 (2.54,7.74)‡**	0.1706
No	1.0 (Ref)	1.0 (Ref)	---	1.0 (Ref)	1.0 (Ref)	---
Healthcare professional diagnosed sleep apnea [j]						
Yes	0.87 (0.47,1.60)	**1.17 (1.04,1.31)‡**	0.3517	0.61 (0.09,4.03)	**1.69 (1.38,2.08)‡**	0.2919
No	1.0 (Ref)	1.0 (Ref)	---	1.0 (Ref)	1.0 (Ref)	---

Note: N = 418 (1.8%) participants who reported both 'too hot' and 'too cold' temperatures were not included in this sensitivity analysis.

Models are adjusted for age (continuous), age^2, race and ethnicity (Hispanic/Latina, non-Hispanic Black, non-Hispanic White), income (<$20,000 - $49,999, $50,000 - $99,999, ≥$100,000), marital status (married or living as though married, divorced/widowed/separated/never married), region of residence (Northeast, Midwest, South, West, and Puerto Rico), and body mass index (BMI: underweight, recommended, overweight, obesity).

[a] In models for weekly sleep duration, sleep medication use, daytime dysfunction, and healthcare professional diagnosed sleep apnea, reasons for trouble sleeping for any reason other than feeling too hot or feeling too cold ≥ 3 times per week in the past month include: unable to fall asleep within 30 minutes, waking up in the middle of the night or early morning, waking up to use the bathroom, cannot breathe comfortably, coughing or snoring loudly, having bad dreams, having pain, or other non-specified reasons (n = 23,853). In models for long sleep onset latency, poor sleep maintenance, and insomnia symptoms, reasons for trouble sleeping for any reason other than feeling too hot or feeling too cold ≥ 3 times per week in the past month include waking up to use the bathroom, cannot breathe comfortably, coughing or snoring loudly, having bad dreams, having pain, or other non-specified reasons (n = 21,641).
[b] Too hot' is defined as self-reported trouble sleeping due to feeling too hot ≥ 3 times per week vs. trouble sleeping due to feeling too hot < 3 times per week and trouble sleeping due to feeling too cold < 3 times per week.
[c] 'Too cold' is defined as self-reported trouble sleeping due to feeling too cold ≥ 3 times per week vs. trouble sleeping due to feeling too hot < 3 times per week and trouble sleeping due to feeling too cold < 3 times per week.
[d] Sleep duration is based on reported bed and wake times or reported average sleep duration. Participants who reported ≤ 2 or ≥ 23 hours of sleep are excluded. Short: <7 hours; Recommended: 7-9 hours; Long ≥ 9 hours.
[e] Long sleep onset latency is defined as not falling asleep within 30 minutes at least three times a week during the past month.
[f] Poor sleep maintenance is defined as waking up in the middle of the night or early morning at least three times a week during the past month.
[g] Insomnia symptoms is defined as long sleep onset latency or poor sleep maintenance.
[h] Sleep medication use is defined as 'taking medicine (prescription or over the counter) to help you sleep' at least three times a week during the past month.
[i] Daytime dysfunction is defined as having trouble staying awake while driving, eating, or engaging in social activity at least three times a week during the past month.
[j] Healthcare provider diagnosed sleep apnea is defined as a current doctor or other health professional diagnosis of sleep apnea.

[ǀ] Remained significant after false discovery rate correction. False discovery rate-corrected *p*-value was considered statistically significant at the $\alpha = 0.05$ level.

Supplemental Table 20B. Relative excess risk due to interaction (RERI) between perceived temperature extremes and menopausal status, among participants who reported trouble sleeping for any reason [a], Sister Study, 2017-2019

	Indoor temperature: Too hot [b]		Indoor temperature: Too cold [c]	
	Premenopausal	Postmenopausal	Premenopausal	Postmenopausal
Weekly sleep duration [d]				
Short		0.01 (-0.42, 0.44)		-0.12 (-1.26, 1.02)
Recommended	1.0 (Ref)	---	1.0 (Ref)	---
Long		-0.04 (-0.53, 0.45)		-0.08 (-1.52, 1.36)
Long sleep onset latency [e]				
Yes	1.0 (Ref)	-0.15 (-0.81, 0.51)	1.0 (Ref)	**1.32 (0.04, 2.60)**
No		---		---
Poor sleep maintenance [f]				
Yes	1.0 (Ref)	N/A*	1.0 (Ref)	-0.14 (-0.58, 0.30)
No		---		---
Insomnia symptoms [g]				
Yes	1.0 (Ref)	N/A*	1.0 (Ref)	-0.07 (-0.48, 0.34)
No		---		---
Sleep medication use [h]				
Yes	1.0 (Ref)	0.16 (-0.20, 0.52)	1.0 (Ref)	-0.18 (-1.29, 0.93)
No		---		---
Daytime dysfunction [i]				
Yes	1.0 (Ref)	1.21 (-0.97, 3.39)	1.0 (Ref)	-9.57 (-33.73, 14.59)
No		---		---
Healthcare professional diagnosed sleep apnea [j]				
Yes	1.0 (Ref)	0.33 (-0.14, 0.80)	1.0 (Ref)	**1.26 (0.08, 2.44)**
No		---		---

Note: N = 418 (1.8%) participants who reported both 'too hot' and 'too cold' temperatures were not included in this sensitivity analysis.
RERI = 0: no additive interaction; RERI >0: positive additive interaction; RERI <0: negative additive interaction.

Models are adjusted for age (continuous), age^2, race and ethnicity (Hispanic/Latina, non-Hispanic Black, non-Hispanic White), income (<$20,000 - $49,999, $50,000 - $99,999, ≥$100,000), marital status (married or living as though married, divorced/widowed/separated/never married), region of residence (Northeast, Midwest, South, West, and Puerto Rico), and body mass index (BMI: underweight, recommended, overweight, obesity).

[a] In models for weekly sleep duration, sleep medication use, daytime dysfunction, and healthcare professional diagnosed sleep apnea, reasons for trouble sleeping for any reason other than feeling too hot or feeling too cold ≥ 3 times per week in the past month include: unable to fall asleep within 30 minutes, waking up in the middle of the night or early morning, waking up to use the bathroom, cannot breathe comfortably, coughing or snoring loudly, having bad dreams, having pain, or other non-specified reasons (n = 23,853). In models for long sleep onset latency, poor sleep maintenance, and insomnia symptoms, reasons for trouble sleeping for any reason other than feeling too hot or feeling too cold ≥ 3 times per week in the past month include waking up to use the bathroom, cannot breathe comfortably, coughing or snoring loudly, having bad dreams, having pain, or other non-specified reasons (n = 21,641).
[b] Too hot' is defined as self-reported trouble sleeping due to feeling too hot ≥ 3 times per week vs. trouble sleeping due to feeling too hot < 3 times per week and trouble sleeping due to feeling too cold < 3 times per week.
[c] 'Too cold' is defined as self-reported trouble sleeping due to feeling too cold ≥ 3 times per week vs. trouble sleeping due to feeling too hot < 3 times per week and trouble sleeping due to feeling too cold < 3 times per week.

[d] Sleep duration is based on reported bed and wake times or reported average sleep duration. Participants who reported ≤ 2 or ≥ 23 hours of sleep are excluded. Short: <7 hours; Recommended: 7-9 hours; Long ≥ 9 hours.
[e] Long sleep onset latency is defined as not falling asleep within 30 minutes at least three times a week during the past month.
[f] Poor sleep maintenance is defined as waking up in the middle of the night or early morning at least three times a week during the past month.
[g] Insomnia symptoms is defined as long sleep onset latency or poor sleep maintenance.
[h] Sleep medication use is defined as 'taking medicine (prescription or over the counter) to help you sleep' at least three times a week during the past month.
[i] Daytime dysfunction is defined as having trouble staying awake while driving, eating, or engaging in social activity at least three times a week during the past month.
[j] Healthcare provider diagnosed sleep apnea is defined as a current doctor or other health professional diagnosis of sleep apnea.
* RERI estimates are invalid because the covariances between 'too hot' and postmenopausal status do not fall within the plausible bounds of the 95% CI bound.
